# Local graph estimation with pathwise false discovery control

**DOI:** 10.1038/s41467-026-72796-9

**Published:** 2026-05-12

**Authors:** Omar Melikechi, David B. Dunson, Noureddine Melikechi, Jeffrey W. Miller

**Affiliations:** 1 Department of Statistical Science, Duke University, Durham, NC USA; 2 Kennedy College of Sciences, University of Massachusetts Lowell, Lowell, MA USA; 3 Department of Biostatistics, Harvard T.H. Chan School of Public Health, Boston, MA USA

**Keywords:** Statistical methods, Probabilistic data networks, Cancer genomics, Machine learning, Cancer epidemiology

## Abstract

Many datasets include a small set of variables, such as biomarkers or clinical outcomes, whose relationships to the broader system are of primary scientific interest. Estimating the full network of inter-variable relationships in such settings often obscures local structures around these targets, limiting interpretability. To address this fundamental problem, we introduce local graph estimation, a statistical framework for inferring substructures around target variables. We show that traditional graph estimation methods often fail to recover local structure, and present pathwise feature selection (PFS) as an effective alternative. PFS estimates local subgraphs by iteratively applying feature selection and propagating uncertainty along network paths, providing rigorous finite-sample false discovery control even in settings with mixed variable types and nonlinear dependencies. In four distinct applications spanning environmental and public health, multiomics, brain connectomics, and single-nucleus RNA sequencing, PFS recovers interpretable networks consistent with domain knowledge, highlighting its ability to uncover established mechanisms and generate novel hypotheses.

## Abstract (web summary)

Local graph estimation can be used to recover interpretable substructures around target variables in large datasets. Here, authors propose pathwise feature selection (PFS) with The editor’s summary is accurate. finite-sample false discovery control, outperforming global methods across diverse applications.

## Introduction

Datasets increasingly contain hundreds or thousands of variables, often of different types or *modalities*. In many applications, however, the objective is not to study the full system of inter-variable relationships but to understand a small number of *target variables*, such as biomarkers or clinical outcomes. In these settings, investigators aim to uncover local substructures within the system, such as paths or clusters of covariates, that are meaningfully related to the targets of interest.

Despite substantial advances in high-dimensional and multimodal data analysis in recent years, many approaches are misaligned with local structure learning. Popular dimension reduction algorithms such as iCluster and Multi-Omics Factor Analysis (MOFA) focus on low-dimensional representations that explain sources of variation across the full dataset, but are likely to miss local structures if they account for only a small fraction of overall variation in the data^1–3^. Predictive modeling is another important component of data analysis, but predictive accuracy alone does not provide a principled basis for structure learning^4,5^.

A popular framework that focuses directly on structure learning is *graph estimation*, which represents variables as nodes in a graph and inter-variable relationships as edges between them^6,7^. In this work, we focus on *conditional independence graphs* (CIGs), where edges encode conditional dependencies between variables. Graphical representations are particularly appealing for interpretation, as they formalize direct associations and organize indirect relationships in a way that can be visualized and used to reason about both individual variables and the system as a whole.

**Fig. 1:**
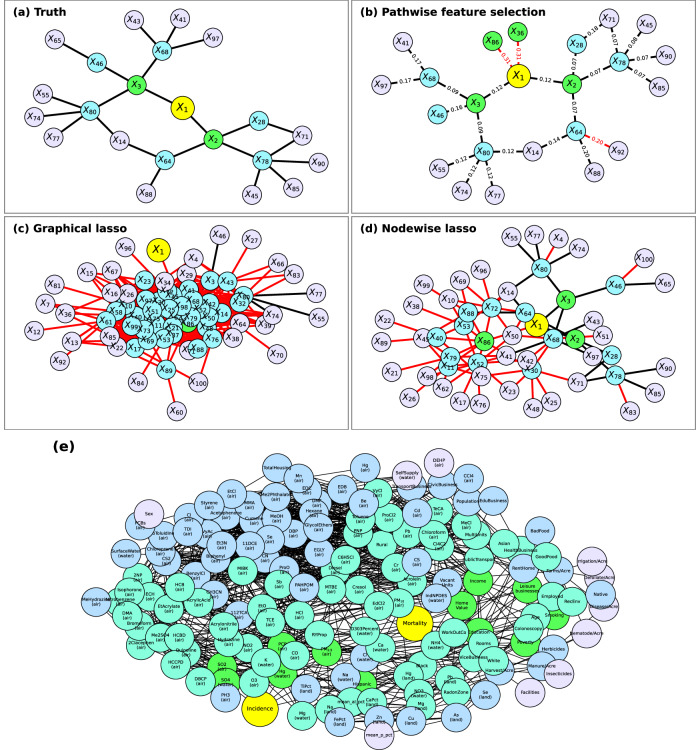
Benefits of pathwise false discovery control and limitations of existing methods on simulated and real data. **a** The true local graph of radius 3 around the target variable *X*_1_, shown in yellow. **b** Pathwise feature selection identifies 21 true edges and 3 false edges. It also provides edge-specific uncertainty quantification in the form of *q*-values---shown as edge weights---with lower *q*-values indicating greater confidence. (**c**) The graphical lasso^8^ identifies 12 true edges and 699 false edges despite the fact that the regularization parameter was tuned to yield the sparsest graph in which *X*_1_ has at least one neighbor (Section S2.1). **d** The nodewise lasso^9^ identifies 22 true edges and 57 false edges. All estimates are based on the same 200 samples drawn from a *p* = 100-dimensional Gaussian graphical model. True and false edges are black and red, respectively; green, blue, and purple nodes are distances 1, 2, and 3 from *X*_1_ in graphs (**a**)--(**d**). **e** Applying the graphical lasso to the environmental health dataset described in Section 2.4 yields a dense graph that obscures meaningful interactions.

Many CIG estimation methods fall into two broad classes. The first comprises regularized estimation approaches, most notably the graphical and nodewise lasso and their variants^8,9^. These methods scale to thousands of variables, but are prone to estimating dense graphs with many false positives (Fig. 1), often assume Gaussian data, and typically perform poorly when inter-variable relationships are nonlinear. The second class consists of methods based on explicit conditional independence testing, such as constraint-based approaches^10^. Previous work and results in this manuscript show that popular implementations of many methods in this class scale poorly with dimension^11–13^. As we will see, these approaches also tend to have relatively low statistical power, especially when the underlying graph contains nonlinear relationships or is moderately dense.

Crucially, while many methods are designed to estimate either the entire graph of inter-variable relationships (global estimation) or immediate neighborhoods of variables (Markov blanket estimation), far less work focuses on recovering extended local structures, such as paths or clusters, around target variables. Markov blanket estimates can in principle be combined to estimate such structures, and prior work has explored extending local procedures to produce global estimates^9,14^. However, theoretical results for these approaches often rely on idealized assumptions such as infinite samples^14^ or the ability to perfectly identify conditional independence relations from data^15^. In particular, they do not provide finite-sample error control at the level of extended local structures, which can lead to unreliable estimates in practice. More broadly, as we will show, global false discovery guarantees do not yield valid control of local errors.

To address these gaps, we propose *local graph estimation*, a statistical framework that formalizes the problem of recovering extended local structures around target variables, and introduce a method, *pathwise feature selection* (PFS), to solve it. Unlike global estimation methods, PFS avoids estimating irrelevant parts of the graph, enabling more accurate local inference around target variables while reducing computational burden in high dimensions. We also prove that PFS provides finite-sample false discovery control at the path level, establishing a highly interpretable framework for pathway discovery. In Fig. 1b, for example, our theory states that there is at most a 26% chance that the estimated path (*X*_1_, *X*_2_, *X*_78_, *X*_90_) is not in the true graph, which is the sum of the *q*-values—shown as edge weights—along that path.

We demonstrate local graph estimation and PFS on simulated data and in four diverse applications: an environmental and public health study of cancer; a multiomic breast cancer study; an analysis of brain networks and cognition; and a single-nucleus RNA sequencing study of Alzheimer’s disease. In simulations, PFS achieves a favorable balance between true positive and false discovery rates relative to existing approaches across a range of linear, nonlinear, sparse, and dense regimes. In all four applications, PFS yields interpretable local structure that is consistent with established domain knowledge. Together, these studies illustrate that local graph estimation and PFS are promising tools for uncovering meaningful, target-specific structure in complex high-dimensional data.

## Results

### Local graph estimation

A graph is a pair *G* = (*V*, *E*), where the set of nodes *V* = {1, …, *p*} indexes variables *X*_1_, …, *X*_*p*_, and *E* ⊆ *V* × *V* denotes the set of edges between pairs of nodes. The terms *variable* and *node* will be used interchangeably, since each variable maps to a unique node. In this work, we focus on *undirected graphs*, where edges (*j*, *k*) and (*k*, *j*) are identical, but local graph estimation can be considered for directed graphs as well. We assume *G* is a *conditional independence graph* for *X*, meaning that (*j*, *k*) is in *E* if and only if *j* ≠ *k* and *X*_*j*_ and *X*_*k*_ are conditionally dependent given all other variables; that is, there is an edge between *j* and *k* if and only if *X*_*j*_ and *X*_*k*_ contain information about each other that is not captured by the rest of the system. A unique such graph exists whenever the joint distribution of the variables is strictly positive^7^. Nodes *j* and *k* are called *neighbors* in *G* if (*j*, *k*) is in *E*; the *neighborhood* of a node is the set of all its neighbors. For example, the neighbors of *X*_3_ in Fig. 1a are *X*_1_, *X*_46_, *X*_68_, and *X*_80_.

Traditionally, graph estimation aims to infer all of *G* from *n* independent samples of a random vector *X* = (*X*_1_, …, *X*_*p*_), but this is often unnecessary when only a subset of *target variablesV*_0_ ⊆ *V* is of particular interest. In this case, let *B*_*r*_(*V*_0_) be the set of nodes that are reachable from any node in *V*_0_ by a path of length *r* or less (that is, for every node *j* in *B*_*r*_(*V*_0_), there is a sequence of at most *r* adjacent edges in *G* connecting *j* to a node in *V*_0_), and let *E*_*r*_(*V*_0_) be the set of edges (*j*, *k*) such that at least one of *j* or *k* is in *B*_*r*−1_(*V*_0_). The *local graph* of radius *r* around *V*_0_ is the subgraph *G*_*r*_(*V*_0_) = (*B*_*r*_(*V*_0_), *E*_*r*_(*V*_0_)) of *G* (see Fig. 1a for an illustration). Given a set of target variables *V*_0_ and a radius *r*, the goal of *local graph estimation* is to estimate *G*_*r*_(*V*_0_) from samples of *X*. Furthermore, we aim to do so in a way that controls false discoveries, applies to mixed variable types such as discrete and continuous data, and handles complex relationships such as nonlinear interactions. Additional details about the local graph estimation problem are in Section S1.

A natural approach to local graph estimation is to obtain an estimate $\widehat{G}$ of *G* using one of many existing full graph estimation methods, then estimate the local graph *G*_*r*_(*V*_0_) by the local graph of radius *r* around *V*_0_ in $\widehat{G}$. This is often implicitly done in practice; for example, whenever one draws conclusions about the neighborhood of a variable based on the full estimated graph $\widehat{G}$. However—in addition to being computationally expensive when *p* is large (Section S3.4)—results reported in Sections S5 and S10 show that this approach exhibits poor local graph estimation performance across a wide range of methods, including those with global false discovery rate control. In Section S2, we describe fundamental mathematical limitations of this approach that help explain the poor performance of full graph estimation methods in the context of local graph estimation.

### Pathwise feature selection

To address these limitations, we introduce *pathwise feature selection* (PFS), a method designed for local graph estimation. PFS constructs local graphs around target variables by iteratively applying feature selection and tracking uncertainty along paths in the estimated graph using *q*-values, which are formally defined in Section S3.1. Informally, each *q*-value *q*_*j*_(*k*) for *k* ≠ *j* is a number between 0 and 1 that quantifies uncertainty about whether the edge (*j*, *k*) is in *G*, with larger values indicating greater uncertainty. In the first iterative step of PFS, the neighborhood of each node *j* in *V*_0_ is estimated by including all nodes *k* such that *q*_*j*_(*k*) falls below a user-specified threshold. Let *S*_1_(*V*_0_) denote the set of newly selected nodes not already in *V*_0_. In the next step, each *j* in *S*_1_(*V*_0_) is treated as a response, and the same procedure is applied to estimate its neighbors, yielding a new layer *S*_2_(*V*_0_) of previously unidentified nodes, and so on. A full description of PFS is given in Algorithm 1 and discussed in the Methods section, with implementation details provided in Section S3.3.

In Section S3.2 we state and prove our main theoretical result, Theorem S3.2, which says that the sum of *q*-values along an estimated path upper bounds the probability that the path is not in the true graph *G*. This gives a principled stopping rule for PFS: the iterative procedure halts either when the maximum radius *r*_max_ is reached or when the cumulative uncertainty along a path—quantified by the sum of its *q*-values—exceeds a threshold ${q}_{\,{{{\rm{path}}}}}^{*}$. For example, in Fig. 1b, every node in the estimated local graph lies on a path starting at *X*_1_ whose *q*-values sum to at most ${q}_{\,{{{\rm{path}}}}}^{*}=0.4$. In particular, there is greater uncertainty in the false edges (1, 36) and (1, 86) (both with *q*-values of 0.31) than in the true edges (1, 2) and (1, 3). PFS therefore extends paths beyond *X*_1_ and *X*_2_, but not beyond *X*_36_ and *X*_86_ under this pathwise threshold. Further discussion of Theorem S3.2 is provided in the Methods section.

PFS offers several practical advantages beyond principled pathwise error control. Users can specify custom *q*-value thresholds for different nodes or node types in order to prioritize features of known importance or emphasize cross-modal connections. Adding to this flexibility, our implementation of PFS uses integrated path stability selection^16,17^ (IPSS) to estimate *q*-values nonparametrically, enabling detection of both linear and nonlinear relationships without requiring distributional assumptions such as Gaussianity (Methods). IPSS also employs a repeated subsampling procedure that has been shown to improve the robustness and reproducibility of feature selection^18^. Finally, in Table S1 we find that PFS with IPSS is significantly faster than other graph estimation methods in high dimensions, including local methods such as those based on Markov blanket estimation. A comparative study of PFS with IPSS and alternative *q*-value constructions is provided in Section S5.4

#### Algorithm 1

**(Pathwise feature selection)**

**Input**: Data matrix ${{{\boldsymbol{X}}}}\in {{\mathbb{R}}}^{n\times p}$, target features *V*_0_ ⊆ *V*, maximum radius ${r}_{\max }$, neighborhood FDR thresholds $\{{q}_{r}^{*}:1\le r\le {r}_{\max }\}$, path threshold ${q}_{{{{\rm{path}}}}}^{*}$, an algorithm for computing *q*-values

1: Initialize current features *S* ← *V*_0_, visited features *B* ← *V*_0_, and *q*-value matrix $Q\leftarrow {{{\bf{1}}}}\in {{\mathbb{R}}}^{p\times p}$

2: **For** *r* = 1 to ${r}_{\max }$ **do**

3:  **For** *j* ∈ *S* **do**

4:   Compute *q*-values *q*_*j*_(*k*) for all *k* ∈ *V*⧹{*j*}

5:   **If** $${q}_{j}(k)\le {q}_{r}^{*}$$ **then**

6:    $${Q}_{jk}\leftarrow {Q}_{kj}\leftarrow \min \{{q}_{j}(k),{Q}_{kj}\}$$

7:   **end if**

8:  **end for**

9:  Compute *d*_*Q*_(*V*_0_, *j*, *r*) for all *j* ∈ *V*⧹*B* (see Equation 1)

10: $$S\leftarrow \,\{j\in V\backslash B:{d}_{Q}({V}_{0},j,r)\le {q}_{{{{\rm{path}}}}}^{*}\}$$

11: *B* ← *B* ∪ *S*

12: **end for**

**Output**: Weighted adjacency matrix *Q* (see Section S3.3 for details)

### Simulation studies

We conducted simulation studies to evaluate local graph estimation performance when the true graph is known, varying sparsity, sample size, and whether relationships between the target variable and its neighbors were linear or nonlinear. We compared PFS to the graphical and nodewise lasso, five regularization-based methods with global false discovery rate (FDR) control, and seven constraint- or mutual information-based methods, six of which have both local and global variants. Descriptions and implementation details for each of these methods are in Section S4. Local graph recovery at varying radii around the target variable was assessed using true positive rate (TPR) and FDR (Equations S2.1 and S5.2). Full simulation settings and results are reported in Section S5.

Simulation results in Tables S2–S6 show that PFS achieves a favorable balance between TPR and FDR across all experiments. One reason for this is that PFS is the only method with rigorous pathwise false discovery control, which enables precise inference at target variables while preventing errors from propagating away from them. In linear settings, graphical and nodewise lasso recover many true edges but suffer from high FDRs, while several global FDR and constraint-based methods are overly conservative, yielding low FDRs at the cost of substantially reduced TPRs. PFS performs especially well in nonlinear settings, achieving the highest TPR among all methods at radii 1, 2, and 3 for both sparse and dense graphs. Furthermore, its consistent balance between discovery and error control across the dense settings indicates that PFS neither incurs excessive false negatives nor depends on sparsity for good performance. Finally, Fig. S2 shows the performance of PFS at different radii as *n* increases in the sparse, linear setting. With *p* fixed at 200, PFS maintains FDR control while TPR steadily increases, approaching one once *n* > *p*.

### Environmental and social drivers of cancer

Mounting evidence suggests that human health is shaped by interacting variables across multiple environmental, socioeconomic, and demographic domains^19–21^. With this in mind, we compiled county-level data from the Environmental Protection Agency (EPA), U.S. Census Bureau, National Cancer Institute (NCI), and the Centers for Disease Control and Prevention (CDC) to investigate cancer outcomes across the contiguous United States. After data cleaning (Section S6.2), the final dataset includes *p* = 165 variables measured across *n* = 2857 counties. Our two target variables are age-adjusted incidence and mortality rates for all cancers, obtained from the NCI and CDC as aggregate values spanning 2017–2022.

**Fig. 2:**
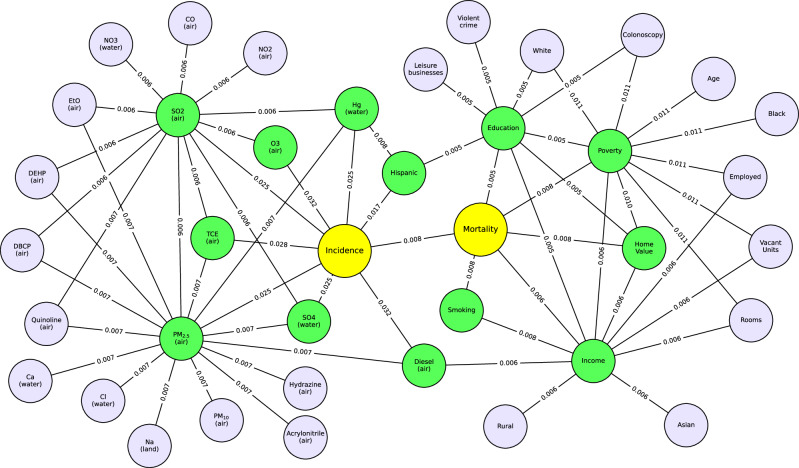
Radius 2 graph around county-level cancer incidence and mortality, estimated by PFS. The target variables, cancer incidence and mortality, are shown in yellow. Nodes directly connected to a target are shown in green, and nodes connected to a green node but not to either target are shown in purple. Edges are annotated with their *q*-values, with smaller values indicating stronger evidence of conditional dependence. Exposures are labeled by environmental medium (air, land, or water).

Figure 2 shows the local graph of radius 2 around cancer incidence and mortality estimated by PFS. Unlike other methods (Section S10.1), PFS reveals two distinct and largely non-overlapping sets of interacting variables. The first set, located in the left half of the graph, links incidence to environmental exposures. Several of these—notably fine particulate matter (PM_2.5_) and sulfur dioxide (SO_2_)—directly connect to compounds classified by the International Agency for Research on Cancer (IARC) as known or probable human carcinogens (Table S12). For example, PM_2.5_ links to SO_2_, which connects to other exposures such as ozone (O_3_), carbon monoxide (CO), and nitrogen dioxide (NO_2_), reflecting known atmospheric processes by which secondary pollutants are formed^22^.

**Fig. 3:**
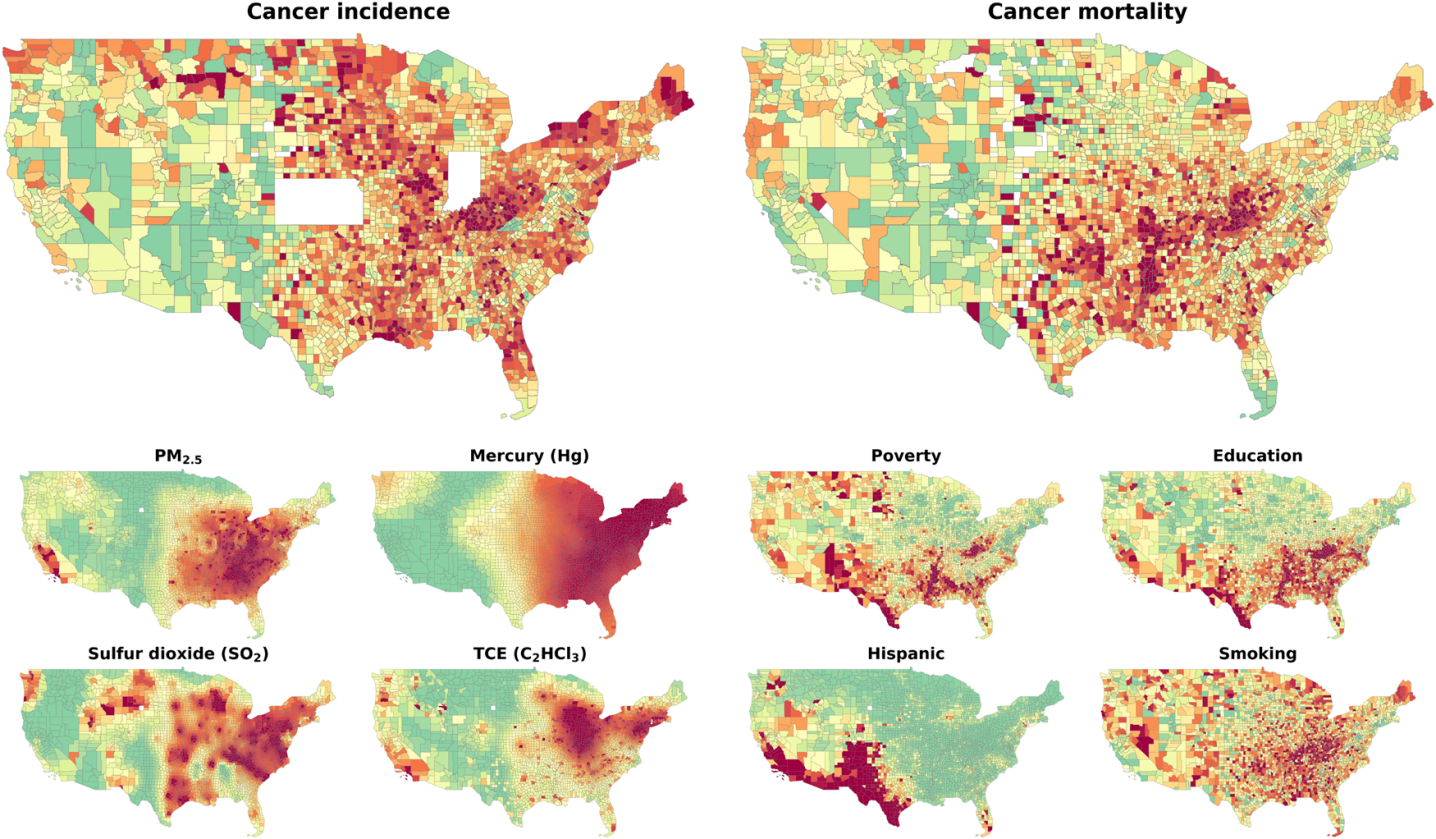
Geographic distributions of cancer burden and selected environmental and socioeconomic variables across the contiguous United States. Heatmaps show county-level values for cancer incidence and mortality (top row) and selected environmental exposures (bottom left) and social and demographic factors (bottom right). Cancer incidence tends to align with elevated levels of exposures---including particulate matter PM_2.5_, mercury, sulfur dioxide, and trichloroethylene (TCE)---while cancer mortality is more closely linked to socioeconomic conditions. Counties with large Hispanic populations, concentrated in the Southwest, show high poverty but low cancer burden, possibly due to reduced environmental exposures. In the Education map, red corresponds to lower education levels and green to higher levels; in all other maps, red and green indicate higher and lower levels of the variable, respectively. Indiana and Kansas did not report cancer incidence and are therefore omitted from the study.

The right half of the local graph, on the other hand, shows that cancer mortality connects most strongly to socioeconomic variables such as income and education. These conditional dependencies suggest a division of influence: even after adjusting for all other variables, environmental exposures remain strongly associated with cancer incidence, while survivorship is associated with social factors. This division is reflected in the heatmaps in Fig. 3. High-incidence counties cluster in regions with elevated environmental exposures, while high-mortality counties overlap with high poverty and low education areas, most notably in the Mississippi Delta and Appalachian regions of Kentucky and West Virginia. Despite stark differences in racial composition—the Mississippi Delta has among the highest proportions of black residents in the country, while the Appalachian counties are almost entirely white—both areas experience similarly high cancer mortality. This pattern aligns with the estimated local graph, where black and white are conditionally independent of mortality given poverty. Table S13 further supports this hypothesis, showing that the correlation between black and mortality drops from 0.228 (*p*-value  < 10^−10^) to 0.053 (*p*-value  = 0.005) after adjusting for poverty, while the correlation between poverty and mortality remains strong after adjusting for black (0.38, *p*-value  < 10^−10^). A similar asymmetry holds for white, reinforcing the role of poverty in shaping observed racial associations with mortality.

Counties in the Southwest with large Hispanic populations present a striking exception. These areas—spanning parts of Texas, New Mexico, Arizona, and Colorado—have poverty and education levels similar to those in the Mississippi Delta and Appalachia, yet exhibit significantly lower cancer incidence and mortality rates. One possible explanation supported by the local graph is that the Hispanic variable is associated with some of the lowest mercury levels in the country, which in turn links to low levels of other exposures. This raises the possibility that reduced exposure may lower cancer risk and contribute to lower mortality in these counties despite their socioeconomic disadvantage. Consistent with this interpretation, Table S13 shows that the correlation between Hispanic and incidence changes from  − 0.34 to  − 0.174 after adjusting for county-specific mercury levels. In contrast, adjusting for the four cancer screening variables only slightly reduces correlation from  − 0.34 to  − 0.324, indicating that low cancer incidence in counties with large Hispanic populations is unlikely to be explained by differences in screening rates.

### Cross-modal pathways in breast cancer

Multiomic studies integrate different types of data to better understand structure within biological systems and identify disease-relevant pathways^23^. In cancer research, this strategy has enabled the discovery of molecular signatures with diagnostic and prognostic value^24,25^. Here, we apply PFS to multiomic breast cancer data from The Cancer Genome Atlas^26^ (TCGA) to investigate three clinical variables: histological type (invasive ductal carcinoma, IDC, or invasive lobular carcinoma, ILC), pathologic stage (stages I–IV), and survival status at last follow-up. In addition to these three targets, the cleaned dataset (Section S7) includes *p* = 10, 741 variables measured across *n* = 547 breast cancer patients, namely: 9785 genes profiled by bulk RNA sequencing (RNA-seq), 819 microRNAs (miRNAs), and 137 proteins quantified by reverse phase protein arrays (RPPA). Because no ground-truth graph is available, we validate our findings through comparison with existing literature and additional statistical analyses, including over-representation analysis (ORA) and protein-protein edge validation (Section S7.4).

**Fig. 4:**
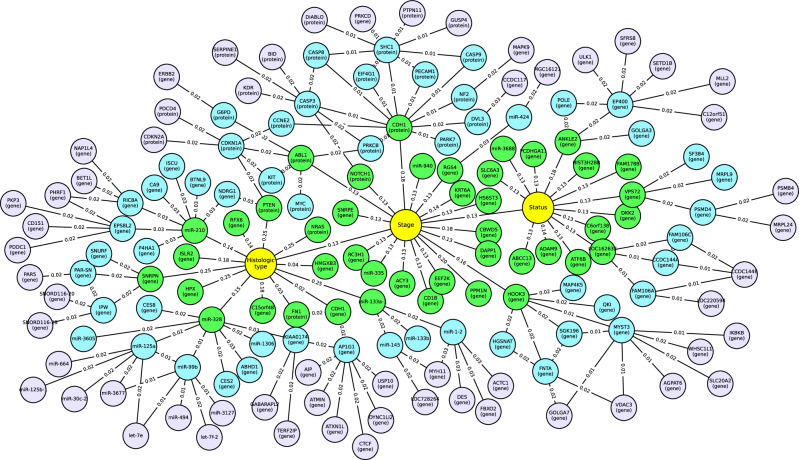
Radius 3 graph around clinical variables in TCGA breast cancer data, estimated by PFS. The target variables---histological type, pathologic stage, and survival status---are shown in yellow. Nodes directly connected to a target are shown in green, nodes at distance two are shown in blue, and nodes at distance three are shown in purple. Edges are annotated with *q*-values, with smaller values indicating stronger evidence of conditional dependence. Genes and proteins are labeled with “(gene)" and “(protein)" respectively, indicating their molecular modality.

The local graph estimated by PFS (Fig. 4) reveals a biologically coherent network linking molecular and clinical features across modalities. The three clinical variables form a path—histological type (henceforth, *subtype*) connects to pathologic stage, which connects to survival status—aligning with known clinical relationships. For example, ILC is often diagnosed at more advanced stages than IDC due to reduced detectability on mammography^27^, consistent with the edge between subtype and stage. Stage is a well-established predictor of survival, and the absence of an edge between subtype and survival aligns with clinical uncertainty about their prognostic relationship^28^.

CDH1 and FN1 have the strongest associations with subtype, forming a multimodal triangle with particularly small *q*-values. CDH1 encodes E-cadherin, whose inactivation is a defining feature of ILC, while FN1 has been linked to immune infiltration and poor prognosis in breast cancer^27,29,30^. ORA results reported in Table S14 indicate that the CDH1-associated gene cluster is significantly enriched for metastasis-related signatures. miR-210, which plays an established role in hypoxic response and metabolic adaptation, forms a compact module with the protein PTEN and known gene targets NDRG1 and ISCU^31,32^. ORA of this cluster shows enrichment for hypoxia-related gene sets, while ORA of the miR-133a-1 module implicates genes involved in muscle contraction and cytoskeletal organization, aligning with prior evidence that miRNAs in this group co-regulate and are frequently downregulated in breast cancer^33,34^.

Many edges emanating from pathologic stage are between proteins. Independent validation against the STRING database^35^ (Section S7.4) shows that PFS is the only method whose estimated graph exhibits significant enrichment for known protein-protein interactions (Table S15), supporting the biological plausibility of the protein subnetwork branching from stage. Stage is also part of a particularly notable pathway, connecting to ABL1, then to G6PD, and finally to the canonical oncogene ERBB2 (HER2). ABL1 is a non-receptor tyrosine kinase involved in oncogenic signaling, while G6PD plays a key role in oxidative stress regulation in HER2-positive breast cancer^36^.

ORAs of two gene clusters linked to survival, namely those emanating from ANKLE2 and HOOK3, show strong enrichment for breast cancer-specific copy number and mutation signatures (Table S14). In both cases, the enriched gene sets are derived from recurrently amplified or mutated genomic regions in breast cancer, providing strong evidence that the survival-associated structure recovered by PFS reflects established disease biology rather than isolated gene-level associations.

Despite the validation results presented above, not all edges or clusters of edges in the estimated local graph admit a straightforward functional interpretation, and we do not attempt to assign biological roles to every substructure within it. Instead, we emphasize that PFS offers a structured and statistically grounded view of potential dependencies—both within and between modalities—that may warrant further investigation. In this way, local graph estimation can serve not only to recover known biology but to generate new hypotheses for future experimental validation.

### Brain networks and cognition

The Human Connectome Project (HCP) is a large-scale neuroimaging effort to map macroscopic brain circuits and study their relationship to human behavior^37^. We applied PFS to data from the HCP Young Adult cohort to investigate brain network organization and cognitive ability. After preprocessing (Section S8.2), the dataset includes *n* = 1188 individuals and *p* = 213 variables comprising various structural brain measurements—including cortical thickness, surface area, and regional volumetric measures across Desikan-Killiany regions^38^—as well as behavioral and personality phenotypes. The target variable is the age-adjusted Fluid Cognition Composite score from the NIH Toolbox (henceforth, *fluid cognition*), a standardized measure summarizing executive function, working memory, and processing speed.

Figure S4 shows the local graph of radius 3 around fluid cognition estimated by PFS. Estimates from other methods are shown in Fig. S21. ORA results in Table 1 indicate that at radius 2, both PFS and a local version of the hybrid parents and children algorithm^39^, HPC(L), are enriched for regions in the frontoparietal control network (FPCN). At radius 3, both methods remain enriched for FPCN, while only PFS—whose graph contains 23 nodes at this radius—shows additional enrichment for the ventral attention network (VAN), suggesting greater specificity than HPC(L) (36 nodes).

**Table 1:** Enrichment of local brain networks associated with fluid cognition

Method	Radius	Nodes	VIS	SM	DAN	VAN	LIM	FPCN	DMN
**PFS**	2	7	–	–	–	–	–	0.0027	–
	3	23	–	–	–	0.0655	–	0.0109	–
HPC(L)	2	8	–	–	–	–	–	0.0042	–
	3	36	–	–	–	–	–	0.0569	–
IAMB(L)	2	14	< 10^−4^	–	–	–	–	–	–
	3	100	0.0218	–	–	–	–	–	–
MMPC(L)	2	6	0.0027	–	–	–	–	–	–
	3	24	0.0009	–	–	–	–	–	–
StablePC	2	5	< 10^−4^	–	–	–	–	–	–
	3	12	< 10^−4^	–	–	–	–	–	–
GFCSL	2	18	–	–	0.0342	–	–	–	–
	3	148	–	–	–	–	–	–	–
Glasso	2	10	< 10^−4^	–	–	–	–	–	–
	3	106	0.0971	–	–	–	–	–	–

For each method, we report *p*-values from over-representation analyses for Yeo-7 functional networks^48^ among cortical regions in the estimated local graph. Local clusters are anchored at a single region of interest that connects directly to the target; radii correspond to cumulative neighborhoods containing all nodes within graph distances 2 and 3 of the target that extend beyond this anchor. The “Nodes" column reports the total number of nodes in each cluster. The Yeo-7 networks are: VIS (visual), SM (somatomotor), DAN (dorsal attention), VAN (ventral attention), LIM (limbic), FPCN (frontoparietal control), and DMN (default mode). Only *p*-values less than 0.1 are shown; entries marked “–" indicate no enrichment at this threshold.

The prominence of FPCN in the local graphs estimated by PFS and HPC(L) is consistent with prior work showing that this network plays a central role in executive control and flexibly couples with other large-scale systems depending on task demands^40,41^. In contrast to other methods (Table 1), neither PFS nor HPC(L) exhibits enrichment for visual (VIS) or somatomotor (SM) networks at comparable radii. VIS and SM are *unimodal* regions that primarily support sensory processing and motor function rather than integration across cortical systems^42^. The absence of VIS/SM enrichment, together with FPCN (and, for PFS, VAN) involvement, aligns with fluid cognition, which emphasizes working memory, processing speed, and cognitive control.

### Cell-type-specific gene networks in Alzheimer’s disease

Single-nucleus RNA sequencing (snRNA-seq) enables profiling of gene expression in individual cells. Grubman et al.^43^ generated snRNA-seq data from the entorhinal cortex of six individuals with Alzheimer’s disease (AD) and six controls to characterize transcriptional changes associated with the disease. We applied PFS to these data to identify AD-associated gene networks in three cell types: astrocytes (*n* = 2171 cells, *p* = 10, 788 genes), microglia (*n* = 449, *p* = 10, 514), and oligodendrocyte progenitor cells (OPCs; *n* = 1078, *p* = 10, 773). The target variable is AD status, defined as whether a cell is from an AD or control individual. Additional dataset details are in Section S9.

Figure S5 to S7 show the local graphs of radius 3 around AD status estimated by PFS. ORAs of these graphs (Table 2) indicate distinct biological mechanisms for each cell type. Specifically, the microglial graph is enriched for immune activation and inflammatory signaling, consistent with the central role of neuroinflammation in AD. The astrocyte graph shows enrichment for mitochondrial respiration and ion transport, reflecting altered energy metabolism and oxidative stress in diseased tissue. The OPC graph highlights central nervous system development, cell adhesion, and synapse-related processes. Several genes with well-established roles in AD, such as APOE and GFAP, appear in the estimated graphs. Importantly, the three analyses yield different local structures despite sharing the same target, suggesting that PFS captures cell-type-specific disease organization rather than a generic AD signature.

**Table 2:** Enrichment of PFS local graphs around AD status by cell type

Cell Type	Enriched biological process	Adjusted *p*-value
**Astrocytes**		
	GO:BP: Monoatomic ion transport	0.001
	GO:BP: Oxidative phosphorylation	0.001
	GO:BP: ATP synthesis coupled electron transport	0.002
	Reactome: Aerobic respiration and respiratory electron transport	0.012
	Reactome: Respiratory electron transport	0.012
	Reactome: Extracellular matrix organization	0.031
**Microglia**		
	GO:BP: Regulation of cell activation	< 10^−3^
	GO:BP: Positive regulation of leukocyte proliferation	< 10^−3^
	GO:BP: Regulation of lymphocyte activation	< 10^−3^
	GO:BP: Adaptive immune response	< 10^−3^
	Reactome: Cytokine signaling in immune system	0.024
	Reactome: Hemostasis	0.029
**Oligodendrocyte progenitor cells (OPCs)**		
	GO:BP: Central nervous system development	< 10^−3^
	GO:BP: Cell-cell adhesion	< 10^−3^
	GO:BP: Presynapse assembly	< 10^−3^
	GO:BP: Synapse organization	< 10^−3^
	Reactome: Protein–protein interactions at synapses	0.012
	Reactome: Neuronal system	0.013

Over-representation analyses were performed using Reactome and Gene Ontology Biological Process (GO:BP) gene sets (Section S9.4). Background sets consist of all genes analyzed by PFS. All reported terms have an overlap of at least 5 genes.

## Discussion

We introduced local graph estimation, a statistical objective focused on identifying substructures around target variables in complex data, and proved that our proposed solution, PFS, provides both edgewise and pathwise uncertainty quantification. Together, local graph estimation and PFS enable statistically principled discovery of paths and clusters of variables, opening new directions for inference on localized network structure. Local graph estimation also supports downstream analyses that rely on graph structure, including graph-based clustering approaches^44^.

PFS is broadly applicable. When implemented with nonparametric selection methods such as IPSS, it accommodates mixed data types, avoids strong modeling assumptions, and readily scales to tens of thousands of variables. Across four distinct applications, we showed that PFS recovers established domain structure while revealing potentially novel relationships. More broadly, local graph estimation offers a flexible and interpretable alternative to global graph estimation for identifying candidate mechanistic relationships centered on variables of primary scientific interest.

## Methods

### Description of algorithm 1

Algorithm 1 provides a step-by-step description of PFS. The algorithm takes as input an *n* × *p* data matrix ***X*** comprising *n* observations of *p* variables, a set of target features *V*_0_, and user-specified *q*-value thresholds. The output is a weighted *p* × *p* adjacency matrix, *Q*, whose entries are the pairwise *q*-values computed during the iterative selection process. Neighborhood thresholds ${q}_{r}^{*}$ control the false discovery rate (FDR) within each estimated neighborhood at iteration *r*, while the path threshold ${q}_{{{{\rm{path}}}}}^{*}$ constrains the maximum sum of *q*-values along any path, thereby providing an upper bound on the probability that a recovered path does not belong to the true graph under the assumptions of Theorem S3.2.

The quantity *d*_*Q*_(*V*_0_, *j*, *r*) in Line 9 is the minimum sum of *q*-values along any path of length *r* from *V*_0_ to node *j* in the estimated graph, namely

1 $$\begin{array}{r}{d}_{Q}({V}_{0},j,r)=\min \left\{{\sum }_{s=0}^{r-1}{Q}_{{j}_{s},{j}_{s+1}}:({j}_{0},\ldots,{j}_{r})\in {\widehat{{{{\mathcal{J}}}}}}_{r}({V}_{0}),\,{j}_{r}=j\right\},\end{array}$$

where ${\widehat{{{{\mathcal{J}}}}}}_{r}({V}_{0})$ is the set of length-*r* paths in the current estimated local graph that start in *V*_0_ (see Sections S1 and S3 for a complete discussion of definitions and notation). Line 10 updates the new layer *S* of previously unidentified nodes, and Line 11 updates the set *B* of nodes whose neighborhoods have already been estimated. Together, these updates guarantee that each neighborhood is estimated at most once during the recursive process.

### Discussion of theorem S3.2

Theorem S3.2 upper bounds the probability that a given path does not belong to the true graph by the sum of the edge-level *q*-values along that path. This result applies to individual paths, but does not provide joint or simultaneous control over collections of paths; in particular, paths that share edges are dependent, complicating extensions of Theorem S3.2 to such settings. The assumptions of Theorem S3.2 are a direct extension of classical assumptions used to justify *q*-values in multiple testing (Section S3.1). Specifically, Theorem S3.2 supposes that, along a given path, test statistics used to determine whether each edge is selected are independent. Informally, this means that whether one edge appears in an estimated path depends only on the evidence for that edge, and not on the evidence for other edges along the path. If these assumptions are violated—for example, if strong dependence between edge-level test statistics causes the selection of one edge to influence the apparent evidence for another—then error control may fail. When these assumptions do hold, edge-level *q*-values can be interpreted as posterior probabilities that a selected edge is not in the true graph^45^. Extending this to paths gives Theorem S3.2 a natural Bayesian interpretation: the sum of *q*-values along a path upper bounds the posterior probability that an estimated path is not in the true graph (T S3.2), motivating our use of *q*-values to limit uncertainty propagation as paths extend away from targets.

### Integrated path stability selection

At each iteration of PFS, we estimate edges using integrated path stability selection (IPSS)^16^, a feature selection method that provides finite-sample false discovery control. IPSS is a refinement of classical stability selection^46^, in which an arbitrary base feature selection algorithm is repeatedly applied to random half-samples of the data. The proportion of times each feature is selected across all subsamples is computed and then aggregated across regularization levels (for example, penalty parameters or importance score thresholds) to produce feature-specific *stability paths*. Rather than applying a hard threshold to stability paths as in classical stability selection, IPSS integrates information along stability paths to yield more precise *q*-values and tighter false discovery control than previous approaches. IPSS also retains the favorable robustness properties of stability selection, which arise from the repeated subsampling scheme. In this work, we use the nonparametric variant of IPSS, which is compatible with arbitrary variable importance measures and captures nonlinear associations without requiring distributional or parametric modeling assumptions^17^. Specifically, we implement IPSS using mean decrease impurity from random forests and gradient boosting, which is computationally efficient and the default choice in many popular machine learning packages.

### PFS parameters

The principal PFS parameters are the maximum radius ${r}_{\max }$ of the estimated local graph, the pathwise *q*-value threshold, and, optionally, edge-level *q*-value thresholds. There is no single “correct” choice of these parameters in general: *q*-value thresholds encode how much uncertainty a user is willing to tolerate in the estimated graph, which is inherently problem-specific. As with significance levels in hypothesis testing more broadly, there is no universally optimal choice of edgewise or pathwise *q*-value thresholds, nor an oracle rule for selecting them. The choice of ${r}_{\max }$ reflects the scope of the scientific question rather than uncertainty tolerance. In some applications, domain knowledge may suggest that relationships beyond a certain distance from the target variables are not of interest, in which case ${r}_{\max }$ can be fixed accordingly. Alternatively, ${r}_{\max }$ can be made arbitrarily large, leaving the breadth of the local graph to be determined entirely by the pathwise *q*-value threshold. Larger radii and more liberal thresholds will generally yield broader, denser local graphs with more discoveries but greater uncertainty, while smaller radii and stricter thresholds will produce sparser, potentially more interpretable, structures with less uncertainty. PFS is designed to make these tradeoffs explicit and user-controlled, rather than implicit or fixed by the method.

### Simulation design for Fig. 1

The data used in Fig. 1, panels (a) through (d), are generated from a *p* = 100 dimensional multivariate Gaussian distribution with mean zero and precision matrix *Θ*, denoted ${{{\mathcal{N}}}}(0,{\Theta }^{-1})$. The precision matrix is composed of three blocks of sizes 1 × 1, 2 × 2, and 97 × 97, corresponding to the target variable, its neighbors, and all remaining variables. Within-block and between-block connections are generated at random such that the average degree of the graph is approximately 3, and nonzero entries of *Θ* are chosen uniformly at random from { ± 1}. The resulting matrix is symmetrized, a positive constant is added to the diagonal to ensure positive definiteness, and the matrix is rescaled so that its eigenvalues lie between 0.01 and 10, allowing for strong correlations between variables. Given *Θ*, we draw *n* = 200 samples independently from ${{{\mathcal{N}}}}(0,{\Theta }^{-1})$, and standardize the resulting data to have zero mean and unit variance.

## Supplementary information

Supplementary information

Transparent Peer Review file

## Data Availability

All data used in this work are publicly available. For the environmental and sociodemographic cancer study, county-level data on cancer incidence, mortality, screening, and smoking prevalence are available from the State Cancer Profiles project at https://statecancerprofiles.cancer.gov/. Environmental and socioeconomic variables are available from the EPA Environmental Quality Index (EQI) at https://cfpub.epa.gov/ncea/risk/recordisplay.cfm?deid=316550, and demographic data are available from the U.S. Census Bureau at https://data.census.gov/. Data from the multiomic breast cancer study can be downloaded from LinkedOmics at https://www.linkedomics.org/data_download/TCGA-BRCA/. Data from the brain network and cognition analysis were obtained from the Human Connectome Project (HCP Young Adult cohort) via ConnectomeDB (https://www.humanconnectome.org), accessed through the BALSA data portal at https://balsa.wustl.edu. Data from the Alzheimer’s disease study can be downloaded from https://adsn.ddnetbio.comand are also available from the Gene Expression Omnibus (GEO) under the accession number GSE138852.

## Code availability

Code and processed data files required to reproduce all results in this paper are available at https://github.com/omelikechi/localgraph-paperand archived on Zenodo^47^. A Python package implementing local graph estimation and PFS is available at https://github.com/omelikechi/localgraphand can be installed via PyPI at https://pypi.org/project/localgraph/.
