# Supplementary material for: Local graph estimation with pathwise false discovery control
Source: Nat Commun. 2026 May 12;17:6353. doi: 10.1038/s41467-026-72796-9 (PMC13376902; doi:10.1038/s41467-026-72796-9)
Supplement: Supplementary file 1 — Supplementary information [file 41467_2026_72796_MOESM1_ESM.pdf]

Supplementary information for "Local graph estimation with pathwise false discovery control"

Omar Melikechi[1], David B. Dunson[1], Noureddine Melikechi[2], and Jeffrey W. Miller[3]

S1. Local graph estimation

In this section, we formally define the local graph estimation problem. Let $X = (X_1, \ldots, X_p)$ be a random vector with conditional independence graph $G = (V, E)$; as noted in Section 2.1, a unique such G exists whenever the distribution of X is positive[1]. A *path* of length r is a sequence of distinct nodes $\boldsymbol{j} = (j_0, \ldots, j_r) \in V^{r+1}$. The path $\boldsymbol{j}$ lies in G if $(j_s, j_{s+1}) \in E$ for all $0 \leq s < r$, and it starts in $V_0 \subseteq V$ if $j_0 \in V_0$. The *distance* $d(j,k)$ in G between $j, k \in V$ is the length of the shortest path in G from j to k. If no path exists, then $d(j,k) = \infty$. For $V_0 \subseteq V$, define $B_0(V_0) = V_0$ and, for $r \in \mathbb{N}$,

$$B_r(V_0) = \big\{k \in V : \min_{j \in V_0} d(j,k) \leq r\big\}. \tag{S1.1}$$

$B_r(V_0)$ is the ball of radius r around V_0. Its boundary, $S_r(V_0) = B_r(V_0) \setminus B_{r-1}(V_0)$, is the set of nodes that are distance r from V_0 (the notation S stands for *sphere*, complementing the ball notation, B). For $r \in \mathbb{N}$, define the edge set

$$E_r(V_0) = \big\{(j,k) \in E : j \in B_{r-1}(V_0) \text{ or } k \in B_{r-1}(V_0)\big\}. \tag{S1.2}$$

Edges between nodes in the outer layer $S_r(V_0)$ are omitted in our definition of $E_r(V_0)$ to avoid unnecessary estimation when performing pathwise feature selection (PFS). This exclusion does not compromise the conditional independence structure under the assumption that the distribution of X is positive; in particular, if X has a positive distribution, then $B_{r-1}(V_0)$ is conditionally independent of $V \setminus B_r(V_0)$ given $S_r(V_0)$; see, for example, Corollary 4.1 in Koller and Friedman[1].

The *local graph* of radius r around V_0 is the subgraph $G_r(V_0) = (B_r(V_0), E_r(V_0))$ of G. When $V_0 = \{j\}$ consists of a single node j, we write $G_r(j)$ as a shorthand for $G_r(\{j\})$, and $S_1(j)$ for the neighborhood of j. In the above notation, the local graph estimation problem is: *Given $V_0 \subseteq V$ and a radius $r \in \mathbb{N}$, estimate $G_r(V_0)$ from samples of X.* The two extremes, namely estimating $G_1(V_0)$ when V_0 contains a single variable (often referred to as supervised feature selection or Markov blanket estimation), and estimating the full graph, $G_\infty(V_0) = G$ (traditional graph estimation) are widely studied; the intermediate cases are not.

S2. Limitations of full graph estimation

In Section 2.1, we remarked that one way to perform local graph estimation is to obtain an estimate $\widehat{G}$ of the full graph G, then estimate $G_r(V_0)$ by $\widehat{G}_r(V_0) = (\widehat{B}_r(V_0), \widehat{E}_r(V_0))$, where $\widehat{B}_r(V_0)$ and $\widehat{E}_r(V_0)$ are defined as in Equations S1.1 and S1.2 but with $\widehat{G}$ in place of G. In this section, we describe

[1]Department of Statistical Science, Duke University, Durham, NC, USA
[2]Kennedy College of Sciences, University of Massachusetts Lowell, Lowell, MA, USA
[3]Department of Biostatistics, Harvard T.H. Chan School of Public Health, Boston, MA, USA

some key limitations of this general approach, highlighting the need for methods like PFS that are specifically tailored to the local estimation problem.

S2.1. Limitations of global methods. Most full graph estimation methods fall into one of two categories: *Global* methods estimate the full graph simultaneously, while *nodewise* methods estimate the neighborhood of each node, then combine these estimates to infer the full graph. Perhaps the most popular global method is the graphical lasso[2], which assumes that $X \sim \mathcal{N}(0, \Theta^{-1})$ is multivariate Gaussian with precision matrix Θ. In this setting, X_j and X_k are conditionally dependent given all other variables, denoted $X_j \perp\!\!\!\perp X_k \mid X_{V\setminus\{j,k\}}$, if and only if $\Theta_{jk} = 0$, so estimating G is equivalent to estimating the set of zero entries of Θ. The graphical lasso estimates Θ via

$$\widehat{\Theta}(\lambda) = \arg\min_{\Theta \succ 0} \mathtt{tr}(\widehat{\Sigma}\Theta) - \log\det\Theta + \lambda\|\Theta\|_1,$$

where $\widehat{\Sigma}$ denotes the empirical covariance matrix, $\|\Theta\|_1 = \sum_{i,j=1}^{p}|\Theta_{ij}|$ is the ℓ_1-norm of Θ, and the minimization is over all symmetric positive-definite matrices $\Theta \succ 0$. The regularization parameter $\lambda \geq 0$ encourages sparse solutions, with larger values of λ typically yielding more zeros in $\widehat{\Theta}(\lambda)$.

A key limitation of the graphical lasso with respect to local graph estimation is that it is often impossible to choose a λ that accurately estimates subgraphs of the true graph. Specifically, under the Gaussian assumption, a necessary condition[3] for an edge to exist between j and any other node is $\lambda < \max\{|\widehat{\Sigma}_{jk}| : k \in V \setminus \{j\}\}$. Figure 1c shows the estimate $\widehat{G}_3(1)$ of the local graph of radius 3 around X_1 obtained using the graphical lasso with $\lambda = 0.9999 \max\{|\widehat{\Sigma}_{1k}| : k \neq 1\}$ applied to data simulated from a 100-dimensional Gaussian distribution, as described in the Methods section. Despite being essentially the sparsest estimate that includes the edge $(1, 2)$, this choice of λ yields 698 false edges within radius 3 of X_1. As shown in Section S5, such spurious edges are typical of the graphical lasso, particularly in scenarios where the connections between a target variable and its neighbors are weaker than the connections between those neighbors and other variables.

Global inference is possible for Gaussian graphical models due to the exceptional correspondence between the graph G and the sparsity pattern of Θ in this setting. To relax the Gaussian assumption, many other methods for undirected graph estimation transform the data and then apply the graphical lasso[4,5]. However, since these methods ultimately rely on the graphical lasso in the final step, they are subject to the same limitation described above, regardless of the initial transformation.

S2.2. Limitations of global error control. Much recent work in graphical modeling focuses on developing methods for full graph estimation with *false discovery rate* (FDR) control[6–8]. The FDR of an estimator $\widehat{E}$ of the edge set E is the expected value of the *false discovery proportion* (FDP),

$$\mathrm{FDP}(\widehat{E}) = \frac{|\widehat{E} \setminus E|}{\max\{|\widehat{E}|, 1\}},$$

where $|A|$ is the size of a set A and the maximum in the denominator is taken to avoid division by zero. For $V_0 \subseteq V$ and a radius $r \in \mathbb{N}$, the analogue for the local graph of radius r around V_0 is

$$\mathrm{FDP}(\widehat{E}_r(V_0)) = \frac{|\widehat{E}_r(V_0) \setminus E_r(V_0)|}{\max\{|\widehat{E}_r(V_0)|, 1\}}. \tag{S2.1}$$

Figure S1 shows that the global FDP of the full estimated graph can be very different from the FDPs within local graphs around specific nodes. For example, in the left panel of Figure S1 we have $\mathrm{FDP}(\widehat{E}) = 0.1$ and $\mathrm{FDP}(\widehat{E}_1(3)) = 1$, while in the right panel we have $\mathrm{FDP}(\widehat{E}) = 0.9$ and $\mathrm{FDP}(\widehat{E}_1(1)) = 0$. Thus, methods with global FDR control do not guarantee error control within

local graphs. This is critical from the point of view of interpretability: We cannot conclude that the local graph around a variable of interest is accurate just because the global error rate is low, nor can we conclude that a local graph is inaccurate just because the global error rate is high.

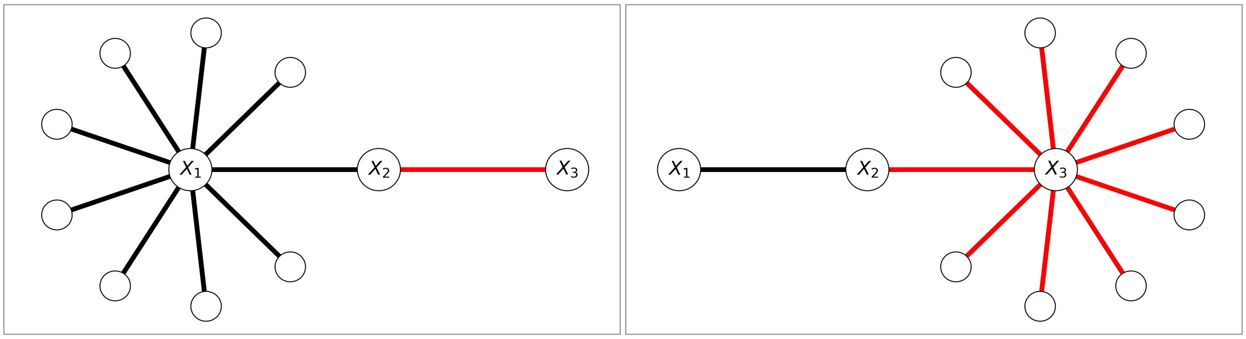

FIGURE S1. *Global error ≠ local error.* Hypothetical estimated graphs, with black and red edges representing true and false positives, respectively. In both cases, we have $\mathrm{FDP}(\widehat{E}_1(1)) = 0$, $\mathrm{FDP}(\widehat{E}_1(2)) = 0.5$, and $\mathrm{FDP}(\widehat{E}_1(3)) = 1$. (*Left*) The false discovery proportion of the full estimated graph is $\mathrm{FDP}(\widehat{E}) = 0.1$. (*Right*) The false discovery proportion of the full estimated graph is $\mathrm{FDP}(\widehat{E}) = 0.9$.

The practical implications of the difference between global and local error control are evident in Section S10, where multiple global FDR methods are applied to the county-level cancer and breast cancer datasets. In Figure S18, for example, Gaussian graphical modeling with FDR control using scaled lasso[6] (GFCSL), implemented in the R package `SILGGM`[7], yields an extremely dense local subgraph of radius 2 around the three clinical targets (Figure S18c). This occurs even though the radius-1 subgraph is reasonably sparse and the target global FDR was set to 0.1, which is the default value in `SILGGM` and the smallest threshold at which an edge is placed between pathologic stage and status (Figure S18a). Taken together, these results illustrate that global FDR guarantees often fail to both recover the immediate neighborhoods of target variables *and* constrain the number of edges at larger radii, significantly limiting their ability to yield interpretable local structure.

S3. Pathwise feature selection: Theory and implementation

Recall the description of pathwise feature selection (PFS) in Section 2.1: for each variable of interest $j_0 \in V_0$, perform feature selection to obtain an estimate $\widehat{S}_1(j_0)$ of the neighborhood $S_1(j_0)$ of j_0. Then, for each $j_0 \in V_0$ and each $j_1 \in \widehat{B}_1(j_0) \setminus V_0$, perform feature selection again to obtain an estimate $\widehat{S}_1(j_1)$ of $S_1(j_1)$, and so on, until some stopping criterion is met.

As described, the above procedure is a generic nodewise selection algorithm that begins in V_0 and terminates according to some criterion. Similar methods for full graph estimation already exist[7,9]. The critical difference is that PFS quantifies the accumulation of uncertainty along estimated paths that start in V_0, enabling more precise inference near the target variables. To do this, we extend the concept of q-values to paths. First, we recall the usual definition of a q-value.

S3.1. Storey's q-values. Graph estimation can be framed as a multiple hypothesis testing problem in which hypotheses $H_j(k) = \mathbb{1}(k \in S_1(j))$ are tested for all pairs $j, k \in V$. That is, $H_j(k) = 0$ if $(j,k) \notin E$ and $H_j(k) = 1$ otherwise. For each $j \in V$ and $k \in V \setminus \{j\}$, suppose the test $H_j(k)$ is based

on a statistic $T_j(k)$. We assume for simplicity that the null hypothesis $H_j(k) = 0$ is rejected at level α if and only if $T_j(k) \leq \alpha$, though the following results hold for more general significance regions[10].

Fix $j \in V$ and let $\widehat{S}_1(j, \alpha) = \{k : T_j(k) \leq \alpha\}$ be the set of nodes whose null hypotheses $H_j(k)$ are rejected at level α. As the notation suggests, $\widehat{S}_1(j, \alpha)$ is an estimator of $S_1(j)$. For $k \in V$ and an observed value $t_j(k)$ of the random test statistic $T_j(k)$, the associated *q-value* is

$$q_j(k) = \inf_{\{\alpha : t_j(k) \leq \alpha\}} \mathrm{pFDR}(\alpha) = \inf_{\{\alpha : t_j(k) \leq \alpha\}} \mathbb{E}\left(\frac{\mathrm{FP}_j(\alpha)}{|\widehat{S}_1(j, \alpha)|} \;\middle|\; |\widehat{S}_1(j, \alpha)| > 0 \right), \tag{S3.1}$$

where $\mathrm{FP}_j(\alpha) = |\widehat{S}_1(j, \alpha) \setminus S_1(j)|$ is the number of false positives in $\widehat{S}_1(j, \alpha)$, the second equality is the definition of the *positive false discovery rate*, pFDR, and $\mathbb{E}(A \mid B)$ is the conditional expectation of A given B. Intuitively, $q_j(k)$ is the smallest expected proportion of incorrectly rejected hypotheses among all rejected hypotheses, taken over all thresholds α for which the null hypothesis $H_j(k)$ is rejected. The following theorem shows that q-values admit a Bayesian interpretation.

Theorem S3.1. *Fix $j \in V$ and suppose that hypothesis tests $H_j(k)$ are performed with the test statistics $T_j(k)$ for all $k \in V \setminus \{j\}$. Assume that the $(T_j(k), H_j(k))$ are independent and identically distributed (iid) with $T_j(k) \mid H_j(k) \sim (1 - H_j(k))F_{0j} + H_j(k)F_{1j}$ for some null distribution F_{0j} and alternative distribution F_{1j}, and that $H_j(k) \sim \mathrm{Bernoulli}(\pi_1)$ are iid Bernoulli random variables for all $k \in V \setminus \{j\}$. Then for an observation $t_j(k)$ of $T_j(k)$,*

$$q_j(k) = \inf_{\{\alpha : t_j(k) \leq \alpha\}} \mathbb{P}\big(H_j(k) = 0 \mid T_j(k) \leq \alpha\big). \tag{S3.2}$$

Theorem S3.1 combines Theorem 1 and Corollary 2 from John Storey's seminal work on q-values[10]. This can be given a Bayesian interpretation in which the hypotheses $H_j(k)$ are given iid Bernoulli(π_1) priors, and the statistics $T_j(k) \mid H_j(k)$ are modeled as iid draws from the mixture distribution $(1 - H_j(k))F_{0j} + H_j(k)F_{1j}$. Under this setup, $\mathbb{P}(H_j(k) = 0 \mid T_j(k) \leq \alpha)$ represents the posterior probability that a Type I error was made, given that the null was rejected when using a threshold of α. Thus, the q-value $q_j(k)$ is the smallest posterior probability of a Type I error over all thresholds α greater than the observed test statistic $t_j(k)$. Equivalently,

$$q_j(k) = \inf_{\{\alpha : t_j(k) \leq \alpha\}} \mathbb{P}\big(k \notin S_1(j) \mid k \in \widehat{S}_1(j, \alpha)\big) \tag{S3.3}$$

is the smallest posterior probability that (j, k) is a false positive, given that k is in the estimated neighborhood $\widehat{S}_1(j, \alpha)$ of node j.

S3.2. Generalization to paths. Storey's q-values quantify uncertainty in individual edge estimates. In this section, we extend the concepts from Section S3.1 to quantify uncertainty in estimated paths. Let $V_0 \subseteq V$ be a subset of variables of interest in the true graph $G = (V, E)$, and let $H_j(k)$ and $T_j(k)$ be hypotheses and test statistics, respectively, as defined above.

For $\alpha \in \mathbb{R}^p$, recursively define $\widehat{B}_0(V_0, \alpha) = V_0$ and, for each integer $s \geq 0$,

$$\widehat{B}_{s+1}(V_0, \alpha) = \bigcup_{j \in \widehat{B}_s(V_0, \alpha)} \widehat{B}_1(j, \alpha_j),$$

where $\widehat{B}_1(j, \alpha_j) = \{j\} \cup \{k : T_j(k) \leq \alpha_j\}$ is the estimated ball of radius 1 around j. $\widehat{B}_r(V_0, \alpha)$ consists of all nodes that are estimated to be within distance r of V_0 under the significance thresholds

$\alpha = (\alpha_1, \ldots, \alpha_p)$. Note that some α_j might be unused; however, taking $\alpha \in \mathbb{R}^p$ avoids the need to explicitly track which nodes appear in the recursive process. The corresponding edge set is

$$\widehat{E}_r(V_0, \alpha) = \bigcup_{s=0}^{r-1} \bigcup_{j \in \widehat{S}_s(V_0,\alpha)} \big\{(j,k) : k \in \widehat{S}_1(j, \alpha_j)\big\},$$

where $\widehat{S}_0(V_0, \alpha) = V_0$ and, for $s \geq 0$, $\widehat{S}_{s+1}(V_0, \alpha) = \widehat{B}_{s+1}(V_0, \alpha) \setminus \widehat{B}_s(V_0, \alpha)$ is the set of nodes that are exactly distance $s+1$ from V_0 in the estimated graph. The graph $\widehat{G}_r(V_0, \alpha) = (\widehat{B}_r(V_0, \alpha), \widehat{E}_r(V_0, \alpha))$ is then an estimator of the true local graph $G_r(V_0)$.

Let $\mathcal{J}_r(V_0)$ and $\widehat{\mathcal{J}}_r(V_0, \alpha)$ be the set of paths starting in V_0 of length r that lie in G and $\widehat{G}_r(V_0, \alpha)$, respectively. A natural extension of Equation S3.1 to a path $\boldsymbol{j}$ of length r starting in V_0 is

$$q(\boldsymbol{j}) = \inf_{\{\alpha : t(\boldsymbol{j}) \leq \alpha\}} \mathbb{E}\left(\frac{|\widehat{\mathcal{J}}_r(V_0, \alpha) \setminus \mathcal{J}(V_0)|}{|\widehat{\mathcal{J}}_r(V_0, \alpha)|} \;\middle|\; |\widehat{\mathcal{J}}_r(V_0, \alpha)| > 0 \right), \tag{S3.4}$$

which is precisely Equation S3.1 when $\boldsymbol{j} = (j, k)$ consists of a single edge. A direct analogue of Theorem S3.1 seems unlikely to hold when $r > 1$ unless no two paths in $\widehat{J}_r(V_0, \alpha)$ contain the same edge because extensions of the independence assumption in Theorem S3.1 to paths will be violated whenever two distinct paths in the estimated graph share an edge. For example, the test statistic and hypothesis pairs for distinct paths (j, k, ℓ) and (j, k, ℓ'), namely $((T_j(k), H_j(k)), (T_k(\ell), H_k(\ell))$ and $((T_j(k), H_j(k)), (T_k(\ell'), H_k(\ell'))$, both contain $(T_j(k), H_j(k))$ and are therefore dependent.

While Equation S3.4 provides a natural extension of Equation S3.1 to paths, it depends on the full sets of estimated and true paths, $\widehat{\mathcal{J}}_r(V_0, \alpha)$ and $\mathcal{J}_r(V_0)$, making it difficult to analyze and interpret. Motivated by the Bayesian interpretation of q-values provided by Theorem S3.1, we instead propose a more path-specific and interpretable measure of uncertainty that assesses the probability that a given path is not in the true graph. Specifically, for any path $\boldsymbol{j} = (j_0, \ldots, j_r)$ starting in V_0 and observations $t_{j_s}(j_{s+1})$ of $T_{j_s}(j_{s+1})$, we define

$$\widetilde{q}(\boldsymbol{j}) = \inf_{\{\alpha : t(\boldsymbol{j}) \leq \alpha\}} \mathbb{P}\big(\boldsymbol{j} \notin \mathcal{J}(V_0) \mid \boldsymbol{j} \in \widehat{\mathcal{J}}_r(V_0, \alpha)\big),$$

where $\{\alpha : t(\boldsymbol{j}) \leq \alpha\}$ is shorthand for $\{\alpha \in \mathbb{R}^p : t_{j_s}(j_{s+1}) \leq \alpha_{j_s} \text{ for all } 0 \leq s < r\}$ and $\mathcal{J}(V_0)$ is the set of all paths in G starting in V_0. This is the smallest probability that $\boldsymbol{j}$ is not in the true graph, given that it is in $\widehat{G}_r(V_0, \alpha)$. Theorem S3.2 states that the uncertainty in the estimated path $\boldsymbol{j}$ is at most the sum of the uncertainties $q_{j_s}(j_{s+1})$ in the neighborhood estimates along it. Its proof, provided below, essentially amounts to a union bound over the individual edges in the path.

Theorem S3.2. *Let $\boldsymbol{j} = (j_0, \ldots, j_r)$ be a path starting in V_0, that is, $j_0 \in V_0$ and $j_s \neq j_t$ for all $s \neq t$. Suppose that the assumptions of Theorem S3.1 hold for every j_s, $0 \leq s < r$, and that $(T_{j_s}(j_{s+1}), H_{j_s}(j_{s+1}))$ are independent for all $0 \leq s < r$. Then $\widetilde{q}(\boldsymbol{j}) \leq \sum_{s=0}^{r-1} q_{j_s}(j_{s+1})$.*

Proof. Observe that $\boldsymbol{j} \notin \mathcal{J}(V_0)$ if and only if $H_{j_s}(j_{s+1}) = 0$ for some $0 \leq s < r$. Also, $\boldsymbol{j} \in \widehat{\mathcal{J}}_r(V_0, \alpha)$ if and only if $T_{j_s}(j_{s+1}) \leq \alpha_{j_s}$ for all $0 \leq s < r$. Therefore,

$$\begin{aligned}
\widetilde{q}(\boldsymbol{j}) &= \inf_{\{\alpha : t(\boldsymbol{j}) \leq \alpha\}} \mathbb{P}\big(\boldsymbol{j} \notin \mathcal{J}(V_0) \mid \boldsymbol{j} \in \widehat{\mathcal{J}}_r(V_0, \alpha)\big) \\
&= \inf_{\{\alpha : t(\boldsymbol{j}) \leq \alpha\}} \mathbb{P}\big(H_{j_s}(j_{s+1}) = 0 \text{ for some } 0 \leq s < r \mid \boldsymbol{j} \in \widehat{\mathcal{J}}_r(V_0, \alpha)\big) \\
&\leq \inf_{\{\alpha : t(\boldsymbol{j}) \leq \alpha\}} \sum_{s=0}^{r-1} \mathbb{P}\big(H_{j_s}(j_{s+1}) = 0 \mid \boldsymbol{j} \in \widehat{\mathcal{J}}_r(V_0, \alpha)\big) \\
&= \inf_{\{\alpha : t(\boldsymbol{j}) \leq \alpha\}} \sum_{s=0}^{r-1} \mathbb{P}\big(H_{j_s}(j_{s+1}) = 0 \mid T_{j_s}(j_{s+1}) \leq \alpha_{j_s}\big) \\
&= \sum_{s=0}^{r-1} \inf_{\{\alpha_{j_s} : t_{j_s}(j_{s+1}) \leq \alpha_{j_s}\}} \mathbb{P}\big(H_{j_s}(j_{s+1}) = 0 \mid T_{j_s}(j_{s+1}) \leq \alpha_{j_s}\big) \\
&= \sum_{s=0}^{r-1} q_{j_s}(j_{s+1}).
\end{aligned}$$

The inequality is a union bound. The third equality holds because the $(T_{j_s}(j_{s+1}), H_{j_s}(j_{s+1}))$ are independent by assumption; specifically, for independent random variables $(X_1, Y_1), \ldots, (X_n, Y_n)$, it is straightforward to verify that Y_1 is conditionally independent of $X_2, \ldots, X_n$ given X_1. The infimum and sum are exchangeable in the fourth equality because each summand only depends on α_{j_s} (not the other α_{j_t}), and the last equality holds by Theorem S3.1. □

S3.3. Additional algorithmic details. In Line 6 of Algorithm 1, the entries Q_{jk} and Q_{kj} are set to the minimum of $q_j(k)$ and $q_k(j)$. This *minimum rule* preserves symmetry and is analogous to the "OR" rule in nodewise graphical models[11], where an edge is included in the estimated graph if either node selects the other as a neighbor. As an alternative, a more conservative *forward rule* may be applied, assigning $Q_{jk} = Q_{kj}$ according to the q-value of the node closer to the initial target set V_0 (still applying the minimum if both nodes lie in the same neighborhood layer). For example, if j and k are distances r and $r + 1$ from V_0, respectively, then Q_{jk} and Q_{kj} are set to $q_j(k)$.

S3.4. Computation. The total runtime of PFS is roughly $p_{\text{sel}} T_{\text{fs}}$, where p_{sel} is the number of nodes whose neighborhoods are estimated, and T_{fs} is the time required for one run of the underlying feature selection method. The quantity p_{sel} depends on the data and the user-specified false discovery thresholds. Liberal thresholds lead to denser neighborhoods and more follow-up selections, while stricter thresholds yield sparser graphs and reduced computation. A practical strategy is to begin with $r_{\max} = 1$ and a liberal threshold to assess neighborhood sparsity around the target variables. Once an appropriate q_1^* is chosen, increase to $r_{\max} = 2$ and rerun PFS using the updated q_1^* along with a liberal value of q_2^* to assess sparsity at radius 2. This process can be repeated iteratively to tune each q_r^* and control the size of the estimated local graph.

In the environmental health study, for example, PFS was run for 15 nodes (two target nodes plus the 13 nodes in their combined neighborhoods), with each IPSS run taking about 60 seconds, yielding a total runtime of approximately 15 minutes. In the breast cancer study, PFS was run for 88 nodes, with each IPSS run taking approximately 35 seconds, yielding a total runtime of approximately 50 minutes. These runtimes are consistent with previous work, which showed that individual IPSS runs complete in under 20 seconds on datasets with 500 samples and 5000 features[12]. In contrast,

two full graph estimation methods with global FDR control, desparsified nodewise scaled lasso[13] (DSNWSL) and Gaussian graphical models with FDR control using scaled lasso[6] (GFCSL), took approximately 4.5 and 5.25 hours on the same breast cancer dataset, respectively. The other three full graph methods in the R package `SILGGM`[7] were computationally infeasible in this setting, as were many of the methods from the R package `bnlearn`[14].

Table S1 shows that PFS with lasso (PFS(L1)) and with gradient boosting (PFS(GB)) scale favorably with dimension relative to many other graph estimation methods. Both PFS variants maintain runtimes on the order of seconds to a few minutes even at $p = 8000$, whereas many full-graph and Markov blanket-based algorithms exceed the imposed two-hour limit well before this regime.

All PFS runtimes reported in this work are based on single-threaded computation, but substantial speedups are easily achievable through parallelization. At each iteration, nodewise selections are performed independently for all nodes in the current layer, enabling straightforward parallel execution. Once neighborhoods are estimated, the pruning step—implemented via the function `prune_graph` in the `localgraph` package—is computationally negligible and can be used to further sparsify the graph without additional feature selection. IPSS is also trivially parallelizable, as each resampling step is independent, allowing for further reductions in runtime[12].

Method	125	250	500	1000	2000	4000	8000
**PFS**(L1)	0:00:06	0:00:15	0:00:13	0:00:02	0:00:16	0:00:24	0:00:13
ARACNE	0:00:00	0:00:00	0:00:00	0:00:00	0:00:01	0:00:06	0:00:28
**PFS**(GB)	0:01:10	0:00:52	0:01:19	0:00:38	0:03:27	0:03:13	0:02:24
NLasso	0:00:02	0:00:03	0:00:07	0:00:29	0:01:45	0:07:35	0:26:19
HPC(L)	0:00:00	0:00:01	0:00:54	0:00:23	0:03:50	0:13:42	0:35:37
GFCSL	0:00:00	0:00:00	0:00:03	0:00:14	0:01:10	0:11:21	1:00:46
DSNWSL	0:00:00	0:00:00	0:00:03	0:00:11	0:01:01	0:15:46	>2hrs
GLasso	0:00:02	0:00:03	0:00:08	0:00:34	0:04:03	0:31:55	>2hrs
MMPC(L)	0:00:00	0:00:00	0:00:02	0:00:21	0:03:00	0:48:32	>2hrs
SIHPC(L)	0:00:00	0:00:00	0:00:02	0:00:27	0:04:34	1:20:06	>2hrs
MMPC	0:00:03	0:00:12	0:00:59	0:06:41	0:41:10	>2hrs	>2hrs
SIHPC	0:00:01	0:00:05	0:00:28	0:05:51	1:26:06	>2hrs	>2hrs
HPC	0:00:09	0:01:37	0:05:24	0:28:55	>2hrs	>2hrs	>2hrs
IAMB(L)	0:00:00	0:00:17	0:16:00	>2hrs	>2hrs	>2hrs	>2hrs
StablePC	0:00:06	0:01:07	0:16:06	>2hrs	>2hrs	>2hrs	>2hrs
FastIAMB(L)	0:00:03	0:00:37	0:28:44	>2hrs	>2hrs	>2hrs	>2hrs
FastIAMB	0:00:08	0:01:12	1:25:45	>2hrs	>2hrs	>2hrs	>2hrs

TABLE S1. *Runtime versus dimension.* Wall-clock runtimes (H:MM:SS) for a single run of different graph estimation methods on simulated data as the number of variables increases from $p = 125$ to 8000 with fixed sample size $n = 500$. Data are generated from a Gaussian graphical model with a random sparse precision matrix. There is one target variable. All methods are run with default settings; PFS(L1) and PFS(GB) denote PFS using IPSS with lasso and gradient-boosting, respectively. PFS parameters are always set to $r_{\max} = 3$ and $q_{\max} = 0.25$. Entries marked >2 hrs exceed the imposed time limit of 2 hours. All other methods are described in Section S5.2. Methods are ordered by runtime at $p = 8000$.

S4. Other methods: Implementation details and settings

In this section, we describe the other graph estimation methods to which PFS is compared in this work. As discussed in Section 1, the following methods belong to one of two classes: regularization-based approaches or constraint-based and mutual-information based approaches. Throughout this work, all method parameters are set to their software-specific defaults unless otherwise specified.

Regularization-based approaches. These methods assume data are Gaussian. The graphical lasso[2] and the nodewise lasso[11] are implemented using the R package `huge`[15]. The bivariate nodewise scaled lasso[16] (BNWSL), desparsified graphical lasso[17] (DSGL), desparsified nodewise scaled lasso[13] (DSNWSL), and Gaussian graphical models with FDR control based on lasso or scaled lasso[6] (GFCL, GFCSL) are implemented using the R package `SILGGM`[7]. The graphical and nodewise lasso do not provide any form of error control; the five `SILGGM` methods provide global FDR control. In several applications, custom nominal FDR values were used for the `SILGGM` methods to yield better results.

Constraint-based and mutual information-based methods. Constraint-based and mutual information-based structure learning algorithms are implemented using the R package `bnlearn`[14]. These include ARACNE[18], hybrid parents and children[19] (HPC), incremental association Markov blanket[20] (IAMB) and its fast variant FastIAMB, max-min parents and children[21] (MMPC), Hiton parents and children[22] (SIHPC), and the stable PC algorithm[23] (StablePC).

In addition to their global implementations, several of these procedures operate nodewise and can be adapted to estimate neighborhoods via the `learn.nbr` or `learn.mb` functions in `bnlearn`; these neighborhoods can then be combined to estimate local graphs. We denote these local variants with a trailing "(L)", for example HPC(L). Methods based on `learn.mb` estimate Markov blankets, whereas those using `learn.nbr` estimate parents-and-children (PC) sets in a directed graph. Markov blanket methods, namely IAMB and FastIAMB, are typically substantially slower than PC-based methods, and both classes scale poorly to high dimensions relative to PFS (Table S1). PC-based algorithms do not directly estimate conditional independence graphs; however, we include them because their skeletons can be interpreted as undirected graphs and they are widely used in practice. Unlike PFS, none of these approaches provide edgewise or pathwise false discovery control.

S5. Additional details: Simulation studies

We conduct five simulation studies to evaluate the performance of PFS relative to existing methods across regimes that vary in sparsity, linearity, and sample size. Specifically, we consider: sparse, linear graphs with $n = 100$ (Table S2); sparse, nonlinear graphs with $n = 100$ (Table S3); dense, linear graphs with $n = 100$ (Table S4); dense, linear graphs with $n = 500$ (Table S5); and dense, nonlinear graphs with $n = 500$ (Table S6). These settings are chosen to assess (i) the behavior of local graph estimators under ideal Gaussian assumptions, (ii) robustness to nonlinear relationships, and (iii) performance when the true local structure around the target variable is dense.

In each study, there are $p = 200$ variables and a single target variable. Each table reports the true positive rate (TPR) and false discovery rate (FDR) (Equations S2.1 and S5.2) for local graph recovery at multiple radii, averaged over 100 independent trials. In Figure S2, we further examine the performance of PFS as the sample size n varies. Full details of the data-generating mechanisms and parameter choices for each setting are provided below.

S5.1. Simulation designs. All simulation studies are based on block-structured conditional independence graphs with a single target variable and $p = 200$ total variables. Data are generated from Gaussian graphical models with precision matrix Θ (Section S2), optionally followed by a nonlinear transformation of the target variable.

Block-structured precision matrices. In all settings, the precision matrix Θ is constructed with three blocks: the first contains only the target variable, the second contains the target's immediate neighbors, and the third block contains all remaining variables. Nonzero entries within and between blocks are generated at random according to prespecified degrees, with values chosen uniformly from $\{-1, 1\}$. The resulting matrix is symmetrized, a positive constant is added to the diagonal to ensure positive definiteness, and the matrix is rescaled so that its eigenvalues lie between 0.01 and 10, allowing for strong correlations between variables as is common in practice.

Sparse, linear, $n = 100$. In the sparse linear setting (Table S2), the three blocks of Θ are sizes 1×1 (target variable), 4×4 (immediate neighbors of the target), and 195×195 (the remaining variables). There are no edges between the four neighbors of the target. Each neighbor is connected to the third block with average degree six, while variables in the third block have average degree two. In each of the 100 trials, a new Θ is generated and $n = 100$ samples are drawn independently from $\mathcal{N}(0, \Theta^{-1})$.

Sparse, nonlinear, $n = 100$. In the sparse nonlinear setting (Table S3), the block structure is identical to the sparse linear case, except that the connectivity between the target's neighbors and the third block is reduced to an average degree of two. The only other difference between this setting and the sparse, linear, $n = 100$ case is that after sampling $n = 100$ observations from $\mathcal{N}(0, \Theta^{-1})$, the target variable X_1 is redefined as a nonlinear function of its true neighbors:

$$X_1 = \sum_{j \in S_1(X_1)} \exp(-X_j^2/2) + \varepsilon, \tag{S5.1}$$

where ε is mean-zero Gaussian noise scaled to achieve a signal-to-noise ratio—defined as the variance of the signal divided by the variance of the noise—of 4. This form of nonlinearity is motivated by scenarios in medicine and epidemiology where both high and low levels of certain covariates can be harmful, making the response sensitive to deviations from a normal range.

Dense, linear, $n = 100$. In the dense linear setting (Table S4), the three blocks of Θ are sizes 1×1 (target variable), 20×20 (immediate neighbors of the target), and 179×179 (the remaining variables). The neighbor block has average internal degree five, and variables in the third block have average degree ten. Each of the 20 immediate neighbors of the target is connected to the third block with average degree five. As before, in each of the 100 trials, a new Θ is generated and $n = 100$ samples are drawn independently from $\mathcal{N}(0, \Theta^{-1})$.

Dense, linear, $n = 500$. In the dense linear setting with increased sample size (Table S5), the block structure and connectivity are identical to the dense, linear, $n = 100$ case. In each of the 100 trials, a new Θ is generated and $n = 500$ samples are drawn independently from $\mathcal{N}(0, \Theta^{-1})$.

Dense, nonlinear, $n = 500$. In the dense nonlinear setting (Table S6), the block structure and connectivity are identical to the dense, linear, $n = 500$ case. The only other difference is that after sampling $n = 500$ observations from $\mathcal{N}(0, \Theta^{-1})$, the target variable X_1 is redefined as a nonlinear function of its true neighbors according to Equation S5.1, where ε is again mean-zero Gaussian noise, scaled to achieve a signal-to-noise ratio of 4.

S5.2. Method settings. All methods are run using default settings from their respective software packages unless otherwise specified. PFS is implemented using integrated path stability selection (IPSS) via the Python package `ipss`[12,24]. In linear settings, IPSS uses lasso[25], while in nonlinear settings it uses variable importance scores from gradient boosting[26]. The maximum pathwise q-value threshold is set to $q^*_{\text{path}} = 0.2$. Local neighborhood q-value thresholds $(q^*_1, q^*_2, \dots)$ are set to $(0.2, 0.1, 0.1, 0.1)$ in linear settings and $(0.2, 0.05, 0.05, 0.05)$ in nonlinear settings. For the `SILGGM` methods, the target FDR is set to 0.1. For `bnlearn` methods, correlation-based tests are used in linear settings and mutual information-based tests are used in nonlinear settings.

S5.3. Simulation results. The results of the five simulation studies are reported in Tables S2 to S6. The false discovery rates for each method at each radius are the average local false discovery proportions (Equation S2.1) over the 100 trials. For an estimated local graph $\widehat{G}_r(V_0) = (\widehat{B}_r(V_0), \widehat{E}_r(V_0))$, the local *true positive rate* (TPR) is the proportion of true edges in the estimated local graph of radius r among all edges in the true local graph $E_r(V_0)$:

$$\text{TPR}(\widehat{E}_r(V_0)) = \frac{|\widehat{E}_r(V_0) \cap E_r(V_0)|}{|E_r(V_0)|}. \tag{S5.2}$$

	TPR				FDR			
Method	$r = 1$	$r = 2$	$r = 3$	$r = 4$	$r = 1$	$r = 2$	$r = 3$	$r = 4$
**PFS**	0.85	0.59	0.31	0.19	0.16	0.07	0.07	0.07
GLasso	0.92	0.95	0.90	0.80	0.90	0.95	0.92	0.89
NLasso	0.90	0.87	0.79	0.71	0.43	0.43	0.47	0.39
BNWSL	0.34	0.15	0.09	0.06	0.01	0.02	0.02	0.02
DSGL	0.03	0.02	0.01	0.01	0.00	0.00	0.00	0.00
DSNWSL	0.60	0.40	0.28	0.22	0.01	0.00	0.01	0.01
GFCL	0.92	0.84	0.71	0.58	0.38	0.40	0.36	0.29
GFCSL	0.60	0.40	0.29	0.25	0.09	0.10	0.13	0.14
ARACNE	0.84	0.77	0.68	0.68	0.15	0.17	0.38	0.64
HPC	0.79	0.67	0.54	0.47	0.02	0.03	0.07	0.13
MMPC	0.80	0.65	0.57	0.55	0.08	0.08	0.21	0.36
SIHPC	0.79	0.63	0.55	0.52	0.06	0.06	0.18	0.34
HPC(L)	0.82	0.71	0.59	0.55	0.07	0.08	0.22	0.32
MMPC(L)	0.88	0.74	0.69	0.74	0.20	0.26	0.54	0.68
SIHPC(L)	0.87	0.71	0.66	0.70	0.17	0.20	0.46	0.62
FastIAMB(L)	0.09	0.04	–	–	0.20	0.22	–	–
IAMB(L)	0.86	0.78	–	–	0.93	0.99	–	–

TABLE S2. *(Linear, sparse, $n = 100$).* True positive rate (TPR) and false discovery rate (FDR) for local graph recovery across methods (rows) and neighborhood radii (columns), averaged over 100 trials. Methods with a trailing (L) are local versions of their global counterparts; for example, HPC(L) is the local version of HPC. FastIAMB(L) and IAMB(L) were only run to radius 2 due to computational constraints.

Method	TPR $r=1$	TPR $r=2$	TPR $r=3$	TPR $r=4$	FDR $r=1$	FDR $r=2$	FDR $r=3$	FDR $r=4$
**PFS**	0.83	0.47	0.30	0.20	0.19	0.22	0.23	0.22
GLasso	0.00	0.00	0.00	0.01	0.05	0.05	0.05	0.05
NLasso	0.00	0.00	0.00	0.00	0.05	0.05	0.04	0.05
BNWSL	0.00	0.00	0.00	0.00	0.04	0.04	0.04	0.04
DSGL	0.00	0.00	0.00	0.00	0.03	0.03	0.03	0.03
DSNWSL	0.00	0.00	0.00	0.00	0.07	0.07	0.06	0.06
GFCL	0.00	0.00	0.01	0.01	0.23	0.21	0.20	0.19
GFCSL	0.00	0.00	0.01	0.01	0.17	0.17	0.15	0.13
ARACNE	0.03	0.04	0.23	0.59	0.98	0.99	0.98	0.94
HPC	0.00	0.00	0.01	0.02	0.34	0.33	0.31	0.27
MMPC	0.01	0.02	0.06	0.18	0.91	0.90	0.87	0.81
SIHPC	0.01	0.02	0.06	0.16	0.90	0.90	0.87	0.81
HPC(L)	0.00	0.00	0.02	0.07	0.52	0.51	0.48	0.42
MMPC(L)	0.02	0.02	0.16	0.49	0.99	0.99	0.96	0.91
SIHPC(L)	0.02	0.02	0.14	0.45	0.98	0.98	0.96	0.91

TABLE S3. *(Nonlinear, sparse, $n = 100$)*. True positive rate (TPR) and false discovery rate (FDR) for local graph recovery across methods (rows) and neighborhood radii (columns), averaged over 100 trials. Methods with a trailing (L) are local versions of their global counterparts; for example, HPC(L) is the local version of HPC.

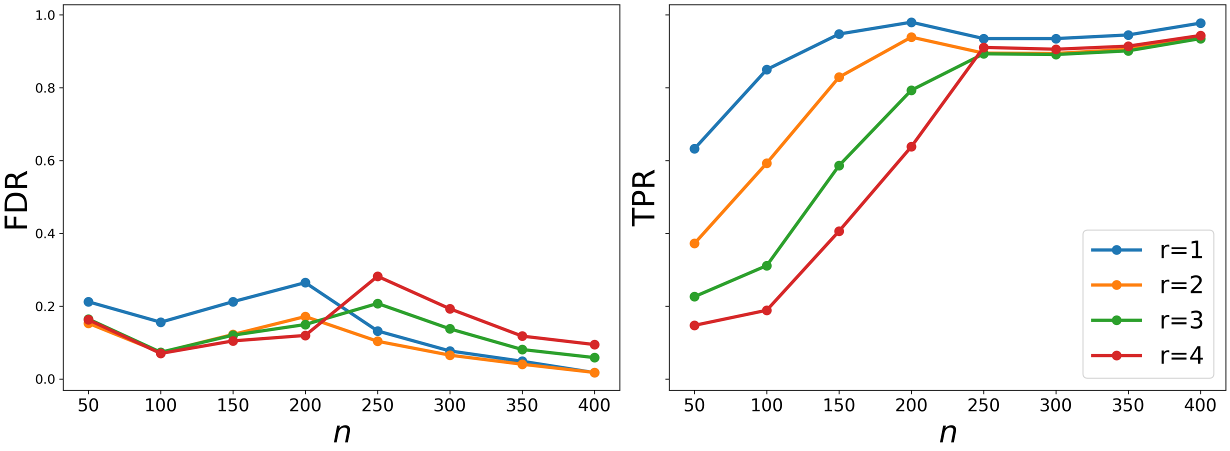

FIGURE S2. *PFS performance as a function of sample size.* FDR (left) and TPR (right) for local graphs estimated by PFS as the sample size n ranges over $\{50, 100, 150, 200, 250, 300, 350, 400\}$. All other simulation settings follow the sparse, linear design in Section S5.1. Curves correspond to radii $r \in \{1, 2, 3, 4\}$; reported FDR and TPR values are averaged over 100 simulation trials.

Method	TPR			FDR		
	$r = 1$	$r = 2$	$r = 3$	$r = 1$	$r = 2$	$r = 3$
**PFS**	0.43	0.13	0.05	0.19	0.21	0.18
GLasso	0.57	0.55	0.52	0.62	0.87	0.80
NLasso	0.42	0.23	0.21	0.29	0.56	0.49
BNWSL	0.01	0.00	0.00	0.00	0.00	0.00
DSGL	0.00	0.00	0.00	0.00	0.00	0.00
DSNWSL	0.07	0.01	0.00	0.02	0.03	0.03
GFCL	0.17	0.05	0.02	0.07	0.14	0.12
GFCSL	0.16	0.05	0.02	0.13	0.22	0.20
ARACNE	0.26	0.08	0.05	0.20	0.38	0.37
HPC	0.17	0.05	0.02	0.06	0.15	0.16
MMPC	0.19	0.06	0.03	0.12	0.26	0.28
SIHPC	0.16	0.05	0.02	0.08	0.21	0.24
HPC(L)	0.22	0.07	0.05	0.12	0.28	0.27
MMPC(L)	0.24	0.10	0.09	0.20	0.46	0.43
SIHPC(L)	0.21	0.08	0.06	0.16	0.37	0.39

TABLE S4. *(Linear, dense, $n = 100$)*. True positive rate (TPR) and false discovery rate (FDR) for local graph recovery across methods (rows) and neighborhood radii (columns), averaged over 100 trials. Methods with a trailing (L) are local versions of their global counterparts; for example, HPC(L) is the local version of HPC.

Method	TPR			FDR		
	$r = 1$	$r = 2$	$r = 3$	$r = 1$	$r = 2$	$r = 3$
**PFS**	0.87	0.59	0.51	0.12	0.16	0.09
GLasso	0.71	0.77	0.91	0.55	0.83	0.70
NLasso	0.63	0.51	0.60	0.22	0.46	0.34
BNWSL	0.79	0.76	0.86	0.06	0.15	0.10
DSGL	0.56	0.45	0.45	0.01	0.03	0.02
DSNWSL	0.75	0.68	0.72	0.01	0.05	0.04
GFCL	0.81	0.76	0.82	0.05	0.14	0.09
GFCSL	0.82	0.78	0.87	0.06	0.15	0.10
ARACNE	0.33	0.11	0.07	0.09	0.18	0.16
HPC	0.44	0.30	0.27	0.02	0.03	0.02
MMPC	0.40	0.25	0.21	0.03	0.07	0.07
HPC(L)	0.48	0.35	0.34	0.03	0.06	0.04
MMPC(L)	0.46	0.34	0.36	0.08	0.21	0.19
SIHPC(L)	0.42	0.29	0.29	0.05	0.15	0.15

TABLE S5. *(Linear, dense, $n = 500$)*. True positive rate (TPR) and false discovery rate (FDR) for local graph recovery across methods (rows) and neighborhood radii (columns), averaged over 100 trials. Methods with a trailing (L) are local versions of their global counterparts; for example, HPC(L) is the local version of HPC.

Method	TPR			FDR		
	$r = 1$	$r = 2$	$r = 3$	$r = 1$	$r = 2$	$r = 3$
**PFS**	0.76	0.48	0.39	0.11	0.24	0.14
GLasso	0.00	0.00	0.02	0.04	0.04	0.03
NLasso	0.00	0.00	0.01	0.04	0.03	0.01
BNWSL	0.01	0.02	0.12	0.52	0.44	0.07
DSGL	0.00	0.00	0.01	0.13	0.11	0.01
DSNWSL	0.00	0.01	0.05	0.33	0.27	0.02
GFCL	0.01	0.02	0.10	0.47	0.40	0.06
GFCSL	0.01	0.02	0.13	0.54	0.46	0.07
ARACNE	0.01	0.01	0.02	0.91	0.82	0.22
HPC	0.00	0.00	0.01	0.19	0.17	0.01
MMPC	0.01	0.01	0.06	0.76	0.68	0.13
HPC(L)	0.01	0.01	0.07	0.76	0.66	0.06
MMPC(L)	0.02	0.02	0.15	0.91	0.83	0.21
SIHPC(L)	0.01	0.01	0.12	0.92	0.82	0.18

TABLE S6. *(Nonlinear, dense, $n = 500$)*. True positive rate (TPR) and false discovery rate (FDR) for local graph recovery across methods (rows) and neighborhood radii (columns), averaged over 100 trials. Methods with a trailing (L) are local versions of their global counterparts; for example, HPC(L) is the local version of HPC.

S5.4. PFS with other selection methods. PFS can be combined with any FDR-controlling procedure, since such procedures can be used to construct q-values. Throughout this work, we use integrated path stability selection (IPSS) for this purpose due to its favorable properties (see Methods) and strong performance relative to other variable selection methods in previous studies[12,24]. In this section, we report PFS results using q-values from other approaches in the same five simulation settings discussed above: (i) linear, sparse, $n = 100$; (ii) nonlinear, sparse, $n = 100$; (iii) linear, dense, $n = 100$; (iv) linear, dense, $n = 500$; (v) nonlinear, dense, $n = 500$. In addition to IPSS, we implemented PFS with the Benjamini–Hochberg (BH) method[27], the Benjamini–Yekutieli (BY) method[28], and three versions of model-X knockoffs[29]: knockoffs with importance statistics from generalized linear models (KOGLM), the lasso (KOL1), and random forests (KORF). Unlike IPSS and knockoffs, BH and BY require p-values; in our experiments, we compute p-values using ordinary least squares (OLS) regression when $n > p$. When $n < p$, we compute p-values using marginal, univariate regressions, which are widely used in practice, though they reflect marginal rather than conditional associations. BH and BY are implemented with the Python package `statsmodels`[30]. The knockoff methods are implemented with the R package `knockoff`.

Tables S7–S11 show that IPSS consistently outperforms other q-value constructions across simulation settings. In all experiments, the PFS framework is held fixed across methods: each method uses the same pathwise q-value threshold q^*_{path} and local thresholds $(q^*_1, q^*_2, \dots)$ specified in Section S5.2. In the two nonlinear experiments, PFS with IPSS significantly outperforms PFS with other selection methods—including KORF, which uses random forests and is therefore also capable of capturing nonlinear relationships—maintaining the highest TPR across all radii while controlling the FDR in both sparse and dense cases. In the two linear, $n = 100$ settings, PFS with BH and BY achieve high TPR but also exceedingly high FDR, while in the linear, dense, $n = 500$ experiment, both methods control FDR but have a lower TPR than PFS with IPSS. PFS with the three knockoff approaches has lower TPR and higher FDR than IPSS in the linear, sparse, $n = 100$ experiment; similar or lower TPR and higher FDR than IPSS in the linear, dense, $n = 100$ experiment; and lower TPR and slightly lower FDR than IPSS in the linear, dense, $n = 500$ experiment.

	TPR				FDR			
Method	$r = 1$	$r = 2$	$r = 3$	$r = 4$	$r = 1$	$r = 2$	$r = 3$	$r = 4$
**IPSS**	0.85	0.59	0.31	0.19	0.16	0.07	0.07	0.07
KOGLM	0.47	0.11	0.05	0.03	0.29	0.26	0.26	0.26
KOL1	0.43	0.11	0.05	0.03	0.25	0.23	0.23	0.23
KORF	0.33	0.11	0.06	0.03	0.46	0.46	0.45	0.45
BH	0.97	0.96	0.95	0.92	0.96	0.99	0.98	0.97
BY	0.96	0.95	0.91	0.86	0.94	0.98	0.98	0.96

Table S7. *PFS with different selection methods (linear, sparse, $n = 100$).* True positive rate (TPR) and false discovery rate (FDR) for local graph recovery across methods (rows) and neighborhood radii (columns), averaged over 100 trials. PFS with integrated path stability selection[12,24] (IPSS) is the method used throughout this work and the default option in the `localgraph` software package. Other methods are: model-X knockoffs[29] with importance statistics based on (i) generalized linear models (KOGLM), (ii) the lasso (KOL1), and (iii) random forests (KORF); the Benjamini–Hochberg method[27] (BH); the Benjamini–Yekutieli method[28] (BY).

Method	TPR				FDR			
	$r = 1$	$r = 2$	$r = 3$	$r = 4$	$r = 1$	$r = 2$	$r = 3$	$r = 4$
**IPSS**	0.83	0.47	0.30	0.20	0.19	0.22	0.23	0.22
KOGLM	0.00	0.00	0.00	0.00	0.01	0.01	0.01	0.01
KOL1	0.00	0.00	0.00	0.00	0.00	0.00	0.00	0.00
KORF	0.13	0.03	0.02	0.01	0.11	0.11	0.11	0.11
BH	0.01	0.01	0.03	0.06	0.13	0.12	0.13	0.13
BY	0.00	0.00	0.01	0.01	0.03	0.03	0.03	0.03

Table S8. *PFS with different selection methods (nonlinear, sparse, $n = 100$).* True positive rate (TPR) and false discovery rate (FDR) for local graph recovery across methods (rows) and neighborhood radii (columns), averaged over 100 trials. PFS with integrated path stability selection[12,24] (IPSS) is the method used throughout this work and the default option in the `localgraph` software package. Other methods are: model-X knockoffs[29] with importance statistics based on (i) generalized linear models (KOGLM), (ii) the lasso (KOL1), and (iii) random forests (KORF); the Benjamini–Hochberg method[27] (BH); the Benjamini–Yekutieli method[28] (BY).

Method	TPR			FDR		
	$r = 1$	$r = 2$	$r = 3$	$r = 1$	$r = 2$	$r = 3$
**IPSS**	0.43	0.13	0.05	0.19	0.21	0.18
KOGLM	0.40	0.07	0.02	0.34	0.37	0.36
KOL1	0.41	0.07	0.02	0.37	0.41	0.38
KORF	0.15	0.02	0.00	0.34	0.36	0.36
BH	0.93	0.81	0.79	0.88	0.99	0.95
BY	0.82	0.68	0.67	0.85	0.98	0.95

Table S9. *PFS with different selection methods (linear, dense, $n = 100$).* True positive rate (TPR) and false discovery rate (FDR) for local graph recovery across methods (rows) and neighborhood radii (columns), averaged over 100 trials. PFS with integrated path stability selection[12,24] (IPSS) is the method used throughout this work and the default option in the `localgraph` software package. Other methods are: model-X knockoffs[29] with importance statistics based on (i) generalized linear models (KOGLM), (ii) the lasso (KOL1), and (iii) random forests (KORF); the Benjamini–Hochberg method[27] (BH); the Benjamini–Yekutieli method[28] (BY).

	TPR			FDR		
Method	$r = 1$	$r = 2$	$r = 3$	$r = 1$	$r = 2$	$r = 3$
**IPSS**	0.87	0.59	0.51	0.12	0.16	0.09
KOGLM	0.35	0.06	0.01	0.06	0.07	0.06
KOL1	0.31	0.05	0.01	0.06	0.06	0.06
KORF	0.02	0.00	0.00	0.02	0.02	0.02
BH	0.57	0.28	0.20	0.19	0.26	0.16
BY	0.26	0.07	0.03	0.03	0.04	0.03

TABLE S10. *PFS with different selection methods (linear, dense, $n = 500$).* True positive rate (TPR) and false discovery rate (FDR) for local graph recovery across methods (rows) and neighborhood radii (columns), averaged over 100 trials. PFS with integrated path stability selection[12,24] (IPSS) is the method used throughout this work and the default option in the `localgraph` software package. Other methods are: model-X knockoffs[29] with importance statistics based on (i) generalized linear models (KOGLM), (ii) the lasso (KOL1), and (iii) random forests (KORF); the Benjamini–Hochberg method[27] (BH); the Benjamini–Yekutieli method[28] (BY).

	TPR			FDR		
Method	$r = 1$	$r = 2$	$r = 3$	$r = 1$	$r = 2$	$r = 3$
**IPSS**	0.76	0.48	0.39	0.11	0.24	0.14
KOGLM	0.00	0.00	0.00	0.00	0.00	0.00
KOL1	0.00	0.00	0.00	0.00	0.00	0.00
KORF	0.03	0.00	0.00	0.02	0.02	0.02
BH	0.00	0.00	0.00	0.17	0.14	0.04
BY	0.00	0.00	0.00	0.03	0.03	0.03

TABLE S11. *PFS with different selection methods (nonlinear, dense, $n = 500$).* True positive rate (TPR) and false discovery rate (FDR) for local graph recovery across methods (rows) and neighborhood radii (columns), averaged over 100 trials. PFS with integrated path stability selection[12,24] (IPSS) is the method used throughout this work and the default option in the `localgraph` software package. Other methods are: model-X knockoffs[29] with importance statistics based on (i) generalized linear models (KOGLM), (ii) the lasso (KOL1), and (iii) random forests (KORF); the Benjamini–Hochberg method[27] (BH); the Benjamini–Yekutieli method[28] (BY).

S6.1. Data sources. Cancer incidence and mortality rates, along with smoking prevalence and cancer screening data, were obtained from the State Cancer Profiles platform, a joint initiative of the National Cancer Institute (NCI) and Centers for Disease Control and Prevention (CDC). This platform aggregates geographically resolved cancer statistics from national systems for public health planning. Cancer incidence and mortality were aggregated over 2017–2022, while screening and smoking data were drawn from 2017–2019. County-level demographic data—including race/ethnicity, sex, and age—were sourced from the U.S. Census Bureau using the 2000 decennial census.

The majority of covariates were drawn from the Environmental Protection Agency's (EPA) Environmental Quality Index (EQI) dataset, specifically the 2000–2005 release[31]. The EQI compiles county-level indicators across five domains—air, water, land, built environment, and sociodemographic factors—from diverse federal and commercial sources, including the EPA, U.S. Geological Survey, Department of Agriculture, and Federal Bureau of Investigation (FBI). To improve comparability across counties, variables were often log-transformed to reduce skew, kriged to fill spatial gaps, or rescaled for consistency in valence. Additional details about the EQI dataset are provided in "*Construction of an environmental quality index for public health research*," by Messer et al.[31]. The period 2000–2005 was chosen for most covariates to reflect the latency of cancer development[31–34].

S6.2. Data cleaning. The raw dataset consisted of $n = 3141$ counties and $p = 240$ variables aggregated from the sources described above. Indiana and Kansas were excluded because they did not report incidence data in the State Cancer Profiles dataset. We also removed Union County, Florida due to extreme outlier values in both cancer incidence and mortality. Some covariates were sparsely reported across large regions: variables missing from five or more states, as well as variables missing in at least 1500 counties were removed. This resulted in our final dataset consisting of $n = 2857$ counties and $p = 165$ variables.

S6.3. PFS implementation. We applied PFS using random forest variable importance scores, specifically mean decrease in impurity (MDI), as implemented in the `ipss` Python package with its default settings[12,24]. Connections between environmental exposures tended to be slightly stronger than those between socioeconomic variables. Thus, to improve interpretability in specific neighborhoods and in the estimated local graph as a whole, we customized q-value thresholds for a few variables based on prior relevance or notable patterns observed during analysis. The neighborhood q-value threshold for incidence was set to 0.04. We also increased thresholds for Hispanic (0.01), Poverty (0.011), and Smoking (0.01). In the case of Education, where multiple variables had the same lowest q-value, we broke ties using expected false positive (efp) scores[12]. For all remaining variables, the default neighborhood q-value threshold was 0.008. In the case of graphical lasso, all variables were standardized to have mean 0 and variance 1. Both the PFS and graphical lasso graphs were estimated with a fixed random seed to ensure reproducibility.

S6.4. Supporting analyses. Below we present additional tables and figures that complement the main analysis in Section 2.4. These include carcinogenicity classifications of key environmental exposures identified in the local graph around cancer incidence (Table S12), partial correlation results supporting the potential mediating role of poverty in racial disparities in mortality (Table S13), and a nonlinear visualization of associations among environmental factors (Figure S3).

Exposure	Formula	IARC	IRIS	Common sources
$PM_{2.5}$	–	1	–	Combustion (gasoline, diesel, oil, wood)
Acrylonitrile	C_3H_3N	1	2	Plastic and rubber manufacturing
TCE	C_2HCl_3	1	1	Industrial degreasing
Diesel	–	1	2	Diesel engine exhaust
EtO	C_2H_4O	1	1	Chemical manufacturing
Hydrazine	N_2H_4	2A	2	Rocket fuels, polymer foam production
Quinoline	C_9H_7N	2B	2	Chemical manufacturing (dyes, pesticides, solvents)
DEHP	$C_24H_38O_4$	2B	2	Plastic manufacturing
DBCP	$C_3H_5Br_2Cl$	2B	–	Pesticides (banned in contiguous U.S. in 1979)
Hg	Hg	3	–	Industrial manufacturing, pesticides
SO_2	SO_2	3	–	Combustion (coal and oil)

TABLE S12. *Carcinogenicity classifications of environmental exposures identified by PFS in the local graph around cancer incidence (Figure 2).* Many of the exposures connected to cancer incidence are classified as known or likely human carcinogens. IARC categories: Group 1 = carcinogenic to humans; 2A = probably carcinogenic; 2B = possibly carcinogenic; 3 = unclassifiable. IRIS categories: 1 = carcinogenic to humans; 2 = likely to be carcinogenic. Dashes indicate no classification is available.

Target	Covariate	Adjusted for	ρ (p-value)	ρ_{adj} (p-value)
Incidence	Hispanic	Screening	-0.340 ($< 10^{-10}$)	-0.324 ($< 10^{-10}$)
Incidence	Hispanic	Mercury	-0.340 ($< 10^{-10}$)	-0.174 ($< 10^{-10}$)
Mortality	Black	Poverty	0.228 ($< 10^{-10}$)	0.053 (0.005)
Mortality	Poverty	Black	0.432 ($< 10^{-10}$)	0.380 ($< 10^{-10}$)
Mortality	White	Poverty	-0.059 (0.002)	0.249 ($< 10^{-10}$)
Mortality	Poverty	White	0.432 ($< 10^{-10}$)	0.484 ($< 10^{-10}$)

TABLE S13. *Partial correlations between variables in the county-level cancer study.* For each target-covariate pair, we report the Pearson correlation ρ and the partial correlation ρ_{adj} after adjusting for a third variable. These results support the potential mediating role of poverty in racial disparities in mortality, and suggest that reduced mercury exposure may partially explain lower cancer incidence in counties with large Hispanic populations, whereas screening differences do not.

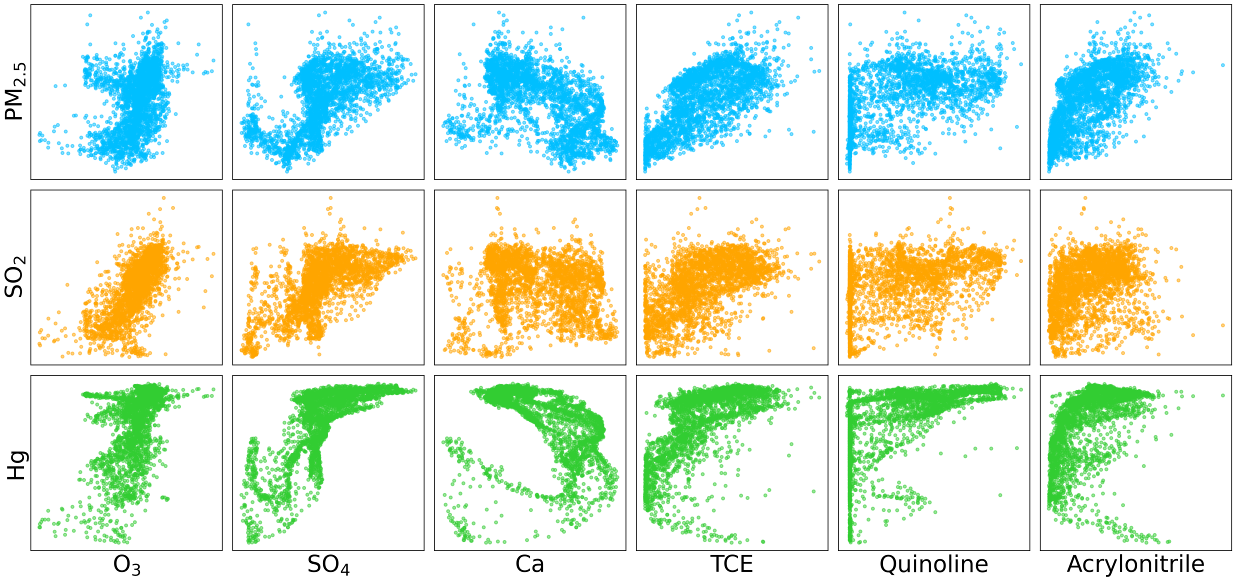

Figure S3. *Nonlinear associations among environmental exposures.* Scatter plots show marginal pairwise relationships between $PM_{2.5}$ (row 1), SO_2 (row 2), and Hg (row 3), and six other exposures—O_3, SO_4, Ca, TCE, Quinoline, and Acrylonitrile—that are present in the local graph in Figure 2. Several of the relationships exhibit nonlinear patterns, including strong curvature. These findings highlight the limitations of linear modeling assumptions and support the use of nonparametric methods for analyzing high-dimensional environmental data.

S7.1. Data sources. All data were obtained from The Cancer Genome Atlas breast cancer cohort (TCGA-BRCA) and downloaded from the publicly available LinkedOmics platform[35]. The dataset includes gene expression measured by bulk RNA sequencing (RNAseq), microRNA (miRNA) expression, protein abundance profiled using reverse phase protein arrays (RPPA), and clinical information. Bulk RNAseq data are provided at the gene level, normalized as $\log_2(\text{RSEM} + 1)$ expression values. The raw RNAseq matrix contains 1093 tumor samples across 20,155 genes. miRNA data, derived from the Illumina HiSeq platform and provided as $\log_2(\text{RPM} + 1)$ normalized values, include 755 samples across 823 miRNAs. RPPA data contain log-normalized protein measurements across 887 samples and 175 proteins. The three clinical target variables are (i) pathologic stage (stage I, II, III, or IV), (ii) histological type, filtered to the two most common categories, namely invasive ductal carcinoma (IDC) and invasive lobular carcinoma (ILC), and (iii) survival status at last follow-up (in the cleaned dataset, 76 patients were deceased and 471 were alive).

S7.2. Data cleaning. We retained genes whose mean expression or variance exceeded the 75th percentile of the gene-wise distributions, thereby removing genes with relatively low expression and variability. To integrate the modalities, we first identified samples with complete measurements across the RNAseq, miRNA, and RPPA data, as well as the three clinical variables. We then removed features with absolute pairwise correlation exceeding 0.999, retaining only one representative column from each highly correlated pair. Columns containing any missing values were removed, and samples with missing values in the response variables were also excluded. After these steps, the final analysis set consists of $n = 547$ tumor samples with $p = 10{,}744$ features, spanning 9785 genes, 819 miRNAs, 137 proteins, and the three clinical targets.

S7.3. PFS implementation. PFS was implemented using IPSS with variable importance scores computed from gradient boosting using XGBoost[26], which is the default choice in the `localgraph` package. Due to the high dimensionality of the data, we increased the number of subsamples in the IPSS algorithm to $B = 200$ (default $B = 100$) to improve robustness. The IPSS preselection expansion factor was set to 2.5 (default 1.5) to retain a broader pool of candidate features before final selection. All other IPSS hyperparameters were left at their default settings.

The maximum radius in the PFS algorithm was set to 3. The pathwise q-value threshold q^*_{path} was set to 1, effectively delegating all thresholding to the neighborhood level. For the three clinical target variables, neighborhood q-value thresholds were set to 0.25. For non-target features, an intermodal q-value threshold of 0.035 was applied; that is, an edge was included between nodes from different modalities if its q-value was below this cutoff. For intramodal edges—that is, for edges between nodes of the same type—all nodes in $S_1(V_0)$ had an intramodal neighborhood q-value threshold of 0.025, while all nodes in $S_2(V_0)$ had an intramodal neighborhood q-value threshold of 0.0175.

S7.4. Supporting analyses. In addition to reviewing the literature, we performed two post hoc validation analyses to assess the biological coherence of structures in the local graph estimated by PFS (Figure 4). These analyses were not used to construct the graph, tune parameters, or select features, but rather to provide external statistical validation of the estimated graph.

Gene set ORA. For selected gene clusters extracted from the estimated local graph, we conducted over-representation analysis (ORA) using curated gene sets from the Molecular Signatures Database (MSigDB) C2 collection (v2025.1), which includes gene sets derived from pathway databases, genetic

perturbation experiments, and the biomedical literature[36]. ORA was performed using the `gseapy`[37] implementation of Fisher's exact test, with the background defined as all 9785 genes in the dataset to which PFS was applied. For each cluster, gene sets were ranked by FDR-adjusted p-value. Additional details and results, including lists of genes in each cluster, are in Section S7.4.

Protein-protein interaction support. To assess whether protein-protein edges inferred by PFS are supported by existing biological knowledge, we compared inferred protein-protein edges against the STRING v12.0 protein interaction database[38]. We extracted all protein-protein edges from the estimated local graph and recorded the number of edges with a STRING interaction score of at least 400, which is the database's default threshold for moderate to high-confidence interactions. To evaluate enrichment relative to chance, we constructed a null distribution by repeatedly sampling the same number of protein pairs uniformly at random from the set of all measured proteins and computing the number of sampled pairs exceeding the STRING score threshold. This procedure was repeated 100,000 times to obtain an empirical null distribution. Enrichment was quantified by the fold increase in supported edges relative to the null expectation, and significance was assessed using an empirical p-value. The same procedure was applied to graphs inferred by alternative methods for comparison. Detailed results are reported in Section S7.4.

We report additional results from over-representation analyses (ORAs) conducted on gene modules extracted from the estimated local graph in the TCGA breast cancer study (Section 2.5). These analyses provide set-level statistical validation of the molecular neighborhoods surrounding the clinical target variables, complementing the literature-based interpretation of individual genes and edges presented in the main text.

For each module, ORA was performed using curated gene sets from MSigDB (C2 collection). Reported results correspond to the top-ranked enriched gene sets for each module, ordered by FDR-adjusted p-value. Only gene sets with overlap at least three were considered. Here, *overlap* denotes the number of genes shared between a given module and a gene set, relative to the total size of that gene set. For modules exhibiting multiple related enrichments (e.g., HOOK3), we report representative results capturing distinct biological themes.

We report results for the following clusters of genes in the local graph estimated by PFS (Figure 4); each cluster is named by a variable in the cluster that links directly to a target in the estimated graph: ANKLE2 cluster (ANKLE2, GOLGA3, EP400, POLE, SETD1B, MLL2, C12orf51, ULK1, SFRS8); HOOK3 cluster (HOOK3, QKI, SGK196, MYST3, MAP4K5, FNTA, HGSNAT, AGPAT6, GOLGA7, IKBKB, VDAC3, WHSC1L1, SLC20A2); miR-210 cluster (P4HA1, ISCU, RIC8A, CA9, EPS8L2, BTNL9, NDRG1, CD151, PDDC1, PKP3, PHRF1, BET1L, NAP1L4); CDH1 cluster (CDH1, CES2, ABHD1, CES8, KIAA0174, AP1G1, USP10, DYNC1LI2, CTCF, ATXN1L, ATMIN, AIP, TERF2IP, GABARAPL2); and miR-133a-1 cluster (FBXO2, ACTC1, DES, MYH11, LOC728264).

Cluster	Gene set	Adjusted p-value
ANKLE2	NIKOLSKY_BREAST_CANCER_12Q24_AMPLICON	8.6×10^{-13}
HOOK3	NIKOLSKY_BREAST_CANCER_8P12_P11_AMPLICON	3.3×10^{-13}
HOOK3	NIKOLSKY_MUTATED_AND_AMPLIFIED_IN_BREAST_CANCER	9.8×10^{-3}
HOOK3	CREIGHTON_ENDOCRINE_THERAPY_RESISTANCE_5	4.8×10^{-2}
CDH1	PROVENZANI_METASTASIS_UP	1.5×10^{-4}
miR-210	BUFFA_HYPOXIA_METAGENE	5.4×10^{-3}
miR-133a-1	REACTOME_MUSCLE_CONTRACTION	3.5×10^{-3}

Table S14. *Over-representation analysis (ORA) results for gene modules extracted from the estimated local graph in TCGA breast cancer data.* For each module, we report the top enriched gene sets ranked by FDR-adjusted p-value. Only gene sets with minimum overlap 3 were considered, where *overlap* is defined as the number of genes shared between the module and the corresponding gene set.

	Total	Supported	Expected	Fold enrichment	p-value
**PFS**	34	22	6.82	3.228	10^{-5}
ARACNE	25	5	5.01	0.998	0.580
GFCSL	112	15	22.43	0.669	0.974
GFCSL(NPN)	388	66	77.76	0.849	0.942
DSNWSL	0	0	0	0	–
DSNWSL(NPN)	0	0	0	0	–
NLasso	0	0	0	0	–
NLasso(NPN)	0	0	0	0	–

Table S15. *Support of inferred protein-protein interactions.* For each method, we report the total number of inferred protein-protein edges, the number of inferred edges supported by a STRING[38] score of at least 400 (the default STRING threshold for moderate-to-high confidence), the expected number of supported edges under a null model based on randomly sampled protein pairs, the corresponding fold enrichment, and an empirical p-value computed from 100,000 random draws. Methods with zero inferred protein-protein edges are shown for completeness.

S8.1. Data sources. Subject-level data together with the official Human Connectome Project[39] (HCP) data dictionary were obtained from the HCP Young Adult (S1200) release and downloaded from the public repository `https://balsa.wustl.edu`.

S8.2. Data cleaning. We began with the subject-level HCP dataset and official data dictionary. All cognition variables were removed except the target (Fluid Cognition Composite). Bookkeeping and metadata variables (e.g., subject identifiers, acquisition details, scanner and session fields, completeness indicators) were excluded. To ensure consistency, only age-adjusted phenotype versions were retained. We restricted behavioral variables to summary-level measures, including working memory, emotion, language, motor, sensory, and personality summary scores. Structural variables were limited to FreeSurfer[40] morphometry features, identified by the prefix `FS_`. Only numeric variables were retained. Subjects missing the target were removed. Features with zero variance were dropped. Remaining missing values were mean-imputed columnwise. After cleaning, the final dataset consists of $n = 1188$ individuals and $p = 213$ variables.

S8.3. PFS implementation. The maximum radius was set to 3 and local q-value thresholds at radii 1, 2, and 3 were set to 0.15, 0.03, and 0.02, respectively. All other PFS parameters were set to their default values in the `localgraph` package.

S8.4. Supporting analyses. To interpret cortical structure recovered in the local graphs estimated by PFS and other methods, we performed over-representation analysis (ORA) of Yeo-7 functional systems[41]. Results are reported in Table 1. For each method we extracted the set of cortical Desikan-Killiany (DK) regions[42] appearing in the local graph component anchored at the node directly connected to the target. Only cortical FreeSurfer morphometry features (thickness and surface area) were included[40]; subcortical and non-morphometric variables were excluded from enrichment testing.

Each DK region was mapped to a Yeo-7 network using a vertex-count lookup table assigning each parcel to its dominant functional system[43]. The background consists of all cortical DK regions present in the cleaned dataset. Enrichment p-values are computed using an upper-tail hypergeometric test. Thus, ORA tests whether a functional system is represented in the local graph more often than expected under random sampling of cortical regions, linking recovered structural organization to established large-scale functional systems implicated in cognition. Enrichment outputs and gene lists are provided with the accompanying code release.

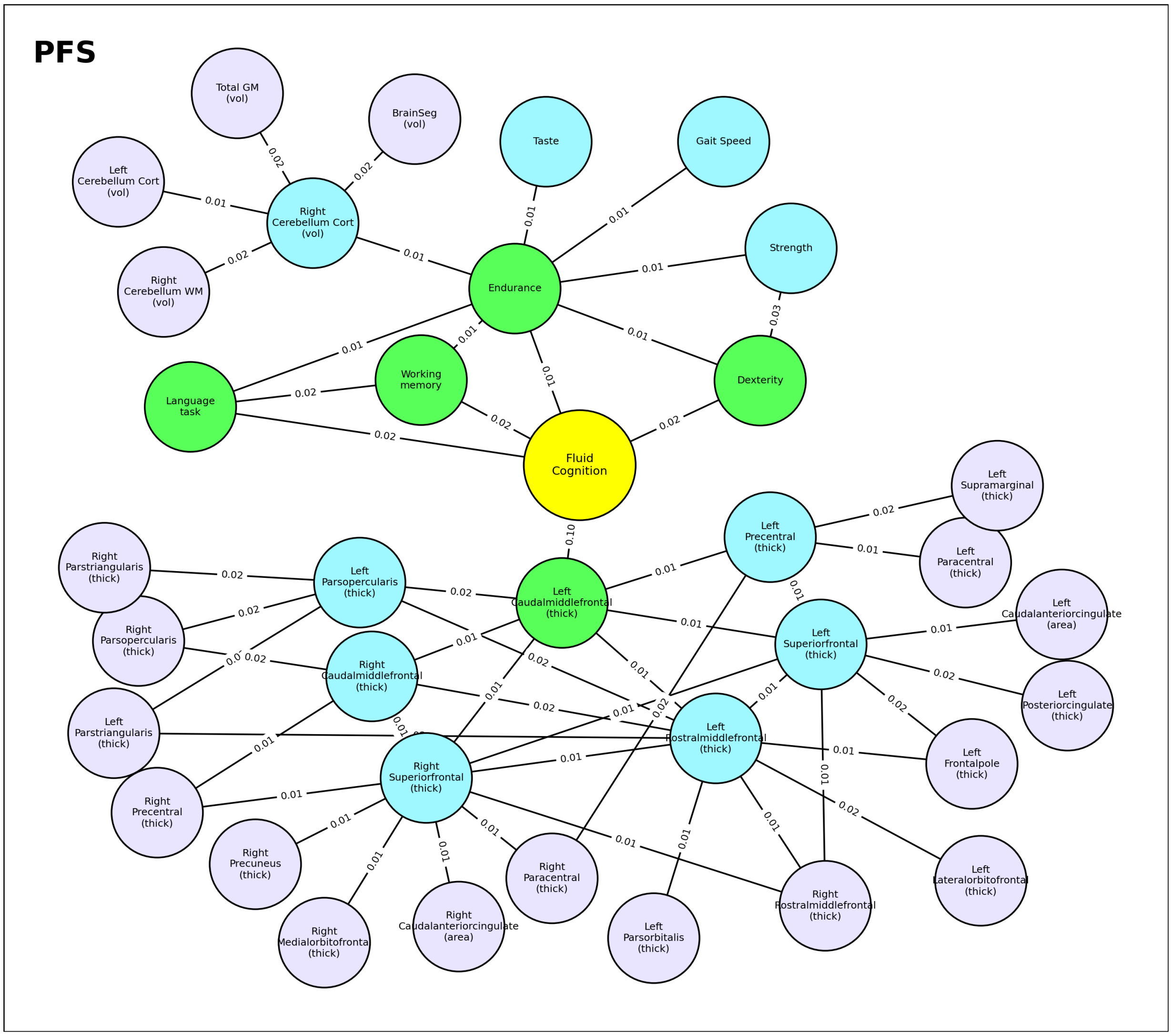

Figure S4. *Radius 3 graph around fluid cognition for the Human Connectome Project data, estimated by PFS.* The target variable, Fluid Cognition score, is shown in yellow. Nodes directly connected to the target are shown in green, nodes at distance two are shown in blue, and nodes at distance three are shown in purple. Edges are annotated with q-values, with smaller values indicating stronger evidence of conditional dependence. Thickness, area, and volume measurements of specific brain regions are labeled with “(thick)”, “(area)”, and “(volume)” respectively.

S9.1. Data sources. Single-nucleus RNA-sequencing data from the study by Grubman et al.[44] were downloaded from https://adsn.ddnetbio.com. The dataset includes gene expression profiles for nuclei from six Alzheimer's disease (AD) and six control individuals, together with cell-type annotations and metadata. Analyses were conducted separately within astrocytes ($n = 2171$ cells), microglia ($n = 449$), and oligodendrocyte progenitor cells (OPCs; $n = 1078$). The target variable in each analysis was a binary indicator of AD versus control status.

S9.2. Data cleaning. Raw count matrices (genes $\times$ nuclei), gene identifiers, and cell barcodes were aligned with the provided metadata. Genes detected in fewer than 50 nuclei were removed. Library-size normalization was performed by scaling each nucleus to 10,000 total counts, followed by $\log(1 + x)$ transformation. After filtering, the analysis matrices contained $p = 10{,}788$ genes (astrocytes), $p = 10{,}513$ genes (microglia), and $p = 10{,}772$ genes (OPCs).

S9.3. PFS implementation. PFS was applied separately within each cell type, with maximum radius 3 in each case. For astrocytes, the local q-value thresholds were set to 0.025 at all radii. For microglia and OPCs, local q-value thresholds were set to 0.05, 0.1, and 0.1 at radii 1, 2, and 3, respectively. All other parameters were set to their defaults in the `localgraph` package.

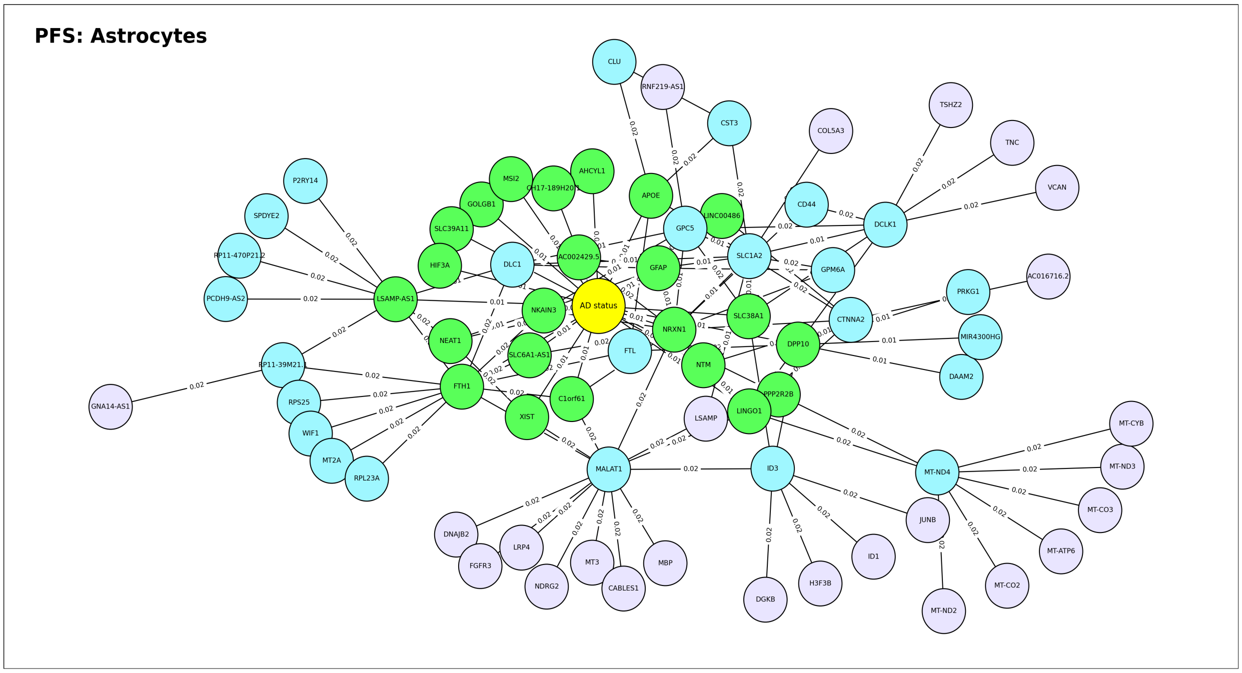

Figure S5. *PFS graph of radius 3 around AD status in astrocytes.* The target (AD status) is shown in yellow. Genes directly connected to the target are shown in green, distance-2 genes are blue, and distance-3 genes are purple. Edges are annotated with q-values, with smaller values indicating stronger evidence of conditional dependence.

S9.4. Supporting analyses. We performed gene set over-representation analyses (ORAs) to assess the biological coherence of the PFS local graphs around AD status for each cell type. Results are

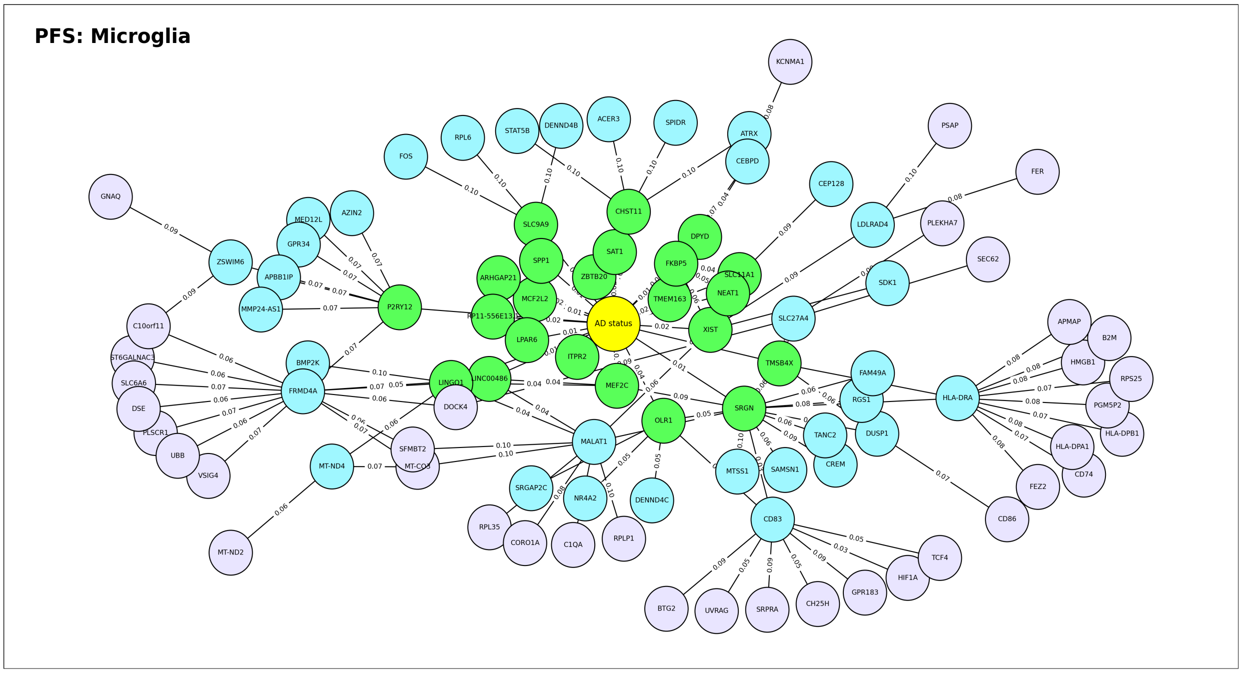

FIGURE S6. *PFS graph of radius 3 around AD status in microglia*. Visual encoding and statistical interpretation are as in Figure S5.

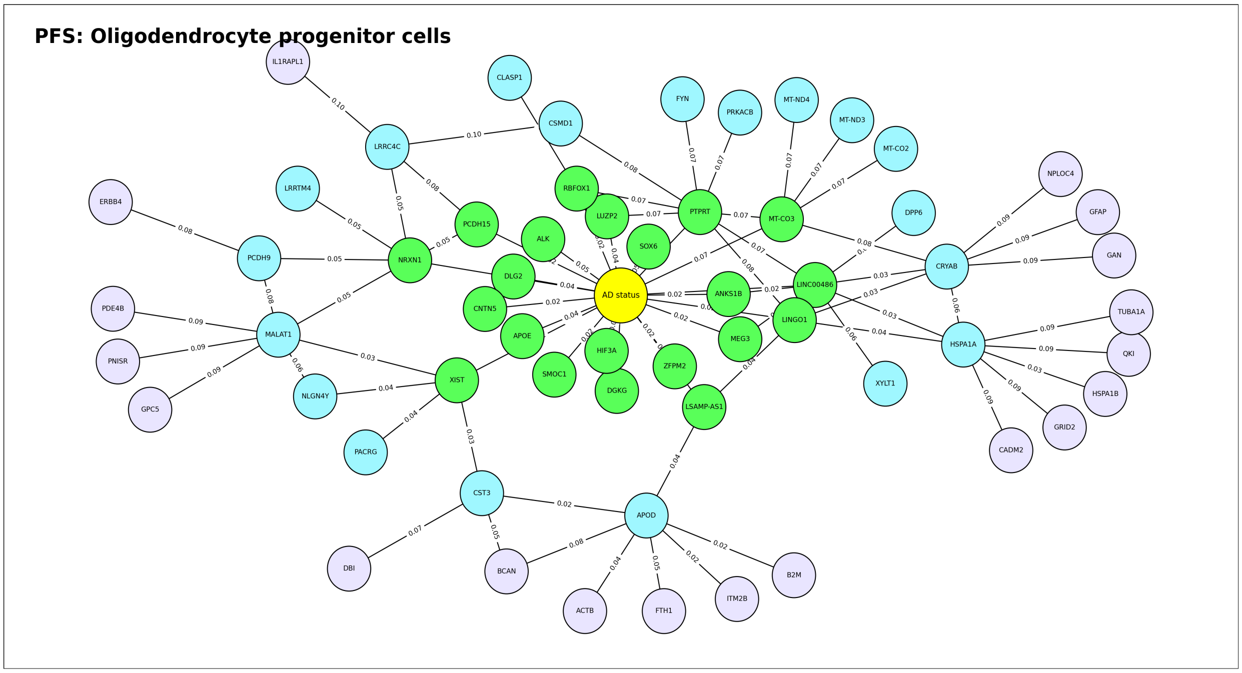

FIGURE S7. *PFS graph of radius 3 around AD status in oligodendrocyte progenitor cells (OPCs)*. Visual encoding and statistical interpretation are as in Figure S5.

reported in Table 2. As with the other ORAs presented in this work, these analyses serve as external statistical validation of the estimated local structures.

Gene clusters consist of all genes in the local graph of radius 3 around AD status for each cell type. ORA was performed using curated gene sets from the MSigDB C2 Reactome and C5 Gene Ontology Biological Process collections (v2025.1) via the `gseapy` implementation of Fishers exact test[36,37]. The background set is composed of all genes retained after cell-type-specific preprocessing (Section S9.2). Gene sets were filtered to require minimum overlap of 5 genes. FDR-adjusted p-values are reported. Enrichment outputs and gene lists are provided with the accompanying code release.

In Section S2 we provided theoretical explanations for why certain global graph estimation methods can struggle to recover local structure, and in Section S5 we presented simulation results illustrating these limitations when the true graph is known. In this section, we examine how several global graph estimation methods perform in practice when applied to the same county-level cancer and breast cancer datasets that we analyzed with PFS in Sections 2.4 and 2.5. We apply methods that assume Gaussian data to both the original data and to a nonparanormal transformation of the data, which is designed to relax the Gaussian assumption[4]. Nonparanormal transformations were implemented using the R package `huge`.

S10.1. County-level cancer study. We report results from ten methods applied to the same county-level cancer dataset that PFS was applied to in Section 2.4. The graphical lasso and nodewise lasso are applied with default settings using `huge`. The five methods with global FDR control—BNWSL, DSGL, DSNWSL, GFCL, and GFCSL—were applied using the R package `SILGGM`. For these methods, the target FDR was set to 0.001, substantially more stringent than the `SILGGM` defaults of 0.05 or 0.1. ARACNE, MMPC, and the stable PC algorithm (PC) were applied using the R package `bnlearn`. For these three methods, we used mutual information rather than correlation to test for conditional independence due to observed nonlinearities in the data (Figure S3). We attempted to run several other methods from `bnlearn`, namely Fast IAMB, HPC, SIHPC, but these failed to return results within the allotted time limit of 24 hours. The following figures show estimated local graphs of radius 2 around cancer incidence and mortality for each respective method.

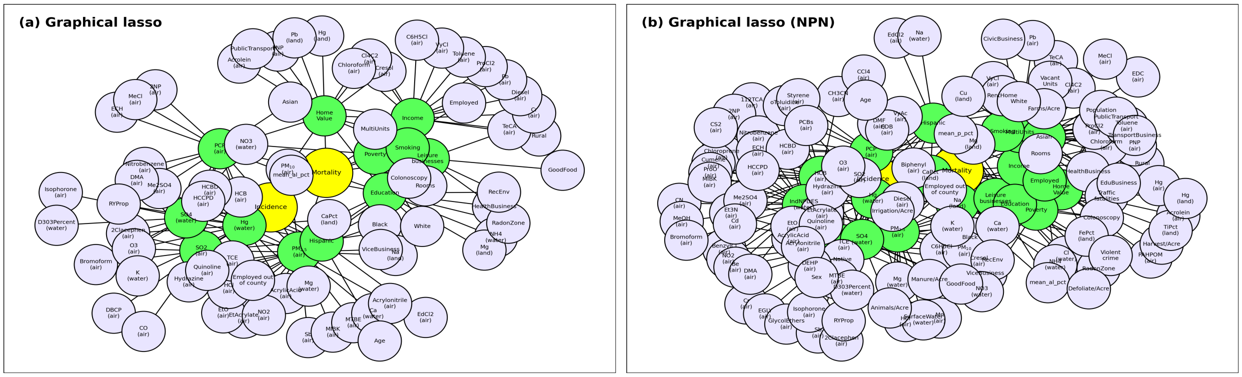

Figure S8. *County-level cancer results using graphical lasso.* Estimated local graph of radius 2 using **(a)** original data and **(b)** nonparanormal data.

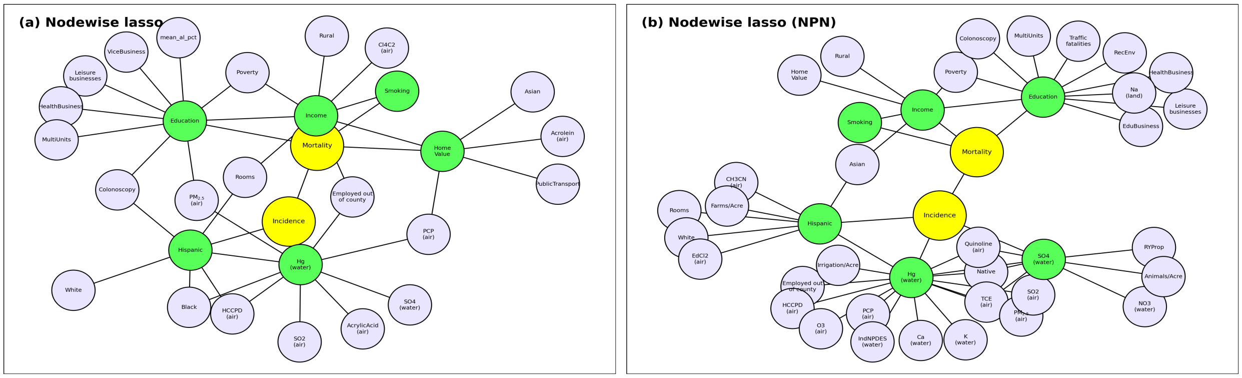

FIGURE S9. *County-level cancer results using nodewise lasso.* Estimated local graph of radius 2 using **(a)** original data and **(b)** nonparanormal data.

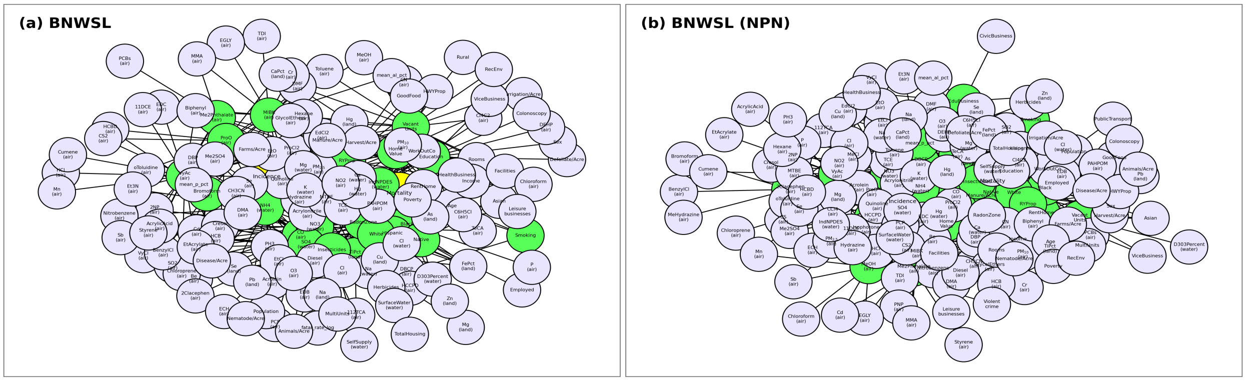

FIGURE S10. *County-level cancer results using BNWSL.* Estimated local graph of radius 2 using **(a)** original data and **(b)** nonparanormal data.

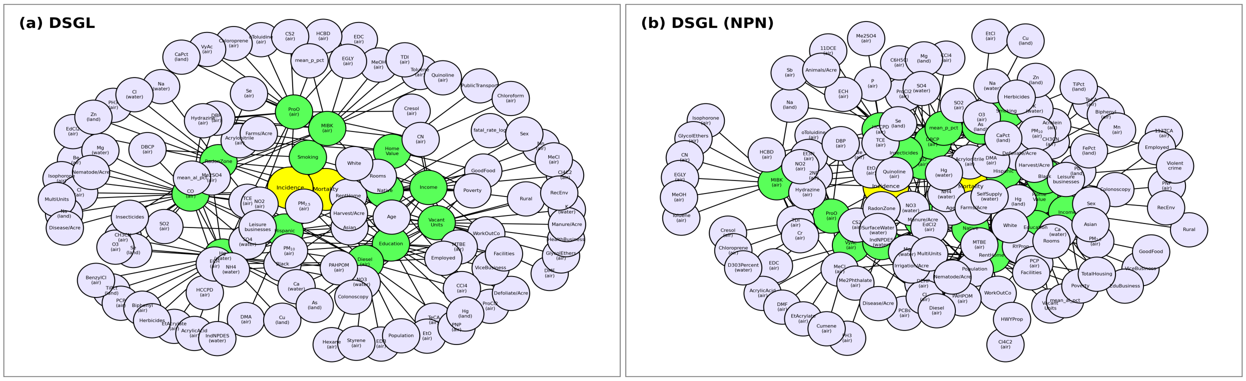

FIGURE S11. *County-level cancer results using DSGL.* Estimated local graph of radius 2 using **(a)** original data and **(b)** nonparanormal data.

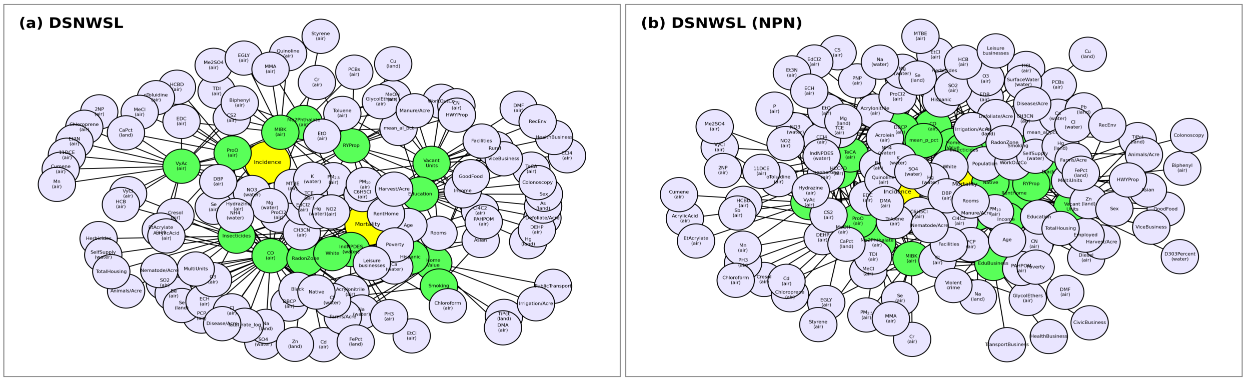

FIGURE S12. *County-level cancer results using DSNWSL.* Estimated local graph of radius 2 using **(a)** original data and **(b)** nonparanormal data.

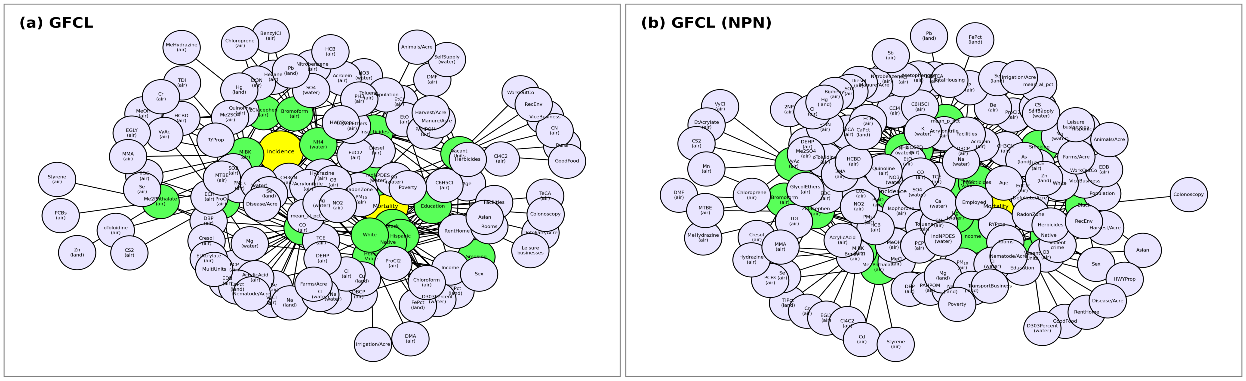

FIGURE S13. *County-level cancer results using GFCL.* Estimated local graph of radius 2 using **(a)** original data and **(b)** nonparanormal data.

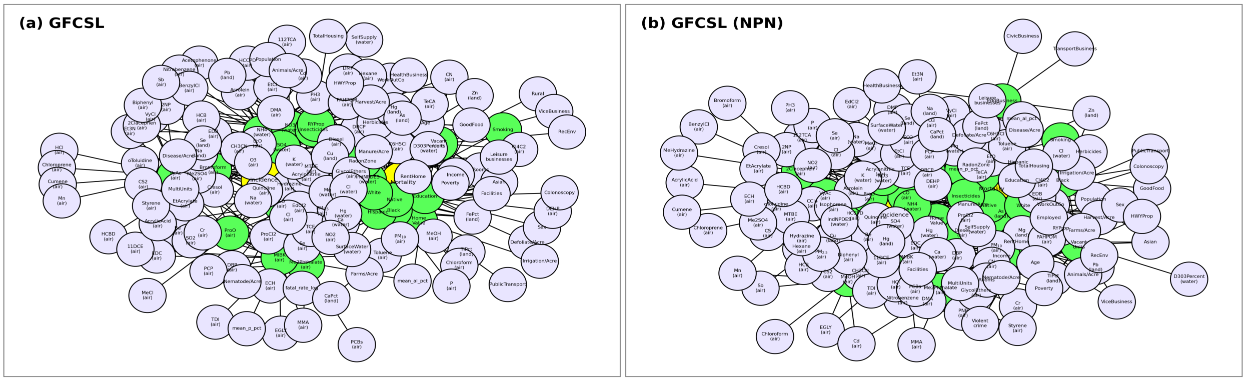

FIGURE S14. *County-level cancer results using GFCSL.* Estimated local graph of radius 2 using **(a)** original data and **(b)** nonparanormal data.

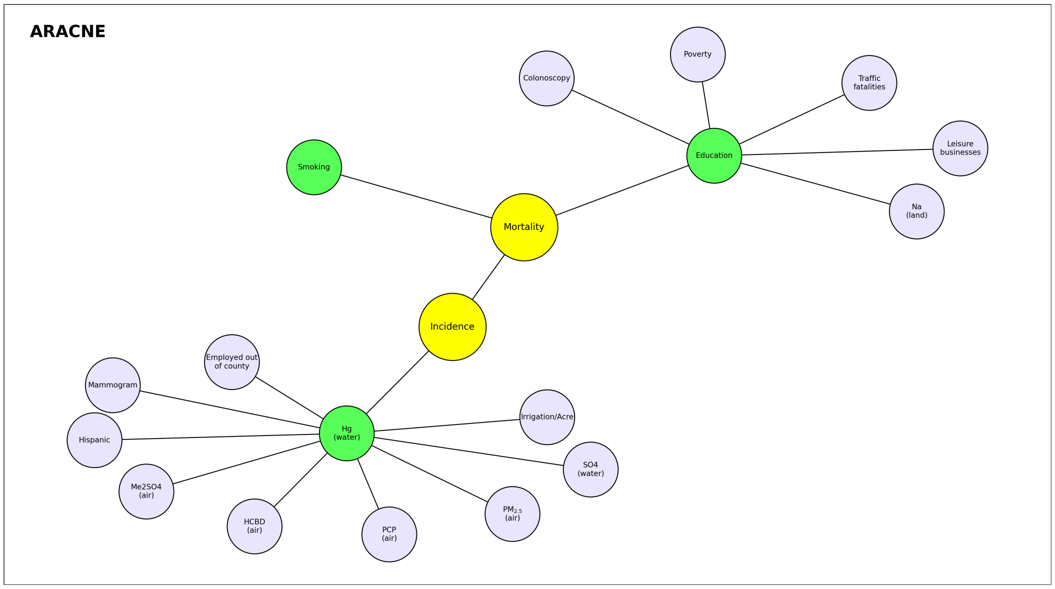

Figure S15. *County-level cancer results using ARACNE.* Estimated local graph of radius 2 using original data (applying the nonparanormal did not change results).

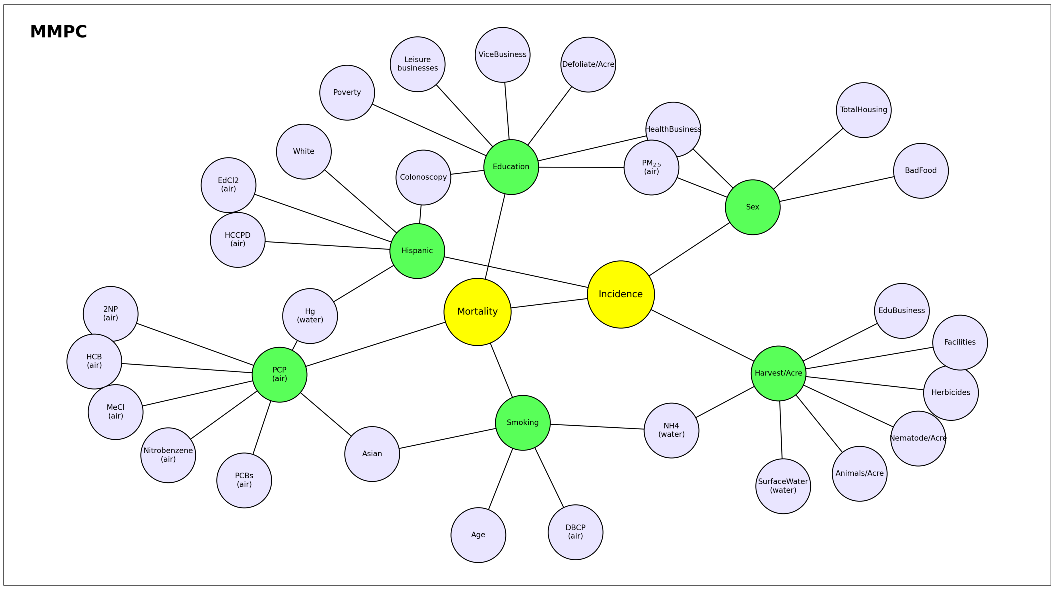

Figure S16. *County-level cancer results using MMPC.* Estimated local graph of radius 2 using original data (applying the nonparanormal did not change results).

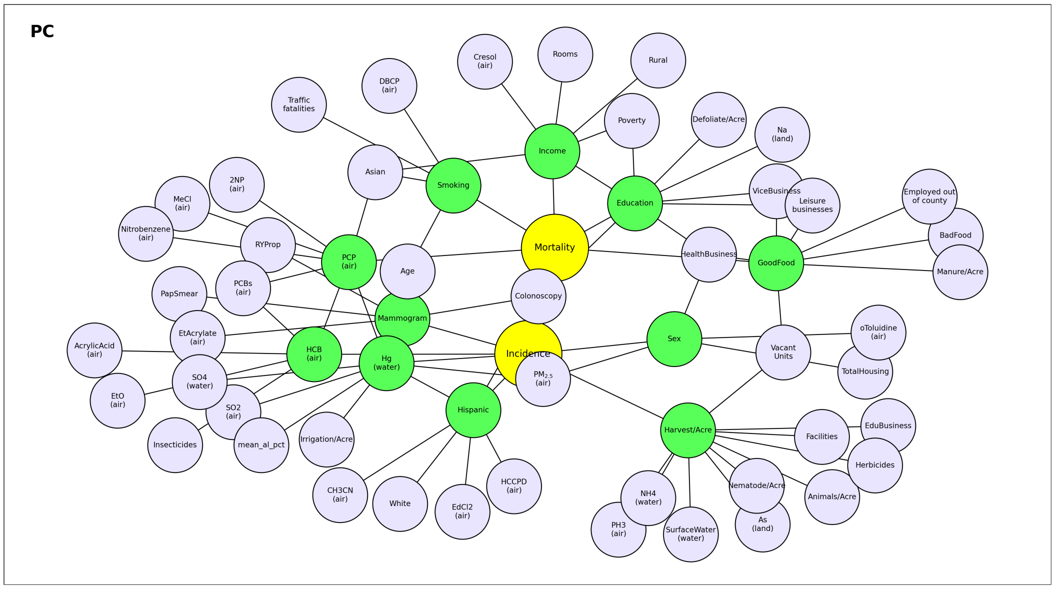

FIGURE S17. *County-level cancer results using StablePC*. Estimated local graph of radius 2 using original data (applying the nonparanormal did not change results).

S10.2. Breast cancer. For the breast cancer data ($n = 547$, $p = 10{,}744$), graphical lasso and nodewise lasso returned graphs in which all three of the target variables were isolated (no neighbors). Among the methods with global FDR control, only DSNWSL and GFCSL were computationally feasible (these ran in 4.5 and 5.25 hours, respectively; by contrast, PFS ran in 50 minutes on the same data). We ran these two methods at multiple FDR thresholds ranging from 0.01 to 0.5 (the `SILGGM` defaults are 0.05 and 0.1). Since the connection between pathologic stage and status has strong clinical support, results are reported for the smallest target FDR at which an edge is present between pathologic stage and status. GFCSL first detects the edge between pathologic stage and status at a target FDR of 0.1 for the original data, and 0.15 for the nonparanormal data. Figures S18a and S18b show that these target FDR values yield interpretable subgraphs of radius 1 around the target variables. However, the subgraphs of radius 2 (Figures S18c and S18d) are extremely dense and uninterpretable. DSNWSL does not detect the edge between pathologic stage and status for any target FDR at or below 0.5 (Figure S19a) on the original data, and detects it only at a target FDR of 0.5 for the nonparanormal data (Figure S19b). Among methods from the `bnlearn` package, ARACNE was the only approach that was computationally feasible for this dataset.

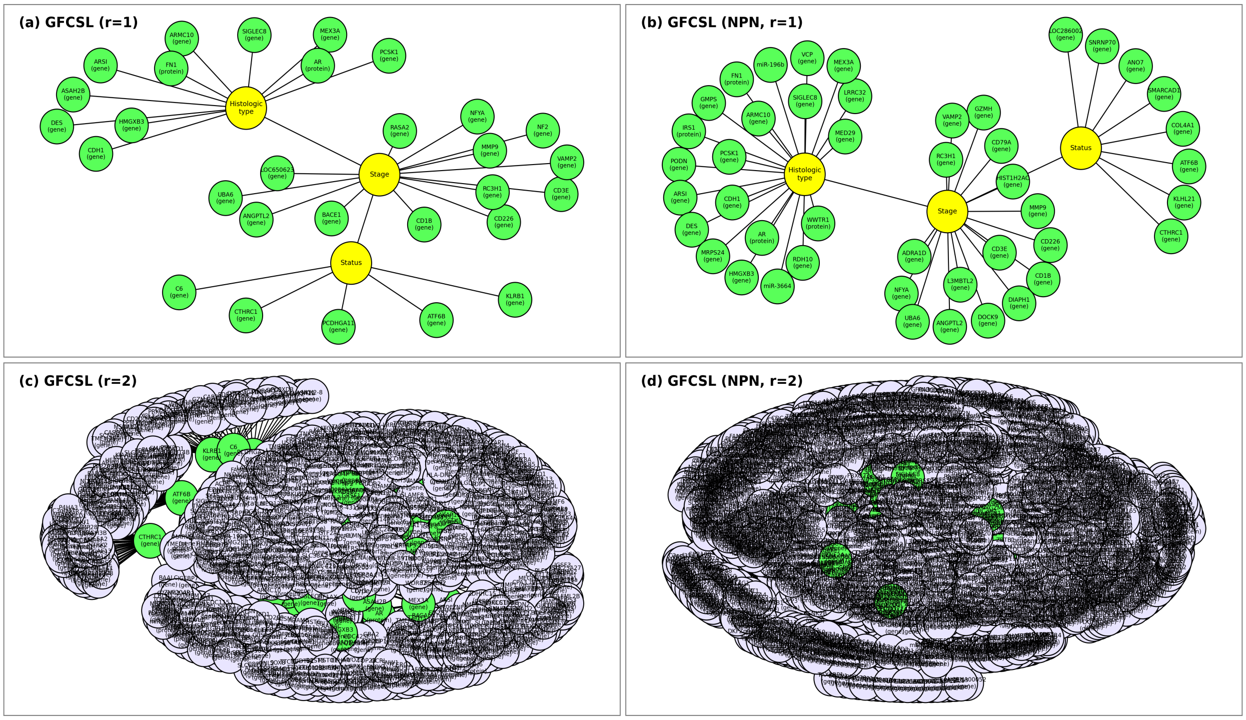

Figure S18. *Breast cancer results using GFCSL.* Estimated local graph of radius 1 using **(a)** original data and **(b)** nonparanormal data. Estimated local graph of radius 2 using **(c)** original data and **(d)** nonparanormal data.

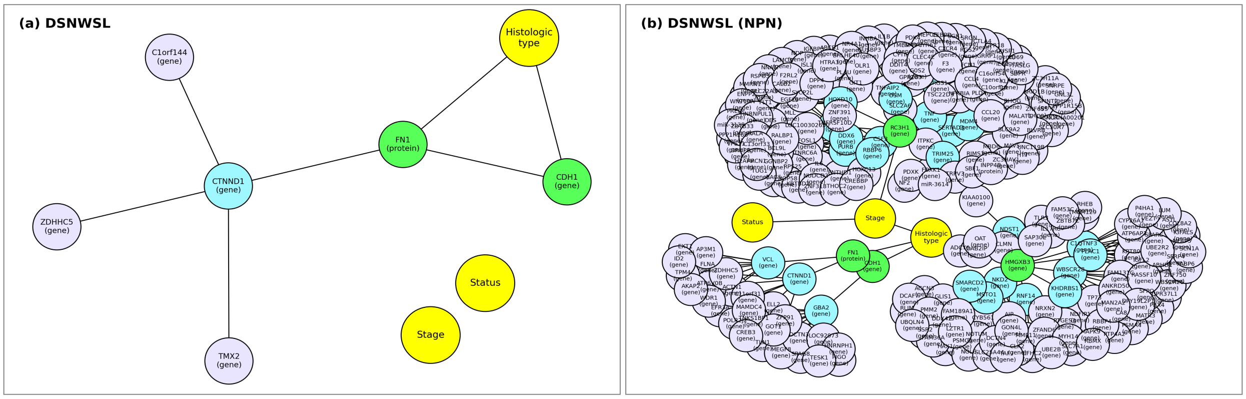

Figure S19. *Breast cancer results using DSNWSL.* Estimated local graph of radius 3 using **(a)** original data and **(b)** nonparanormal data.

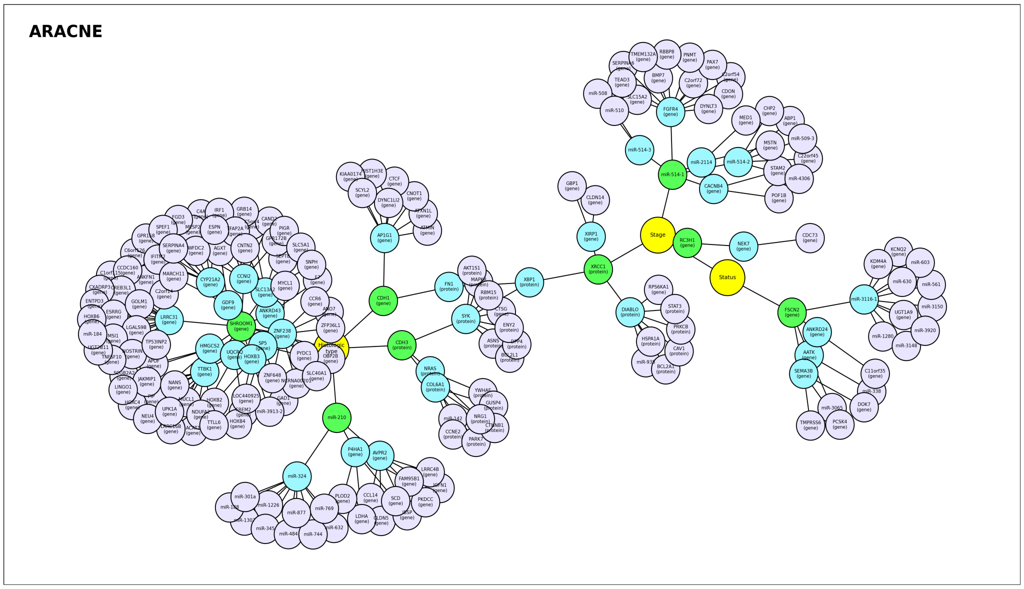

Figure S20. *Breast cancer results using ARACNE.* Estimated local graph of radius 3 using original data (applying the nonparanormal did not change results).

S10.3. Human connectome and cognition. In addition to PFS, we applied several other graph estimation methods to the HCP dataset to estimate local graphs of radius 3 around fluid cognition. The two regularization-based approaches, graphical lasso and GFCSL, were run with default settings. The stable PC algorithm was run with significance level $\alpha = 0.05$ using mutual information. For local neighborhood estimation, we applied nodewise versions of several constraint-based methods: HPC with $\alpha = 0.05$, IAMB with $\alpha = 0.005$, and MMPC with $\alpha = 0.025$. In each case, neighborhoods were estimated for the fluid cognition variable and expanded to radius 3. All conditional independence tests in these methods used mutual information.

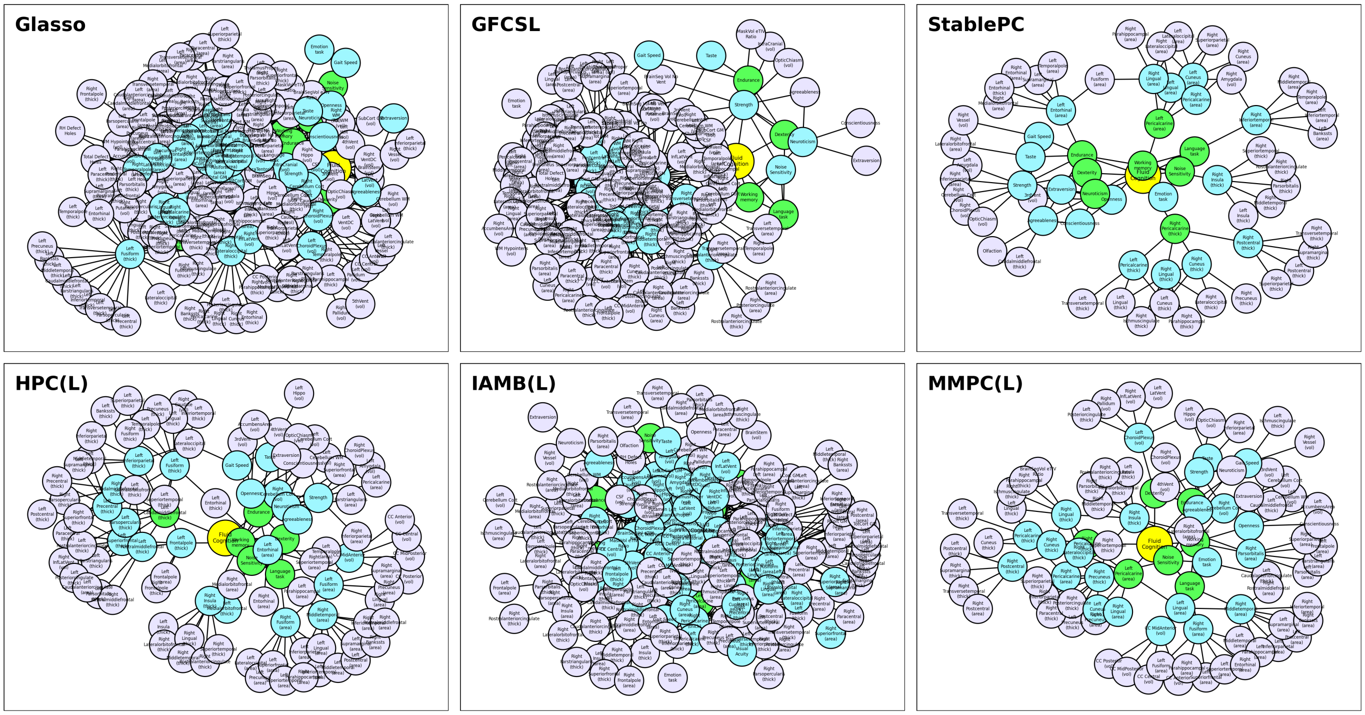

FIGURE S21. *Human Connectome Project (HCP) results using other methods.* Estimated local graphs of radius 3 around fluid cognition (yellow) using various graph estimation methods. Only methods that identified at least one edge between Fluid Cognition and a FreeSurfer-derived structural brain morphometry feature are shown.

Supplementary references

[1] Koller, D. & Friedman, N. *Probabilistic Graphical Models: Principles and Techniques* (MIT press, 2009).

[2] Friedman, J., Hastie, T. & Tibshirani, R. Sparse inverse covariance estimation with the graphical lasso. *Biostatistics* **9**, 432–441 (2008).

[3] Mazumder, R. & Hastie, T. Exact covariance thresholding into connected components for large-scale graphical lasso. *The Journal of Machine Learning Research* **13**, 781–794 (2012).

[4] Liu, H., Lafferty, J. & Wasserman, L. The nonparanormal: Semiparametric estimation of high dimensional undirected graphs. *Journal of Machine Learning Research* **10** (2009).

[5] Xue, L. & Zou, H. Regularized rank-based estimation of high-dimensional nonparanormal graphical models. *The Annals of Statistics* **40**, 2541–2571 (2012).

[6] Liu, W. Gaussian graphical model estimation with false discovery rate control. *The Annals of Statistics* **41**, 2948–2978 (2013).

[7] Zhang, R., Ren, Z. & Chen, W. SILGGM: An extensive R package for efficient statistical inference in large-scale gene networks. *PLoS Computational Biology* **14**, e1006369 (2018).

[8] Li, J. & Maathuis, M. H. GGM knockoff filter: False discovery rate control for Gaussian graphical models. *Journal of the Royal Statistical Society Series B: Statistical Methodology* **83**, 534–558 (2021).

[9] Drton, M. & Maathuis, M. H. Structure learning in graphical modeling. *Annual Review of Statistics and its Application* **4**, 365–393 (2017).

[10] Storey, J. D. The positive false discovery rate: A Bayesian interpretation and the q-value. *The Annals of Statistics* **31**, 2013–2035 (2003).

[11] Meinshausen, N. & Bühlmann, P. High-dimensional graphs and variable selection with the lasso. *The Annals of Statistics* **34**, 1436–1462 (2006).

[12] Melikechi, O., Dunson, D. B. & Miller, J. W. Nonparametric IPSS: fast, flexible feature selection with false discovery control. *Bioinformatics* **41**, btaf299 (2025).

[13] Janková, J. & van de Geer, S. Honest confidence regions and optimality in high-dimensional precision matrix estimation. *Test* **26**, 143–162 (2017).

[14] Scutari, M. Learning Bayesian networks with the bnlearn R package. *Journal of Statistical Software* **35**, 1–22 (2010).

[15] Zhao, T., Liu, H., Roeder, K., Lafferty, J. & Wasserman, L. The huge package for high-dimensional undirected graph estimation in R. *The Journal of Machine Learning Research* **13**, 1059–1062 (2012).

[16] Ren, Z., Sun, T., Zhang, C.-H. & Zhou, H. H. Asymptotic normality and optimalities in estimation of large Gaussian graphical models. *The Annals of Statistics* **43**, 991–1026 (2015).

[17] Jankova, J. & Van De Geer, S. Confidence intervals for high-dimensional inverse covariance estimation. *Electronic Journal of Statistics* **9**, 1205–1229 (2015).

[18] Margolin, A. A. *et al.* ARACNE: an algorithm for the reconstruction of gene regulatory networks in a mammalian cellular context. *BMC bioinformatics* **7**, S7 (2006).

[19] Gasse, M., Aussem, A. & Elghazel, H. A hybrid algorithm for Bayesian network structure learning with application to multi-label learning. *Expert Systems with Applications* **41**, 6755–6772 (2014).

[20] Tsamardinos, I., Aliferis, C. F., Statnikov, A. R. & Statnikov, E. Algorithms for large scale Markov blanket discovery. In *FLAIRS*, vol. 2, 376–81 (2003).

[21] Tsamardinos, I., Aliferis, C. F. & Statnikov, A. Time and sample efficient discovery of Markov blankets and direct causal relations. In *Proceedings of the ninth ACM SIGKDD international conference on Knowledge discovery and data mining*, 673–678 (2003).

[22] Aliferis, C. F., Statnikov, A., Tsamardinos, I., Mani, S. & Koutsoukos, X. D. Local causal and Markov blanket induction for causal discovery and feature selection for classification part I: algorithms and empirical evaluation. *Journal of Machine Learning Research* **11** (2010).

[23] Colombo, D., Maathuis, M. H. *et al.* Order-independent constraint-based causal structure learning. *Journal of Machine Learning Research* **15**, 3741–3782 (2014).

[24] Melikechi, O. & Miller, J. W. Integrated path stability selection. *Journal of the American Statistical Association* 1–11 (2025).

[25] Tibshirani, R. Regression shrinkage and selection via the lasso. *Journal of the Royal Statistical Society Series B: Statistical Methodology* **58**, 267–288 (1996).

[26] Chen, T. & Guestrin, C. XGBoost: A scalable tree boosting system. In *Proceedings of the 22nd ACM SIGKDD International Conference on Knowledge Discovery and Data Mining*, 785–794 (2016).

[27] Benjamini, Y. & Hochberg, Y. Controlling the false discovery rate: a practical and powerful approach to multiple testing. *Journal of the Royal Statistical Society: Series B* **57**, 289–300 (1995).

[28] Benjamini, Y. & Yekutieli, D. The control of the false discovery rate in multiple testing under dependency. *Annals of Statistics* 1165–1188 (2001).
[29] Candes, E., Fan, Y., Janson, L. & Lv, J. Panning for gold:model-xknockoffs for high dimensional controlled variable selection. *Journal of the Royal Statistical Society Series B: Statistical Methodology* **80**, 551–577 (2018).
[30] Seabold, S., Perktold, J. *et al.* Statsmodels: econometric and statistical modeling with Python. *scipy* **7**, 92–96 (2010).
[31] Messer, L. C., Jagai, J. S., Rappazzo, K. M. & Lobdell, D. T. Construction of an environmental quality index for public health research. *Environmental Health* **13**, 1–22 (2014).
[32] Wei, Y., Davis, J. & Bina, W. F. Ambient air pollution is associated with the increased incidence of breast cancer in us. *International Journal of Environmental Health Research* **22**, 12–21 (2012).
[33] Wang, N. *et al.* Lung cancer and particulate pollution: A critical review of spatial and temporal analysis evidence. *Environmental Research* **164**, 585–596 (2018).
[34] Kentros, P. A. *et al.* Ambient particulate matter air pollution exposure and ovarian cancer incidence in the usa: an ecological study. *BJOG: An International Journal of Obstetrics & Gynaecology* **131**, 690–698 (2024).
[35] Vasaikar, S. V., Straub, P., Wang, J. & Zhang, B. Linkedomics: analyzing multi-omics data within and across 32 cancer types. *Nucleic Acids Research* **46**, D956–D963 (2018).
[36] Liberzon, A. *et al.* Molecular signatures database (MSigDB) 3.0. *Bioinformatics* **27**, 1739–1740 (2011).
[37] Fang, Z., Liu, X. & Peltz, G. GSEApy: a comprehensive package for performing gene set enrichment analysis in Python. *Bioinformatics* **39**, btac757 (2023).
[38] Szklarczyk, D. *et al.* The STRING database in 2025: protein networks with directionality of regulation. *Nucleic Acids Research* **53**, D730–D737 (2025).
[39] Van Essen, David C and Smith, Stephen M and Barch, Deanna M and Behrens, Timothy EJ and Yacoub, Essa and Ugurbil, Kamil and Wu-Minn HCP Consortium and others. The WU-Minn human connectome project: an overview. *Neuroimage* **80**, 62–79 (2013).
[40] Fischl, B. FreeSurfer. *Neuroimage* **62**, 774–781 (2012).
[41] Yeo, B. T. *et al.* The organization of the human cerebral cortex estimated by intrinsic functional connectivity. *Journal of Neurophysiology* **106**, 1125–1165 (2011).
[42] Desikan, R. S. *et al.* An automated labeling system for subdividing the human cerebral cortex on MRI scans into gyral based regions of interest. *Neuroimage* **31**, 968–980 (2006).
[43] Alexander-Bloch, A. F. *et al.* On testing for spatial correspondence between maps of human brain structure and function. *Neuroimage* **178**, 540–551 (2018).
[44] Grubman, Alexandra and Chew, Gabriel and Ouyang, John F and Sun, Guizhi and Choo, Xin Yi and McLean, Catriona and Simmons, Rebecca K and Buckberry, Sam and Vargas-Landin, Dulce B and Poppe, Daniel and others. A single-cell atlas of entorhinal cortex from individuals with Alzheimer's disease reveals cell-type-specific gene expression regulation. *Nature Neuroscience* **22**, 2087–2097 (2019).
