# Supplementary material for: Local graph estimation with pathwise false discovery control
Source: Nat Commun. 2026 May 12;17:6353. doi: 10.1038/s41467-026-72796-9 (PMC13376902; doi:10.1038/s41467-026-72796-9)
Supplement: Supplementary file 2 — Transparent Peer Review file [file 41467_2026_72796_MOESM2_ESM.pdf]

nature portfolio

Peer Review File

Local graph estimation with pathwise false discovery control

Corresponding Author: Dr Omar Melikechi

This file contains all reviewer reports in order by version, followed by all author rebuttals in order by version.

Version 2:

Reviewer comments:

Reviewer #1

(Remarks to the Author)
The authors introduce a local estimation framework for conditional independence graphs and a novel method, pathwise feature selection (PFS), which provides false discovery control along paths.

I consider local graph estimation an important problem and their method technically sound and potentially very useful.

Its advantages over previous, full-graph estimation methods (global and nodewise) are convincingly justified in theory (Supplement S2) and demonstrated in simulation studies (S6). The results on real data in the main manuscript also seem plausible.

The manuscript is well-written and I have only a few minor suggestions for its improvement:

* The authors should expand on the statement "In Theorem S3.2, we prove that the sum of q-values along an estimated path upper bounds the probability that the path is not in the true graph G.".
Does this guarantee only hold for any single path in isolation, or jointly for a set of (possibly overlapping) paths? What are the key assumptions, and what could go wrong if they were violated? Can the results be interpreted from a Bayesian perspective?
As far as I can assess, these questions have been rigorously addressed in the Supplement, but a less technical distillation would be helpful in the main text.

* The key findings from the simulation studies (now in the supplement) should be briefly summarised and/or discussed in the main text. In particular, I find it relevant that PFS achieves a better trade-off between true positive rates and false discovery rates than traditional full-graph estimators. Is there a theoretical underpinning for this?

* That the graphs in question are "conditional independence graphs" should be mentioned earlier in the text, either in the abstract or introduction.

(Remarks on code availability)

Reviewer #2

(Remarks to the Author)
The authors present a new method useful for local graph estimation, PFS (pathwise feature selection). This method allows for the inference of local subgraphs that identify significant links between target variables and other variables in the observed system, without the need to estimate the entire graph. Traditional methods rely on the latter approach, which is computationally expensive and often leads to dense networks, where the explosion in the number of spurious links obscures the signal of interest. The major strengths of PFS lie in the control of the false discovery rate associated with the inferred individual edges, the possibility of including mixed variable types, and the ability to recognize nonlinear interactions between variables.
I find the idea interesting, innovative in the field of feature selection, and formally sound. Nevertheless, I think it is necessary to improve the validation of the results by making it more quantitative, especially if the goal of the work is to propose and distribute a Python package that can be used by third parties to conduct research in various fields of application. Below are

some points that could help to make PFS more transparent, interpretable, and reliable.

A - Quantitative Validation and Interpretation:
1) The results are validated only qualitatively, arguing that the observed links in the inferred local graphs more or less make sense given the existing literature. Is there any metric that can quantify the performance in terms of accuracy/precision/recall of the estimated links?
For example, in the section ‘Uncovering cross-modal pathways in breast cancer,’ the paths between target variables and individual genes/proteins/micro-RNAs are sometimes justified one by one, which is misleading when considering how Gene Ontology (GO) taxonomies are constructed. A single gene/protein/micro-RNA can belong to many different GOs, and if we take in consideration a dataset of cancer it is expected that the great majority of the genes sampled in the dataset have a direct connection with cancer in the literature. In other words, justifying the presence of a gene/protein/micro-RNA by referring to a single one of its (many) GOs easily leads to a confirmation bias. Instead, it would be interesting to conduct a statistically significant validation based on an enrichment/over-representation analysis of groups of genes/proteins/micro-RNAs in the various classes defined by the target variables (histologic type, stage, status), to show that the features connected to them in the subgraph are indeed relevant for such classification.

B - Comparative Analysis and Performance Metrics:
1) Holding the target variable constant, what is the relationship between a local graph estimated with PFS and those derived from full graph estimation? Are topological observables conserved when transitioning from one to the other? For instance, how do the centrality measures of the target variables vary?
2) Is there a relationship between sub-networks identified by network clustering algorithms like MAPPR (Local Higher-Order Graph Clustering, 2017, doi: 10.1145/3097983.3098069) and PFS?
3) How does the performance of PFS vary as a function of the ratio between the number of samples and the number of features?
4) The choice of r_{max} and q*_{threshold} is not particularly argued. Can you provide some references that suggest specific ranges to try for these two quantities?

(Remarks on code availability)

Reviewer #3

(Remarks to the Author)
The article is coherent and well written. The presented research is sound and deserves wider attention. The manuscript articulates limitations of some of the existing methods, proposes a coherent solution and validates its performance with both an artificial simulation and two real datasets that yield potentially significant biological findings. The manuscript may benefit from the feedback bellow.

1. I suggest expanding the introduction and describing motivation in a bit more detail. The claim that existing methods often yield dense graphs is not supported very well as the only methods considered are variants of LASSO. Surely, not ALL existing methods yield equally dense graphs. If LASSO does have this undesirable tendency, can the problem be traced to poor regularization or is it inherent in the formulation?

2. It seems there are two independent goals that are not articulated very well. The advantage of targeted estimation lies with computational efficiency for large datasets and does not directly address density or accuracy. Even for denser structures obtained via global estimation, given the Local Markov property, it's always possible to extract just the Markov Neighborhood (MN) of a target variable, simplifying interpretation significantly (unless MN itself happens to contain too many nodes). On the other hand, the improved accuracy of PFS is what addressed density and, as such, perhaps deserves a separate paragraph. Can this improvement in accuracy be extended to the global graph estimation?

3. Figure 1e obscures the MN of the target variables, so it's difficult to directly assess how much improvement PFS actually delivers. Sometimes, a few unfortunate edges will fold the graphs on itself and make it impossible to read. There is no way to tell whether something like this may be going on here. The MN here seems to be comprised of roughly the same nodes as that of Figure 2, so it's not clear whether the improvement to the MN itself is significant.
The same applies to most of the figures in the supplementary material (except for Figure S8). It would help if, at least, the dissimilarity between MNs of different graphs, e.g. the count of the same edges, were assessed somewhere in the manuscript.

2. Local graph estimation (estimation of local structures around a group of target variables) is not exactly a new problem. It has been considered in the form of local MN estimation at least for directed graphs. Local MN estimation has been used as a way of dealing with performance limitations in a wide set of applications, including a variety of biological applications like GWAS studies. It would be advisable to include some discussion regarding what could be considered prior/similar work.
It would be helpful if the manuscript considered a wider context. For example, does PFS offer something fundamentally different with respect to other local estimation methods? Can it be compared to non-LASSO techniques? In what way would estimating information-theoretic quantities like Mutual Information over the same extended structures be inferior to feature selection via PFS?

3. Figure 2 could use more discussion. It reveals what could be at least some degree of "overfitting" and yet this is what PFS is supposed to control. For example 5 nodes (Mortality, Education, Poverty, Home Value, Income) form a completely connected graph where at least a few nodes are likely conditionally independent, e.g. Mortality and HomeValue given Income, or Education and Income given Poverty. Another fully connected structure (Incidence, PM2.5, TCE, SO2) appears on the left side of the figure. There are some questionable fully connected node triples scattered around the figure that further contribute to the overall density. Is the sensitivity too high? Do the user specified parameters need further adjustment? How can this be interpreted in terms of the simulation studies in S6.2? How stable are these findings under varying parameters?

4. As a core component, IPSS should be at least briefly formulated somewhere in the manuscript. What appears in the supplementary material is not quite sufficient and IPSS is treated as a black-box. This is not a well enough known method, particularly the variant used in this paper, to be treated so casually.

(Remarks on code availability)
The installation instructions could be improved if step-by-step instructions, starting from a clean python installation to the test-run, were added. I was able to pip-install the code, but, unfortunately, not all package dependencies were resolved. Attempting to execute simulation.py and run_pfs.py revealed missing packages -- pandas, pyqt5, rpy2, etc... It might be helpful to list the whole dependency tree on the main page (including a working installation of R interfaced with python).

The datasets are as described in the manuscript.

Reviewer #4

(Remarks to the Author)
This study introduces Pathwise Feature Selection (PFS), a computational method for local graph estimation—focused on inferring sparse and interpretable subgraphs around target variables from high-dimensional data. PFS is built on conditional independence graphs, using integrated path stability selection (IPSS) for nonparametric q-value estimation. It iteratively expands neighborhoods from target variables and tracks cumulative path uncertainty via summed q-values. Unlike traditional full-graph methods that yield dense, uninterpretable results, PFS recovers sparse, biologically meaningful local subgraphs, handles nonlinear dependencies and mixed variable types, and scales to high-dimensional data. While PFS has been validated in two cancer studies, providing a targeted, interpretable approach for local graph inference, I have a few questions to be addressed that are listed below.

Major concerns:
1. The authors claim existing graph estimation methods yield dense, less interpretable graphs prone to false discoveries, so PFS uses a user-specified radius to constrain output to the target variable’s radius-hop neighborhood, ensuring sparsity for interpretability and false positive control. However, real-world local graphs around targets (e.g., in cancer onset or gene regulation) may not be sparse—multiple relevant factors could exist, and forced sparsity may cause false negatives. Key questions remain unaddressed: How does PFS balance its emphasis on sparsity and false positive control while minimizing false negatives? Moreover, in the simulated test, the ground-truth local graph around the target is inherently sparse, potentially masking false negative issues; can PFS still effectively resolve local structure when the ground-truth around the target is dense?

2. The introduction outlines the limitations of existing high-dimensional data analysis methods, such as the inability to handle all variables simultaneously, lack of interpretability, and restrictive assumptions. However, it remains unclear how the authors' chosen framework—graphical estimation—addresses these issues. A more compelling justification is needed. Further comparative testing is needed to evaluate the performance of the graph estimation framework against other frameworks (such as deep learning and machine learning based frameworks) in terms of interpretability, computational

efficiency, and preservation of genuine signals.

3. The core PFS methodology relies on IPSS q-values to determine edges and control false discoveries, yet the advantages of using IPSS q-values in graph estimation scenarios compared to other statistical measures remain unclarified.

4. When conducting benchmarking, the authors only compared PFS with existing graph estimation-based methods. However, given that numerous other methods have been developed for multi-omics studies and high-dimensional data analysis, for example, by Uyar et al.(https://pubmed.ncbi.nlm.nih.gov/40940333/) and by Argelaguet et al. (https://pubmed.ncbi.nlm.nih.gov/32393329/), the performance of PFS should also be evaluated against these alternative approaches on the same datasets.

5. The method requires users to set a pathwise q-value threshold. The article mentions using a value of 0.4 for this threshold - what is the justification for selecting this specific value? A method should be provided to evaluate the performance of different pathwise q-value thresholds.
6. In the discussion section, the authors mention that PFS demonstrates high generalizability; however, its current validation has only been conducted on public health datasets and bulk multi-omics datasets, which appears insufficient. The authors should extend the testing of PFS to more heterogeneous datasets, such as single-cell and spatial multi-omics datasets.

Minor concerns:
1. A Methods section should be added to the main text to outline the key steps and rationale of the proposed algorithm.

2. Figure 1c and 1d are not cited or described in the main text.

3. Given that Figure 1e and Figure 2 present results from different methods applied to the same dataset, I suggest consolidating them into a new, combined Figure 2.

4. The process for generating the simulated data used in Figure 1 should be described in the Methods section.

(Remarks on code availability)
The software package can be installed.

Version 3:

Reviewer comments:

Reviewer #1

(Remarks to the Author)
The authors have addressed all concerns to my satisfaction.

(Remarks on code availability)

Reviewer #2

(Remarks to the Author)
The authors have comprehensively addressed all my previous concerns. Rather than simply providing textual justifications, they have conducted substantial new analyses that significantly elevate the methodological and empirical solidity of the paper.
I am particularly satisfied with the following revisions:

Quantitative Validation: The inclusion of formal Over-Representation Analyses (ORA) for the real-world datasets, alongside the empirical validation using the STRING database (Tables S9 and S10), completely resolves my initial concern regarding potential confirmation bias. Moving from a qualitative "gene-by-gene" narrative to a statistically sound module-based evaluation greatly enhances the biological interpretability and reliability of the method.

Performance Metrics and Sample Size: The expanded simulation studies explicitly reporting TPR and FDR across different network topologies (dense/sparse, linear/non-linear) provide the quantitative transparency I requested. Furthermore, the new analysis evaluating the performance as a function of the sample-to-feature ratio (Figure S2) effectively demonstrates the algorithm's consistency and false discovery control across different dimensional regimes.

While the selection of parameters such as r_{max} and q^* relies on theoretical thresholds and user-defined tolerance rather than offering an automated data-driven heuristic, I acknowledge that this is a standard constraint in this class of statistical frameworks and accept their rationale.
Overall, the authors have done an excellent job refining their work. I have no further critiques and recommend the manuscript for publication in Nature Communications.

(Remarks on code availability)

Reviewer #3

(Remarks to the Author)
I thank the authors for addressing almost all of the original concerns.
A few remaining outstanding issues remain:

1. The first paragraph on page 2:
Now the text seems to suggest that the second class of approaches (including information-theoretic methods) scales poorly. This is, of course, not the case and should be reworded. First of all, not all methods from the second class are the same, and this is quite evident in Table S1 of the supplement (ARACNE is actually almost always faster than PFS). So, only some methods scale poorly, which must also be highly implementation-dependent. Second, methods requiring matrix operations will generally scale worse than scoring/testing methods by design. Any method that needs LAPACK solvers for searching the solution space will usually be more computationally demanding and have greater scaling limitations compared to some serial alternatives.

2. Second paragraph on page 2:
Prior work in local graphs estimation needs more attention. Even though the local MN estimation is often restricted to the immediate neighborhood, the extension of ANY local procedure to the estimation of the optimal embedding envelope of arbitrary size is a natural next step that has been taken before more than once (e.g. Gao (2017) Local-to-Global BN Structure Learning, etc.). So, what this paragraph seems to claim, due to its unrestricted generality, is simply not true. If the listed limitations are attributable to specific packages (e.g. bnlearn) then the claims about existing MN estimation strategies should be restricted to these specific methods/packages to avoid making overly general claims.

3. Page 5 last paragraph:
The claim that PFS achieves the "highest TPR among all methods in both linear and..." contradicts the tables in the supplement where some methods seem to outperform PFS in power for linear setting (G/NLASSO, ARACNE, HPC, etc. in Table S2; G/NLASSO in Table S3; NLASSO, BNWSL, GFCSL, etc. in Table 4). Where PFS does outperform other methods in power is specifically the nonlinear setting, which should really be brought out from the start. Perhaps, a robust balance between TPR and FDR across other scenarios, but certainly not "the highest TPR". If I may suggest author's own words from the rebuttal item 3.5: "this work suggests that PFS provides a favorable balance between error control and discovery".

(Remarks on code availability)
I was able to install the code and run a few scripts (e.g. run_pfs.py).
But unfortunately for some scripts either the paths are garbled or something else

FileNotFoundError: [Errno 2] No such file or directory: '../data/cleaned_data/cleaned_data.csv'
ImportError: cannot import name 'max_cor_response' from 'localgraph'

I did not have the time to troubleshoot this and could not reproduce the results.
Also, the "results" folder described in README is missing and is never created.

Reviewer #4

(Remarks to the Author)
The manuscript has been carefully revised to clarify the scope and contributions of the work. The inclusion of additional simulation studies and a new single-nucleus RNA-sequencing application strengthens the empirical validation. However, two specific concerns remain regarding the methodological evaluation and data presentation.

Major Concern :
An ablation study is necessary to empirically validate the proposed algorithmic combination. Specifically, the authors should keep the PFS framework constant while substituting the underlying q-value generator to evaluate the empirical performance of IPSS q-values specifically within the PFS framework.
While the authors provided a theoretical explanation of the advantages of IPSS q-values—such as their nonparametric

nature and explicit false discovery control—the core of the original concern remains unaddressed. The cited IPSS literature evaluates its performance strictly in single-target feature selection scenarios. In contrast, PFS relies on an iterative, pathwise expansion where uncertainties are summed along multi-hop network paths. It cannot be assumed that a metric optimized for single-step selection remains optimal under cumulative pathwise error propagation.

Minor Concern :
Figure 1e and Figure 2 should be consolidated into a new Figure 2. Figures 1a-d, based on simulated data, sufficiently establish the research motivation by illustrating the differences between global graph estimation methods and PFS. Since Figure 1e and Figure 2 visualize the same real-world dataset, combining them offers a direct side-by-side comparison that highlights the practical advantages of PFS in real-world scenarios and enhances readability without disrupting the logical flow.

(Remarks on code availability)

Version 4:

Reviewer comments:

Reviewer #3

(Remarks to the Author)
I thank the authors for addressing the concerns and introducing appropriate changes into the manuscript.

(Remarks on code availability)
The code repository is now missing the "requrements.txt" file, so there is no way to install it according to the repository instructions. I would advise that the installation section on the intro page should precede the "quick start" section.

Reviewer #4

(Remarks to the Author)
The authors have addressed all my comments, and I have no further concerns

(Remarks on code availability)

Open Access This Peer Review File is licensed under a Creative Commons Attribution 4.0 International License, which permits use, sharing, adaptation, distribution and reproduction in any medium or format, as long as you give appropriate credit to the original author(s) and the source, provide a link to the Creative Commons license, and indicate if changes were made.
In cases where reviewers are anonymous, credit should be given to 'Anonymous Referee' and the source.

The images or other third party material in this Peer Review File are included in the article's Creative Commons license, unless indicated otherwise in a credit line to the material. If material is not included in the article's Creative Commons license and your intended use is not permitted by statutory regulation or exceeds the permitted use, you will need to obtain permission directly from the copyright holder.
To view a copy of this license, visit https://creativecommons.org/licenses/by/4.0/

Reply to reviews for "Local graph estimation: Interpretable network discovery for complex data"

We thank the Associate Editor and the reviewers for their careful and constructive evaluation of our manuscript and for extending us the opportunity to revise it. We have substantially updated the manuscript to address each concern; for ease of reference, all changes in the main text and Supplement are shown in blue. Key updates include:

- A substantially revised Introduction clarifying the scope and contributions of the work
- Multiple new simulation studies
- Comparisons of PFS to 14 other methods, including 7 newly added ones
- Two new applications: an analysis of the human connectome and cognition, and a single-nucleus RNA-sequencing study of Alzheimer's disease
- A Methods section, including expanded discussions of Algorithm 1 (PFS) and theoretical results
- Multiple new over-representation analyses that support our empirical findings

Below, we provide a detailed, point-by-point response to each comment.

Reviewer 1

The authors introduce a local estimation framework for conditional independence graphs and a novel method, pathwise feature selection (PFS), which provides false discovery control along paths.

I consider local graph estimation an important problem and their method technically sound and potentially very useful.

Its advantages over previous, full-graph estimation methods (global and nodewise) are convincingly justified in theory (Supplement S2) and demonstrated in simulation studies (S6). The results on real data in the main manuscript also seem plausible.

The manuscript is well-written and I have only a few minor suggestions for its improvement:

1.1. The authors should expand on the statement "In Theorem S3.2, we prove that the sum of q-values along an estimated path upper bounds the probability that the path is not in the true graph G.". Does this guarantee only hold for any single path in isolation, or jointly for a set of (possibly overlapping) paths? What are the key assumptions, and what could go wrong if they were violated? Can the results be interpreted from a Bayesian perspective? As far as I can

assess, these questions have been rigorously addressed in the Supplement, but a less technical distillation would be helpful in the main text.

We have added a new discussion of Theorem S3.2 to the Methods section. This discussion clarifies that the guarantee is path-specific rather than joint over collections of paths, describes the key assumptions underlying the result, explains what could go wrong if these assumptions are violated, and outlines the associated Bayesian interpretation. The presentation is nontechnical and distills the theoretical results from the Supplement into the main text, as requested.

1.2. The key findings from the simulation studies (now in the supplement) should be briefly summarised and/or discussed in the main text. In particular, I find it relevant that PFS achieves a better trade-off between true positive rates and false discovery rates than traditional full-graph estimators. Is there a theoretical underpinning for this?

We have added a new section to the main text, Section 3, summarizing and discussing the simulation results. We now highlight that pathwise false discovery control is the theoretical underpinning for the favorable performance of PFS relative to other methods. Specifically, in the second paragraph of Section 3 we state: "*PFS achieves a favorable balance between TPR and FDR across all experiments. One reason for this is that PFS is the only method with rigorous pathwise false discovery control, which enables precise inference at targets while preventing errors from propagating away from them.*"

1.3. That the graphs in question are "conditional independence graphs" should be mentioned earlier in the text, either in the abstract or introduction.

Thank you for this suggestion. We now mention that this work focuses on conditional independence graphs when graph estimation is first introduced in the Introduction.

Reviewer 2

The authors present a new method useful for local graph estimation, PFS (pathwise feature selection). This method allows for the inference of local subgraphs that identify significant links between target variables and other variables in the observed system, without the need to estimate the entire graph. Traditional methods rely on the latter approach, which is computationally expensive and often leads to dense networks, where the explosion in the number of spurious links obscures the signal of interest. The major strengths of PFS lie in the control of the false discovery rate associated with the inferred individual edges, the possibility of including mixed variable types, and the ability to recognize nonlinear interactions between variables. I find the idea interesting, innovative in the field of feature selection, and formally sound. Nevertheless, I think it is necessary to improve the validation of the results by making it more quantitative, especially if the goal of the work is to propose and distribute a Python package that can be used by third parties to conduct research in various fields of application. Below are some points that could help to make PFS more transparent, interpretable, and reliable.

Quantitative Validation and Interpretation:

2.1. The results are validated only qualitatively, arguing that the observed links in the inferred local graphs more or less make sense given the existing literature. Is there any metric that can quantify the performance in terms of accuracy/precision/recall of the estimated links?

For example, in the section "*Uncovering cross-modal pathways in breast cancer*," the paths between target variables and individual genes/proteins/micro-RNAs are sometimes justified one by one, which is misleading when considering how Gene Ontology (GO) taxonomies are constructed. A single gene/protein/micro-RNA can belong to many different GOs, and if we take in consideration a dataset of cancer it is expected that the great majority of the genes sampled in the dataset have a direct connection with cancer in the literature. In other words, justifying the presence of a gene/protein/micro-RNA by referring to a single one of its (many) GOs easily leads to a confirmation bias. Instead, it would be interesting to conduct a statistically significant validation based on an enrichment/over-representation analysis of groups of genes/proteins/micro-RNAs in the various classes defined by the target variables (histologic type, stage, status), to show that the features connected to them in the subgraph are indeed relevant for such classification.

Thank you for this suggestion, which we believe substantially improves the paper.

Regarding quantitative performance metrics, measures such as accuracy, precision, and recall require knowledge of the true underlying graph, which is generally only available in simulated settings. To this end, we have expanded our simulation studies (Sections 3 and S5), adding multiple new scenarios and additional competing methods. In each study, we report true positive rates (recall) and false discovery rates ($1 -$ precision) at different radii.

We agree that assessing variables individually or pairwise risks confirmation bias. Thus, following your suggestion, we have added multiple over-representation analyses (ORAs) to the manuscript to provide statistically significant, set-level validation. ORAs spanning multiple domains and modalities are included for the breast cancer study as well as for the newly added human connectome and Alzheimer's disease applications. Across these studies, pathways and clusters of variables in the local graphs estimated by PFS show significant enrichment for pathway and functional categories consistent with domain knowledge and the associated targets; see, in particular, Tables 1 and 2 in the main text and Tables S9 and S10 in the supplement.

Comparative Analysis and Performance Metrics:

2.2. Holding the target variable constant, what is the relationship between a local graph estimated with PFS and those derived from full graph estimation? Are topological observables conserved when transitioning from one to the other? For instance, how do the centrality measures of the target variables vary?

If the full graph is perfectly recovered, then the local graph around a fixed target variable is also recovered. Thus, a local graph estimated with PFS and a local graph derived from a full graph estimate coincide when both are correct. The distinction lies in how the problem is approached: PFS estimates the local graph directly, whereas full graph estimation recovers local graphs only as a byproduct of a global estimate. Section S2 provides theoretical explanations for why the latter approach can fail to recover local structure, while simulation and application results reported throughout the main text and supplement provide empirical evidence of this behavior.

Centrality measures are designed to quantify the global importance of vertices within a network. In the context of local graph estimation, however, the notion of importance is defined a priori by the scientific question via the choice of target variables. As a result, centrality is not an especially relevant diagnostic in the local setting: vertices with high global degree or other centrality scores may be unrelated to the targets, while vertices that are weakly connected

globally may be highly relevant locally. Consequently, centrality measures do not align with the inferential goals of PFS and are not used to assess the estimated local graphs.

2.3. Is there a relationship between sub-networks identified by network clustering algorithms like MAPPR (Local Higher-Order Graph Clustering, 2017, doi: 10.1145/3097983.3098069) and PFS?

The relationship between PFS and network clustering algorithms such as MAPPR is that the former aims to estimate graph structure, whereas the latter assumes the graph is known and uses it to identify clusters of nodes. In this sense, local graph estimation and network clustering are complementary, with clustering naturally operating downstream of graph estimation; we now note this in the Discussion and cite MAPPR as an example.

2.4. How does the performance of PFS vary as a function of the ratio between the number of samples and the number of features?

In general, the performance of PFS, as well as of other methods, improves as the number of samples grows relative to the number of features. To address this comment, we have added evaluations of the true positive rates (TPRs) and false discovery rates (FDRs) of PFS at radii 1, 2, 3, and 4 in the sparse, linear setting as n ranges over $\{50, 100, 150, 200, 250, 300, 350, 400\}$ and the number of features is fixed at $p = 200$. The results, illustrated in Figure S2, show that FDRs remain well-controlled across all sample sizes while TPRs increase steadily with n. A similar pattern appears in Tables S4 and S5, which report simulation results for otherwise identical settings when $n = 100$ and $n = 500$, respectively (and $p = 200$).

The relationship between n, p, and PFS can also be seen in its q-values: generally speaking, as n/p increases, uncertainty in edge detection decreases, leading to smaller q-values. For example, in the county-level cancer study ($n = 2857$ and $p = 165$) the q-values around the target variables are on the order of 0.001, while in the breast cancer study ($n = 547$ and $p = 10{,}744$), the q-values in the immediate neighborhoods of the targets are on the order of 0.1.

2.5. The choice of r_{max} and $q^*_{\text{threshold}}$ is not particularly argued. Can you provide some references that suggest specific ranges to try for these two quantities?

We have expanded the Methods to clarify the roles of r_{max} and the q-value thresholds and to explain why there is no optimal choice for either parameter. In particular, we now emphasize that these parameters serve different purposes: the q-value thresholds control the level of uncertainty a user is willing to tolerate in the recovered structure, while r_{max} reflects the scope of the scientific question rather than statistical error tolerance. As with significance levels in hypothesis testing, the appropriate choice of q-value thresholds is inherently problem-specific and depends on the desired balance between discovery and conservatism; consequently, there is no oracle rule or generally recommended range that applies across applications. Similarly, the choice of r_{max} is guided primarily by domain knowledge and interpretability considerations. In some applications, relationships beyond a given radius are scientifically implausible or uninformative, allowing r_{max} to be fixed. In other settings, r_{max} can be set arbitrarily large, with the effective extent of the local graph determined automatically by the pathwise q-value threshold.

Reviewer 3

The article is coherent and well written. The presented research is sound and deserves wider attention. The manuscript articulates limitations of some of the existing methods, proposes a coherent solution and validates its performance with both an artificial simulation and two real datasets that yield potentially significant biological findings. The manuscript may benefit from the feedback below.

3.1. I suggest expanding the introduction and describing motivation in a bit more detail. The claim that existing methods often yield dense graphs is not supported very well as the only methods considered are variants of LASSO. Surely, not ALL existing methods yield equally dense graphs. If LASSO does have this undesirable tendency, can the problem be traced to poor regularization or is it inherent in the formulation?

We have substantially revised the Introduction to better motivate the problem of local graph estimation and clarify the limitations of existing approaches. In addition to lasso/regularization-based estimators, we now compare PFS to global and local versions of multiple constraint and/or mutual information-based procedures. While certain of these methods generally produce sparser graphs than regularized approaches, simulation and real-data results indicate that this sparsity often comes at the cost of reduced power, particularly in nonlinear or dense settings.

Regarding lasso-based methods, the tendency to yield dense graphs is inherent to the formulation rather than a consequence of poor regularization. In Section S2.1, we show that there is no choice of the regularization parameter λ that yields accurate local subgraph recovery in certain commonly encountered settings. This limitation applies to all approaches that ultimately rely on the graphical lasso in the final estimation step, motivating the need for methods that explicitly target local structure rather than relying on global regularization.

3.2. It seems there are two independent goals that are not articulated very well. The advantage of targeted estimation lies with computational efficiency for large datasets and does not directly address density or accuracy. Even for denser structures obtained via global estimation, given the Local Markov property, it's always possible to extract just the Markov Neighborhood (MN) of a target variable, simplifying interpretation significantly (unless MN itself happens to contain too many nodes). On the other hand, the improved accuracy of PFS is what addressed density and, as such, perhaps deserves a separate paragraph. Can this improvement in accuracy be extended to the global graph estimation?

Improved computational efficiency and accuracy are related in that they are both consequences of the local graph framework. Specifically, by focusing inference around target variables, the number of edges to be estimated is greatly reduced—especially in high-dimensions—lowering computational expense relative to global estimation methods. Simultaneously, concentrating inference near target variables allows for greater modeling flexibility and more precise estimation in regions of interest, leading to increased local accuracy compared to existing methods that treat all nodes uniformly. We have updated the Introduction to clarify this point.

Regarding Markov neighborhood (MN) estimation, a fundamental component of this work is that our goal is not to recover only the immediate neighborhood of a target variable, but rather to identify extended local structures such as paths and clusters. Recovering MNs alone is insufficient for this objective. Moreover, while it is always possible to extract a Markov neighborhood from an estimate of the global graph, this presupposes that the global estimator

reliably recovers the local neighborhood. Our mathematical results (Section S2) and empirical findings show that global methods do not reliably estimate local structures, including MNs, in many settings. We now address this in the third paragraph of Section 2.

In principle, the improved local accuracy achieved by PFS could be extended to global graph estimation. However, PFS is specifically designed to prevent estimation errors near the target variables from propagating throughout the graph. As a result, the estimated graph typically becomes increasingly sparse as one moves away from the targets, which may induce false negatives far from the region of interest. This behavior reflects the fact that local and global graph estimation target fundamentally different estimands. For this reason, we recommend existing global methods for global inference tasks and PFS for local graph estimation.

3.3. Figure 1e obscures the MN of the target variables, so it's difficult to directly assess how much improvement PFS actually delivers. Sometimes, a few unfortunate edges will fold the graphs on itself and make it impossible to read. There is no way to tell whether something like this may be going on here. The MN here seems to be comprised of roughly the same nodes as that of Figure 2, so it's not clear whether the improvement to the MN itself is significant. The same applies to most of the figures in the supplementary material (except for Figure S8). It would help if, at least, the dissimilarity between MNs of different graphs, e.g. the count of the same edges, were assessed somewhere in the manuscript.

Because MN estimation is precisely local graph estimation when $r = 1$, MN recovery is directly assessed via the reported true positive rates (TPRs) and false discovery rates (FDRs) at radius 1 in simulations. In applications, we now report the number of nodes identified as part of our over-representation analyses (Tables 1 and S10). Regarding Figure 1e: the fact that the figure obscures the MN of the target variables is precisely the point we intend to make with this illustration; namely, global estimation often produces dense graphs that obscure relevant local structure. As noted in our previous response, our principal objective is not MN estimation but rather to recover extended, target-specific local structures. In particular, two methods may estimate identical MNs yet return very different graphs beyond radius 1. This is apparent in the results of Section S10; for example, Figure S18 shows that while MNs of the target variables (top panels) remain relatively sparse, the corresponding radius-2 neighborhoods (bottom panels) become extremely dense, making extended local structure difficult to discern.

3.4. Local graph estimation (estimation of local structures around a group of target variables) is not exactly a new problem. It has been considered in the form of local MN estimation at least for directed graphs. Local MN estimation has been used as a way of dealing with performance limitations in a wide set of applications, including a variety of biological applications like GWAS studies. It would be advisable to include some discussion regarding what could be considered prior/similar work. It would be helpful if the manuscript considered a wider context. For example, does PFS offer something fundamentally different with respect to other local estimation methods? Can it be compared to non-LASSO techniques? In what way would estimating information-theoretic quantities like Mutual Information over the same extended structures be inferior to feature selection via PFS?

We agree that Markov neighborhood (MN) estimation, which is equivalent to local graph estimation when the radius $r = 1$, is an established problem. However, we are not aware of any MN estimation work that formalizes or directly addresses the problem of estimating local graphs of radius $r > 1$. As noted in our previous responses, our objective is to recover extended local structures, which requires $r > 1$ and therefore is not addressed by MN estimation alone.

Moreover, existing methods do not combine MN neighborhoods in a way that is simultaneously target-focused and provides false discovery control along edges and paths. We have revised the Introduction to emphasize these important points; see also Section S1.

Regarding how PFS fundamentally differs from existing methods: to our knowledge, PFS is the first graph estimation method that provides pathwise false discovery control, thereby enabling principled inference of extended local structures around target variables. PFS also gives edgewise false discovery control, with edges weighted by q-values, allowing uncertainty to be visualized directly in the estimated graph. These features are not available for existing graph estimation methods. We have revised the manuscript to make these contributions more explicit.

Regarding comparison to non-lasso techniques, including mutual information-based methods: we now compare PFS to seven such approaches in simulations and applications. In addition to lacking pathwise false discovery control, many of these methods scale poorly to high dimensions, as reported in Table S1. Additionally, constraint and mutual information-based methods often exhibit markedly less power than PFS in nonlinear or moderately dense simulation settings (Tables S3–S6). In applications, over-representation analyses of local graphs estimated by several of these methods show similar patterns (Tables 1 and S10).

3.5. Figure 2 could use more discussion. It reveals what could be at least some degree of "overfitting" and yet this is what PFS is supposed to control. For example 5 nodes (Mortality, Education, Poverty, Home Value, Income) form a completely connected graph where at least a few nodes are likely conditionally independent, e.g. Mortality and HomeValue given Income, or Education and Income given Poverty. Another fully connected structure (Incidence, PM2.5, TCE, SO2) appears on the left side of the figure. There are some questionable fully connected node triples scattered around the figure that further contribute to the overall density. Is the sensitivity too high? Do the user specified parameters need further adjustment? How can this be interpreted in terms of the simulation studies in S6.2? How stable are these findings under varying parameters?

We have revised the text to emphasize that, rather than claiming to eliminate all spurious edges, PFS controls the *expected proportion* of false discoveries in neighborhoods. Some spurious edges are therefore expected, the upside being that users can balance error control with discovery in order to identify plausible substructures within the system that can be validated via additional analyses, as we have done throughout this work.

One such example is the fully connected subgraph involving Incidence, $PM_{2.5}$, TCE, and SO_2 in Figure 2, which is supported by external evidence. As we state in the main text: "*Several of these [environmental exposures]—notably fine particulate matter ($PM_{2.5}$) and sulfur dioxide (SO_2)—directly connect to numerous compounds classified by the International Agency for Research on Cancer (IARC) as known or probable human carcinogens (Table S7). For example, $PM_{2.5}$ links to SO_2, which in turn connects to other pollutants such as ozone (O_3), carbon monoxide (CO), and nitrogen dioxide (NO_2), reflecting known atmospheric processes by which secondary pollutants are formed.*" Moreover, $PM_{2.5}$ and TCE are both classified as carcinogenic to humans (Group 1) by the IARC, lending further support to their connections to Incidence.

Several of the other suggested violations of conditional independence are possibly explained by the fact that PFS estimates a conditional independence graph (CIG) rather than a causal

DAG. Notably, relationships of the form $A \to B \leftarrow C$ in a DAG become fully connected triples in the corresponding CIG when the DAG is moralized.

We also emphasize that while some false positives are expected under any procedure that controls false discovery rates, simulation and application results throughout this work suggest that PFS provides a favorable balance between error control and discovery relative to mutual information- and regularization-based methods, which often underfit and overfit, respectively. In the case of the environmental and public health cancer study, for example, none of the mutual information methods (ARACNE, MMPC, or StablePC) identifies edges between Incidence and $PM_{2.5}$ or between Incidence and TCE, while most regularization-based methods produce extremely dense radius-2 local graphs (Section S10.1). This behavior mirrors the simulation results reported in Sections 3 and S5, where regularization methods tend to overfit, constraint and mutual information-based methods tend to underfit, and PFS tends to identify a relatively high number of positives while keeping false discoveries under control (but not zero).

Finally, the stability of the PFS estimate under varying q-value thresholds can be observed directly from the graphs: in Figure 2, for example, reducing the q-value threshold around Mortality to 0.007 would eliminate edges between Mortality and Poverty and between Mortality and Smoking (the edge between Mortality and Incidence would remain since Incidence's q-value threshold exceeds 0.008). This would lead to a sparser graph. More generally, as thresholds are tightened, edges are removed without qualitative reorganization of local neighborhoods; as thresholds are relaxed, additional nodes gradually enter the graph.

3.6. As a core component, IPSS should be at least briefly formulated somewhere in the manuscript. What appears in the supplementary material is not quite sufficient and IPSS is treated as a black-box. This is not a well enough known method, particularly the variant used in this paper, to be treated so casually.

We have revised the manuscript to include a dedicated Methods subsection describing integrated path stability selection (IPSS). References to the original IPSS papers are also provided for readers interested in further technical details.

Remarks on code availability

3.7. The installation instructions could be improved if step-by-step instructions, starting from a clean python installation to the test-run, were added. I was able to pip-install the code, but, unfortunately, not all package dependencies were resolved. Attempting to execute simulation.py and run_pfs.py revealed missing packages – pandas, pyqt5, rpy2, etc... It might be helpful to list the whole dependency tree on the main page (including a working installation of R interfaced with python). The datasets are as described in the manuscript.

Thank you for testing the code. We have revised `requirements.txt` to list all dependencies required to run the scripts in the repository. We have also clarified in the `README` that certain comparison methods rely on R packages accessed via `rpy2`, and therefore require a working R installation available on the system path.

Reviewer 4

This study introduces Pathwise Feature Selection (PFS), a computational method for local graph estimation—focused on inferring sparse and interpretable subgraphs around target variables from

high-dimensional data. PFS is built on conditional independence graphs, using integrated path stability selection (IPSS) for nonparametric q-value estimation. It iteratively expands neighborhoods from target variables and tracks cumulative path uncertainty via summed q-values. Unlike traditional full-graph methods that yield dense, uninterpretable results, PFS recovers sparse, biologically meaningful local subgraphs, handles nonlinear dependencies and mixed variable types, and scales to high-dimensional data. While PFS has been validated in two cancer studies, providing a targeted, interpretable approach for local graph inference, I have a few questions to be addressed that are listed below.

Major concerns

4.1. The authors claim existing graph estimation methods yield dense, less interpretable graphs prone to false discoveries, so PFS uses a user-specified radius to constrain output to the target variable's radius-hop neighborhood, ensuring sparsity for interpretability and false positive control. However, real-world local graphs around targets (e.g., in cancer onset or gene regulation) may not be sparse—multiple relevant factors could exist, and forced sparsity may cause false negatives. Key questions remain unaddressed: How does PFS balance its emphasis on sparsity and false positive control while minimizing false negatives? Moreover, in the simulated test, the ground-truth local graph around the target is inherently sparse, potentially masking false negative issues; can PFS still effectively resolve local structure when the ground-truth around the target is dense?

Thank you for raising this concern. We emphasize that PFS is designed to control false discoveries, not to enforce sparsity. In particular, the user-specified radius determines the scope of the local structure explored around the target variable, but it is not what ensures error control. False discovery control at any radius follows from Theorem S3.2, which makes no assumptions about graph sparsity. Consequently, if the true local neighborhood at radius r is dense, PFS does not necessarily suppress those edges.

To evaluate the extent to which sparse ground-truth settings may mask false negatives, we have added three new simulation studies in which the radius-1 neighborhood of the target contains 20 variables and the overall graph is substantially denser than in our original experiments. The results, reported in Tables S4, S5, and S6, show that in both linear and nonlinear regimes PFS consistently achieves competitive or superior true positive rates relative to other methods while maintaining control of false discoveries. In the dense linear and nonlinear studies with $n = 500$, PFS attains the highest TPR of any method in the radius-1 neighborhood of the target variable. These results demonstrate that PFS does not rely on sparsity of the true local graph to achieve good power. Rather, it adapts to dense local structure when present, recovering many true neighbors while avoiding the explosion of false positives observed in other graph estimators.

4.2. The introduction outlines the limitations of existing high-dimensional data analysis methods, such as the inability to handle all variables simultaneously, lack of interpretability, and restrictive assumptions. However, it remains unclear how the authors' chosen framework—graphical estimation—addresses these issues. A more compelling justification is needed. Further comparative testing is needed to evaluate the performance of the graph estimation framework against other frameworks (such as deep learning and machine learning based frameworks) in terms of interpretability, computational efficiency, and preservation of genuine signals.

We have substantially revised the Introduction to explain why graph estimation is the appropriate framework for our stated objective. Specifically, we now begin by explicitly stating that our

goal is to learn *local structure*—such as neighborhoods, clusters, and pathways—around target variables, rather than prediction or unsupervised learning. We then explain why other widely used frameworks for high-dimensional and multimodal data analysis are misaligned with this objective and why graph estimation is appropriate for our goals.

We also emphasize that PFS is itself a machine/deep learning-based framework, not an alternative to them. Specifically, PFS provides a principled, theoretically supported approach for transforming importance scores from any machine/deep learning method into valid q-values for edges and paths, thereby enabling formal reasoning about local structures. In this work, we use scores from gradient boosting and random forests because deep learning models are substantially more difficult to tune, more computationally expensive, and are known to underperform these tree-based methods in the moderate sample regimes that we consider (Shwartz-Ziv and Armon, 2022; Grinsztajn et al., 2022).

4.3. The core PFS methodology relies on IPSS q-values to determine edges and control false discoveries, yet the advantages of using IPSS q-values in graph estimation scenarios compared to other statistical measures remain unclarified.

First, we clarify that PFS is compatible with any method that computes q-values, so users are not restricted to IPSS (Algorithm 1). That said, we have expanded the discussion in both the main text and Methods to clarify the advantages of using IPSS-derived q-values over other statistical measures that could be used for edge selection. We explain that, unlike other commonly used measures such as raw variable importance scores, q-values quantify uncertainty and support explicit false discovery control. Additionally, IPSS computes q-values from arbitrary variable importance scores and therefore supports nonlinear relationships and does not rely on distributional assumptions, explaining PFS's favorable performance in the nonlinear simulations. IPSS also inherits robustness from repeated subsampling, making its q-values less sensitive to data perturbations or cohort variability. Relative to earlier stability selection approaches, IPSS yields more precise q-values and tighter false discovery control. Further empirical comparisons to alternative feature selection methods are reported in the IPSS papers, which we reference in the main text and Supplement (Melikechi and Miller, 2025; Melikechi et al., 2025).

4.4. When conducting benchmarking, the authors only compared PFS with existing graph estimation-based methods. However, given that numerous other methods have been developed for multi-omics studies and high-dimensional data analysis, for example, by Uyar et al.(`https://pubmed.ncbi.nlm.nih.gov/40940333/`) and by Argelaguet et al. (`https://pubmed.ncbi.nlm.nih.gov/32393329/`), the performance of PFS should also be evaluated against these alternative approaches on the same datasets.

We agree that there are a broad range of methods for analyzing high-dimensional multimodal data. However, many of these approaches are not comparable to PFS because they target fundamentally different objectives. We have revised the Introduction to clarify the inferential scope of local graph estimation/PFS and to distinguish its focus on targeted structure learning from other task in high-dimensional or multimodal data analysis such as prediction or unsupervised learning. See, in particular, the second paragraph of the updated Introduction.

We did, however, apply MOFA+ to the TCGA breast cancer dataset analyzed in this work, which contains four modalities: genes, proteins, microRNAs, and clinical variables. The method issued the following warning: "*Warning: some view(s) have less than 15 features, MOFA won't be able to learn meaningful factors for these view(s)*," indicating that results for the clinical

modality, which contains the three target variables of interest, are unreliable. Consistent with this warning, two of the three clinical targets (patient status and pathologic stage) had near-zero loadings across all factors, and none of the targets shared a common leading factor. This behavior reflects the different objective of latent factor models rather than a limitation of MOFA+ itself. Accordingly, we do not report these results in the paper.

4.5. The method requires users to set a pathwise q-value threshold. The article mentions using a value of 0.4 for this threshold—what is the justification for selecting this specific value? A method should be provided to evaluate the performance of different pathwise q-value thresholds.

The pathwise q-value threshold of 0.4 in Figure 1 is chosen to illustrate how PFS works and the advantages of pathwise false discovery control; it is not intended as a generally recommended or default value. Specifically, in that example, the randomly generated data are such that the two falsely identified edges in the immediate neighborhood of the target have q-values 0.33 and 0.36, respectively, whereas the two true edges have substantially smaller q-values of 0.14. Setting the threshold to 0.4 illustrates that, although these false edges are identified at this level, PFS assigns them higher uncertainty than the true edges and therefore continues to expand the local graph beyond the true edges but does not propagate beyond the false ones. At more conservative thresholds, for example 0.2, the false edges (X_1, X_{36}) and (X_1, X_{86}) would not appear in the local estimate at all.

More generally, we have revised the Methods section to emphasize that there is no universally optimal choice of pathwise q-value threshold, as it encodes the level of uncertainty a user is willing to tolerate and is therefore inherently problem-specific. As with significance levels in hypothesis testing, different thresholds reflect different tradeoffs between discovery and conservatism, and no oracle rule exists for selecting them. Rather than proposing a fixed threshold or a formal tuning procedure, PFS is designed to explicitly quantify this tradeoff and enable user-control. In practice, users may assess stability, interpretability, and scientific plausibility of the recovered structures across a range of thresholds, analogous to sensitivity analyses commonly performed for significance levels (see also our response to Comment 2.5).

4.6. In the discussion section, the authors mention that PFS demonstrates high generalizability; however, its current validation has only been conducted on public health datasets and bulk multi-omics datasets, which appears insufficient. The authors should extend the testing of PFS to more heterogeneous datasets, such as single-cell and spatial multi-omics datasets.

We have expanded the empirical evaluation to include two additional settings that are statistically and scientifically distinct from the original environmental/public health and breast cancer applications. First, we analyze data from the Human Connectome Project comprising neuroimaging-derived phenotypes together with behavioral measures. Second, we include a single-nucleus RNA-sequencing study of Alzheimer's disease, providing a cell-type-specific single-cell application with over 10,000 variables per analysis, where each analysis corresponds to one of three distinct cortical cell types. In both applications, external validation through literature review and over-representation analyses indicates that the local structures identified by PFS are scientifically interpretable and consistent with established domain knowledge. Together with the environmental and bulk multi-omics studies, these additions broaden the empirical evidence supporting the applicability of PFS across multiple domains and data modalities.

Minor concerns

4.7. A Methods section should be added to the main text to outline the key steps and rationale of the proposed algorithm.

We have added a Methods section to the manuscript. It includes a subsection that explicitly outlines the proposed algorithm, Algorithm 1, which appears in Section 2 of the main text. This subsection provides a concise description of the inputs and outputs of PFS, the roles of the neighborhood and path-level q-value thresholds, and the pathwise stopping criterion motivated by Theorem S3.2. We also include a clear definition of the lightest-path quantity used in Algorithm 1 and explain how it governs the iterative expansion of the local graph.

4.8. Figure 1c and 1d are not cited or described in the main text.

Figure 1 is cited in the main text, and panels (c) and (d) are described in the figure caption. We intentionally rely on the caption to explain individual panels in order to keep the main text focused and avoid unnecessary repetition. We believe this presentation is standard and sufficient for readers to interpret the figure.

4.9. Given that Figure 1e and Figure 2 present results from different methods applied to the same dataset, I suggest consolidating them into a new, combined Figure 2.

Figure 1e is intended to illustrate a common pitfall of global graph estimation methods, namely that dense global estimates can obscure local structure around target variables. By contrast, Figure 2 is designed to highlight the behavior of PFS in recovering interpretable local structure. Although the two figures use the same dataset, they serve distinct conceptual purposes: Figure 1e motivates the need for targeted local graph estimation methods, while Figure 2 demonstrates the practical benefits of PFS for this task. Consolidating the figures would obscure this distinction and weaken the narrative separating motivation from methodological evaluation.

4.10. The process for generating the simulated data used in Figure 1 should be described in the Methods section.

Thank you for this suggestion. The Methods section now includes a subsection, *Simulation design for Figure 1*, which describes the data-generating process used in panels (a) through (d).

Remarks on code availability

4.11. The software package can be installed.

Thank you for checking this.

References

Léo Grinsztajn, Edouard Oyallon, and Gaël Varoquaux. Why do tree-based models still outperform deep learning on typical tabular data? *Advances in Neural Information Processing Systems*, 35: 507–520, 2022.

Omar Melikechi and Jeffrey W Miller. Integrated path stability selection. *Journal of the American Statistical Association*, pages 1–11, 2025.

Omar Melikechi, David B. Dunson, and Jeffrey W. Miller. Nonparametric IPSS: fast, flexible feature selection with false discovery control. *Bioinformatics*, 41(5):btaf299, 2025.

Ravid Shwartz-Ziv and Amitai Armon. Tabular data: Deep learning is not all you need. *Information Fusion*, 81:84–90, 2022.

Reply to reviews for
"Local graph estimation: Interpretable network discovery for complex data"

We thank the Associate Editor and reviewers for their constructive feedback. Below we provide point-by-point responses to the remaining comments.

Reviewer 1

The authors have addressed all concerns to my satisfaction.

Thank you for taking the time to review our manuscript and for your valuable feedback.

Reviewer 2

The authors have comprehensively addressed all my previous concerns. Rather than simply providing textual justifications, they have conducted substantial new analyses that significantly elevate the methodological and empirical solidity of the paper. I am particularly satisfied with the following revisions:

Quantitative Validation: The inclusion of formal Over-Representation Analyses (ORA) for the real-world datasets, alongside the empirical validation using the STRING database (Tables S9 and S10), completely resolves my initial concern regarding potential confirmation bias. Moving from a qualitative "gene-by-gene" narrative to a statistically sound module-based evaluation greatly enhances the biological interpretability and reliability of the method.

Performance Metrics and Sample Size: The expanded simulation studies explicitly reporting TPR and FDR across different network topologies (dense/sparse, linear/non-linear) provide the quantitative transparency I requested. Furthermore, the new analysis evaluating the performance as a function of the sample-to-feature ratio (Figure S2) effectively demonstrates the algorithm's consistency and false discovery control across different dimensional regimes.

While the selection of parameters such as r_{max} and q^* relies on theoretical thresholds and user-defined tolerance rather than offering an automated data-driven heuristic, I acknowledge that this is a standard constraint in this class of statistical frameworks and accept their rationale. Overall, the

authors have done an excellent job refining their work. I have no further critiques and recommend the manuscript for publication in Nature Communications.

Thank you for your time and thoughtful feedback. We are glad that the revised manuscript addresses your previous concerns and are grateful for your many helpful suggestions.

Reviewer 3

I thank the authors for addressing almost all of the original concerns. A few remaining outstanding issues remain:

3.1. The first paragraph on page 2: Now the text seems to suggest that the second class of approaches (including information-theoretic methods) scales poorly. This is, of course, not the case and should be reworded. First of all, not all methods from the second class are the same, and this is quite evident in Table S1 of the supplement (ARACNE is actually almost always faster than PFS). So, only some methods scale poorly, which must also be highly implementation-dependent. Second, methods requiring matrix operations will generally scale worse than scoring/testing methods by design. Any method that needs LAPACK solvers for searching the solution space will usually be more computationally demanding and have greater scaling limitations compared to some serial alternatives.

We agree that ARACNE is a notable exception in terms of scalability among the methods we tested in this second class. We have revised the paragraph to avoid suggesting that the entire class scales poorly or that performance is independent of implementation. The text now states that popular implementations of many of these methods scale poorly with dimension, citing both prior work and empirical results in our manuscript. For ease of reference, we elaborate on prior work that supports this claim below.

In an extensive comparison study, Yu et al. (2020) find that IAMB and FastIAMB are among the fastest out of 18 methods in this second class, including both constraint- and score-based approaches. In our experiments, IAMB and FastIAMB scale substantially worse than alternatives outside of this class, namely PFS and regularized approaches. Regarding parent and child (PC) algorithms, Ling et al. (2020) state: "*[S]ince standard PC discovery algorithms, MMPC and HITON-PC, need to perform an exhaustive subset search within the currently selected variables for PC discovery, the existing local causal structure learning algorithms are computationally expensive or even prohibitive when the size of the PC set of the target becomes large.*" Wang et al. (2019) make a similar observation, stating that many of these methods are "*computationally demanding and limited to relatively small-scale systems.*"

3.2. Second paragraph on page 2: Prior work in local graphs estimation needs more attention. Even though the local MN estimation is often restricted to the immediate neighborhood, the extension of ANY local procedure to the estimation of the optimal embedding envelope of arbitrary size is a natural next step that has been taken before more than once (e.g. Gao (2017) Local-to-Global BN Structure Learning, etc.). So, what this paragraph seems to claim, due to its unrestricted generality, is simply not true. If the listed limitations are attributable to

specific packages (e.g. bnlearn) then the claims about existing MN estimation strategies should be restricted to these specific methods/packages to avoid making overly general claims.

Thank you for pointing us to this prior work. We agree that neighborhood procedures can be combined to estimate subgraphs of arbitrary size; both the previous and current versions of our manuscript examine several methods that take this approach, including nodewise lasso, HPC(L), MMPC(L), SIHPC(L), IAMB(L), and FastIAMB(L) (see, for example, Tables S1–S6). However, while such extensions are possible, their use for estimating extended local structures, such as paths or local graphs of radius-r around target variables when $r > 1$, has received very little attention in the literature. The Gao et al. paper, for example, focuses solely on recovering the full graph from neighborhood estimates and evaluates performance using global structure learning metrics. In contrast, our work focuses explicitly on recovering subgraphs around target variables at different radii. More importantly, theoretical results for existing local-to-global-type approaches often rely on strong assumptions, such as infinite samples—as assumed in Gao et al. (2017)—or the ability to perfectly identify conditional independence relations from data (Wang et al., 2014). To our knowledge, existing work does not establish finite-sample false discovery control at the level of paths. In this sense, the limitations of existing approaches with respect to local graph estimation are primarily methodological and theoretical rather than implementation or package-based. We have revised the paragraph to clarify these points.

3.3. Page 5 last paragraph: The claim that PFS achieves the "highest TPR among all methods in both linear and..." contradicts the tables in the supplement where some methods seem to outperform PFS in power for linear setting (G/NLASSO, ARACNE, HPC, etc. in Table S2; G/NLASSO in Table S3; NLASSO, BNWSL, GFCSL, etc. in Table 4). Where PFS does outperform other methods in power is specifically the nonlinear setting, which should really be brought out from the start. Perhaps, a robust balance between TPR and FDR across other scenarios, but certainly not "the highest TPR". If I may suggest author's own words from the rebuttal item 3.5: "this work suggests that PFS provides a favorable balance between error control and discovery".

Thank you for catching this. We have revised the main text to accurately reflect the results in Tables S2–S6. Specifically, the previous claim is now replaced by: "*PFS performs especially well in nonlinear settings, achieving the highest TPR among all methods at radii 1, 2, and 3 for both sparse and dense graphs. Furthermore, its consistent balance between discovery and error control across the dense settings indicates that PFS neither incurs excessive false negatives nor depends on sparsity for good performance.*"

Remarks on code availability

3.4. I was able to install the code and run a few scripts (e.g. run_pfs.py). But unfortunately for some scripts either the paths are garbled or something else

FileNotFoundError: [Errno 2] No such file or directory: '../data/cleaned_data/cleaned_data.csv'
ImportError: cannot import name 'max_cor_response' from 'localgraph'

I did not have the time to troubleshoot this and could not reproduce the results. Also, the "results" folder described in README is missing and is never created.

Thank you for running the code and for the detailed feedback. We have fixed all three issues:

- `FileNotFoundError`: Scripts previously constructed paths relative to the working directory. We have updated all `run_pfs.py` and `run_methods.py` scripts to anchor paths to the script's

own location using Python's `pathlib`, so they run correctly regardless of the working directory.

- `ImportError`: This was due to a version mismatch with the `localgraph` package. We have pinned `localgraph==0.1.4` in `requirements.txt`, which resolves all imports.
- Missing `results/` folders: These were excluded from the repository via `.gitignore`. We have added them to the repo and updated `.gitignore` accordingly.

We have verified that the code runs end-to-end on two examples: `simulations/illustration.py` ($\approx$ 30 seconds) and `applications/hcp/run_pfs.py` ($\approx$ 5 minutes). A Quickstart section has been added to the README to help users execute these scripts.

Reviewer 4

The manuscript has been carefully revised to clarify the scope and contributions of the work. The inclusion of additional simulation studies and a new single-nucleus RNA-sequencing application strengthens the empirical validation. However, two specific concerns remain regarding the methodological evaluation and data presentation.

Major concern

4.1. An ablation study is necessary to empirically validate the proposed algorithmic combination. Specifically, the authors should keep the PFS framework constant while substituting the underlying q-value generator to evaluate the empirical performance of IPSS q-values specifically within the PFS framework. While the authors provided a theoretical explanation of the advantages of IPSS q-values—such as their nonparametric nature and explicit false discovery control—the core of the original concern remains unaddressed. The cited IPSS literature evaluates its performance strictly in single-target feature selection scenarios. In contrast, PFS relies on an iterative, pathwise expansion where uncertainties are summed along multi-hop network paths. It cannot be assumed that a metric optimized for single-step selection remains optimal under cumulative pathwise error propagation.

Thank you for this suggestion. We have added five simulation studies to the Supplement (Section S5.4) in which all PFS parameters other than the underlying q-value construction method are held fixed. Specifically, we compare PFS with IPSS-based q-values to versions using the Benjamini–Hochberg and Benjamini–Yekutieli procedures, as well as multiple variants of model-X knockoffs. Tables S7–S11 show that PFS with IPSS achieves a favorable balance between TPR and FDR in a variety of settings, indicating that its advantages persist under cumulative pathwise error propagation.

Minor concern

4.2. Figure 1e and Figure 2 should be consolidated into a new Figure 2. Figures 1a-d, based on simulated data, sufficiently establish the research motivation by illustrating the differences between global graph estimation methods and PFS. Since Figure 1e and Figure 2 visualize the same real-world dataset, combining them offers a direct side-by-side comparison that highlights the practical advantages of PFS in real-world scenarios and enhances readability without disrupting the logical flow.

We appreciate this suggestion, but still prefer the current organization. While Figures 1e and 2 use the same dataset, their roles in the manuscript are distinct: Figure 1e motivates local graph estimation by illustrating a common limitation of existing global methods on real data (complementing the simulation-based examples in Figure 1a–d), whereas Figure 2 is the primary result of Section 4, around which that section is organized. Combining these figures would result in a single figure serving different purposes across separate sections, disrupting the narrative progression from motivation to key results and reducing the legibility of the local graph in Figure 2.

References

Tian Gao, Kshitij Fadnis, and Murray Campbell. Local-to-global Bayesian network structure learning. In *International Conference on Machine Learning*, pages 1193–1202. PMLR, 2017.

Zhaolong Ling, Kui Yu, Hao Wang, Lei Li, and Xindong Wu. Using feature selection for local causal structure learning. *IEEE Transactions on Emerging Topics in Computational Intelligence*, 5(4): 530–540, 2020.

Changzhang Wang, You Zhou, Qiang Zhao, and Zhi Geng. Discovering and orienting the edges connected to a target variable in a DAG via a sequential local learning approach. *Computational Statistics & Data Analysis*, 77:252–266, 2014.

Lingfei Wang, Pieter Audenaert, and Tom Michoel. High-dimensional Bayesian network inference from systems genetics data using genetic node ordering. *Frontiers in Genetics*, 10:1196, 2019.

Kui Yu, Xianjie Guo, Lin Liu, Jiuyong Li, Hao Wang, Zhaolong Ling, and Xindong Wu. Causality-based feature selection: Methods and evaluations. *ACM Computing Surveys (CSUR)*, 53(5):1–36, 2020.

Reply to reviews for
"Local graph estimation with pathwise false discovery control"

Reviewer 3

I thank the authors for addressing the concerns and introducing appropriate changes into the manuscript.

Thank you for taking the time to review our manuscript and for your helpful suggestions.

Remarks on code availability

3.1. The code repository is now missing the "requirements.txt" file, so there is no way to install it according to the repository instructions. I would advise that the installation section on the intro page should precede the "quick start" section.

Thank you for checking the repository. The `requirements.txt` file was accidentally excluded from the repository by a `.gitignore` rule; we have corrected this. We have also restructured the README so that the Installation section now precedes the Quickstart section.

Reviewer 4

The authors have addressed all my comments, and I have no further concerns.

Thank you for your time and thoughtful feedback across both rounds of review.
